# DNA repair and recombination in higher plants: insights from comparative genomics of arabidopsis and rice

**DOI:** 10.1186/1471-2164-11-443

**Published:** 2010-07-21

**Authors:** Sanjay K Singh, Sujit Roy, Swarup Roy Choudhury, Dibyendu N Sengupta

**Affiliations:** 1 Department of Botany, Bose Institute, 93/1 Acharya Prafulla Chandra Road, Kolkata 700 009, India; 2 Department of Chemistry, Protein Chemistry Laboratory, Bose Institute, 93/1 Acharya Prafulla Chandra Road, Kolkata 700 009, India

## Abstract

**Background:**

The DNA repair and recombination (DRR) proteins protect organisms against genetic damage, caused by environmental agents and other genotoxic agents, by removal of DNA lesions or helping to abide them.

**Results:**

We identified genes potentially involved in DRR mechanisms in *Arabidopsis *and rice using similarity searches and conserved domain analysis against proteins known to be involved in DRR in human, yeast and *E. coli*. As expected, many of DRR genes are very similar to those found in other eukaryotes. Beside these eukaryotes specific genes, several prokaryotes specific genes were also found to be well conserved in plants. In *Arabidopsis*, several functionally important DRR gene duplications are present, which do not occur in rice. Among DRR proteins, we found that proteins belonging to the nucleotide excision repair pathway were relatively more conserved than proteins needed for the other DRR pathways. Sub-cellular localization studies of DRR gene suggests that these proteins are mostly reside in nucleus while gene drain in between nucleus and cell organelles were also found in some cases.

**Conclusions:**

The similarities and dissimilarities in between plants and other organisms' DRR pathways are discussed. The observed differences broaden our knowledge about DRR in the plants world, and raises the potential question of whether differentiated functions have evolved in some cases. These results, altogether, provide a useful framework for further experimental studies in these organisms.

## Background

The integrity of genomes of all living organisms is continuously being challenged by different environmental agents and metabolic by-products. Consequently, evolution has provided organisms with several DNA repair and recombination (DRR) pathways to remove or to tolerate lesions in their genetic material and maintain the integrity of genome. Thus DRR is not only a fundamental cellular process for protecting cells against the damage, but is also indispensable to ensure faithful transmission of genetic information from one generation to the next. In fact, these partly redundant machineries have at least two important contrasting roles in evolution, escorting the genome, and allowing for a certain level of mutations during evolution. The decisive balance of these two activities is perhaps the best reason for the high levels of conservation observed in DRR related proteins, even across the three domains, Bacteria, Archaea and Eukarya [1-3].

The effect of environmental change on ecosystem scales responses and on the life processes of individual organisms has been a major environmental issue for the last three decades. Understanding of how species may evolve in response to environmental changes remains relatively unclear, particularly for plants. Plants, because of their intrinsic immobility, are constantly exposed to various environmental agents and endogenous processes that impose damage to DNA and cause genotoxic stress, which can reduce plant genome stability, growth and productivity. Like any other organisms plants employ a wide variety of strategies to either reverse, excise, or tolerate the presence of DNA damage products. Although repair and damage tolerance mechanisms have been thoroughly described in *E. coli*, *Saccharomyces cerevisiae*, rodents and human, surprisingly little is known about these processes in plants. However, in recent years, there has been increased interest in plant DRR and in using plants as models for understanding the under lying mechanism of DRR [4].

Our research interest lies mainly with understanding the coordination between replication and repair machinery in higher plant genome with special emphasis on the role of single subunit, short gap- filling family-X DNA polymerase orthologues in plant DNA replication and repair (DNA polymerase λ) [5-9]. Previously few attempts have been made to study the different repair pathway in plants at genome level but they were limited to either gene identification or single pathway only [10,11]. Recent study has reported in detail about the core DNA replication machinery in rice and *Arabidopsis *[12]. Previously, we carried out an *in silico *analysis to study the sequential, structural and phylogenetic features of BRCT (breast cancer susceptibility C-terminus) domain in higher plant genome and investigated the distribution of this module in various proteins in higher plants in relation to the functional significance of these proteins in plant DNA damage and repair [13]. However detailed knowledge about the sequential, structural and evolutionary properties of the genes involved in plant DRR events is still very limited.

In this article, we have made an attempt to systemically analyze the genomes of the two fully sequenced and well explored plants, *Arabidopsis *(a dicot) and rice (a monocot), to investigate the presence and evolution of genes known to be involved in DRR in other organisms. We have found that some repair machinery is very well conserved in plant genomes whereas others have diverged more rapidly. In addition, several genes involved in different repair pathways are found to be duplicated in both the genomes. To further gain insight into plant DRR components, we have combined published experimental information with our own bioinformatics analysis of genomic sequence data. We observed that the genes involved in DRR are, in general, part of the cell core metabolic pathways and showed significant similarity in different genomes while intriguing differences indicating biological diversity in plant responses to DNA damage. Overall this *in silico *study provides important information regarding DRR pathways in plants and represents a useful starting place for further research on the functional characterization of the proteins involved in plant DNA metabolism and the evolution of DRR genes in higher plant genomes.

## Results and discussion

### Identification of DRR genes in rice and *Arabidopsis*

In order to study the conservation and evolution of DRR pathways in rice (*Oryza sativa*) and *Arabidopsis *(*Arabidopsis thaliana*) genomes, we examined the conservation of 229 and 223 gene models in *Arabidopsis *and rice respectively. Detail about every gene and number of paralogs present in *Arabidopsis *and rice genomes are tabulated in additional file 1 while the locus IDs and relevant plant literature (if available) is listed in additional file 2. The genomes of *Arabidopsis *and rice were searched for the presence of genes known to act in the metabolism of DNA lesions, mostly in human, *Saccharomyces cerevisiae *and *E. coli*. When a gene was not found, it indicates either that the gene was absent in the considered organism or that the sequence was too varied to be recognized using our criteria. Later on, genes of rice and *Arabidopsis *were also queried against each genome to check the consistency of the genes. Sequences were also manually checked for the presence of any wrong annotations. Few probable wrong annotations were found. For example, BLAST analysis revealed three loci, LOC_Os02g54280, LOC_Os02g54170 and LOC_Os02g54290, with significant hits for AtBRU1 in rice, a gene acts as a link between responses to DNA damage and epigenetic gene silencing in *Arabidopsis*. We discarded LOC_Os02g54290 because it did not contain the BRU1 specific domain. LOC_Os02g54170 was annotated as BRUSHY 1 while LOC_Os02g54280 was annotated as retrotransposon protein. CDD analysis has shown that LOC_Os02g54280 contain the BRU1 specific conserved domains, tetratricopeptide repeats (TPRs) and leucine-rich repeats (LRRs), with few additional domains like RnaseH (cd06222). LOC_Os02g54170 has shown to contain only LRRs and lack TPRs. Thus the possibility of wrong annotation for LOC_Os02g54280, as like LOC_Os02g54290, was high as it was highly similar to AtBRU1 (1.1e-140, 41.0% identity and 67.3% similar) and also contained all conserved domains. The identified DRR genes were found to be distributed on all the rice (3 to 17%) and *Arabidopsis *(12 to 30%) chromosomes. In rice, the highest number of DRR genes (17.48%) was predicted on chromosome 1, the longest chromosome in the rice genome. Similarly in *Arabidopsis*, the longest chromosomes 1 and 5 of *Arabidopsis *have been found to harbour the highest number of genes involved in DRR (29.91% and 23.21% respectively) (Additional file 3). Apparently, the distribution of DRR genes in terms of their involvement in different pathway seems uniform, however a strong bias towards clustering of genes involved in same pathway on different chromosome deserve special mention (Additional file 4).

In order to facilitate understanding of the major similarities and differences between two genomes, genes have been classified as: 1-Excision repair [base excision repair (BER), nucleotide excision repair (NER) and mismatch repair (MMR)]; 2- Double-Strand Break Repair [homologous recombination (HR) and non-homologous end joining (NHEJ)] and 3- Other DNA repair related genes. In the third category, we have assigned all those genes which are not catalogued in KEGG pathway database as the part of main DRR pathways.

### Conservation of pathway

**Figure 1:**
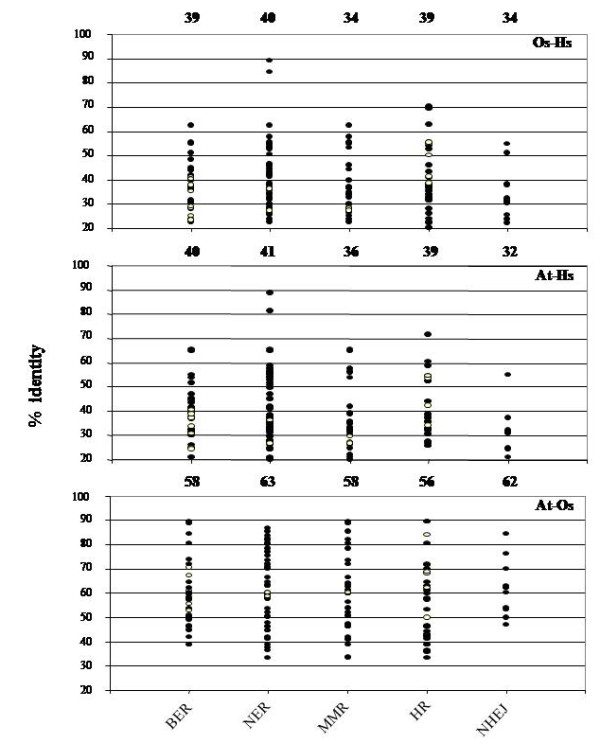
**Conservation of different DNA repair pathways**. Values of Smith-Waterman identity scores for conserved proteins in core DRR pathways. Each dot corresponds to a pairwise alignment between a protein and its orthologs. Eukaryotes specific proteins are represented by dark circles while prokaryotes specific proteins are represented by white circles (see text). Average identity of each pathway is indicated above the 100% line.

We next investigated the degree of conservation of DRR pathways. Conservation of DNA safeguarding pathways during evolution was calculated by the average conservation in amino acids and presence/absence of proteins belonging to a given pathway (Figure 1, Additional file 1). Component of different pathways have been defined according to KEGG pathway database. Pathway conservation was measured in three different categories: conservation between rice and human proteins (Figure 1a), conservation between *Arabidopsis *and human proteins (Figure 1b) and conservation between *Arabidopsis *and rice proteins (Figure 1c). Plants possess few prokaryotes and *Saccharomyces cerevisiae *specific DRR proteins also therefore *E. coli *and *Saccharomyces cerevisiae *specific proteins were used to compare the conservation level with its plants counterparts. Thus *E. coli *and *Saccharomyces cerevisiae *proteins replaced the human proteins in plant-human protein conservation categories and represented as white circles (Figure 1a and 1b). Proteins which are involved in several pathways were also included in each pathway to calculate the average conservation score. The best example was the MRN complex, classified in both NHEJ and HR pathway. In addition to the presence of several interconnections, pathway such as NER was more conserved than others in terms of amino acid identity. All genes of NER pathway revealed a high degree of sequence similarity with their counterparts present in other genomes (Figure 1). In human and plant pairs, NER, BER and HR pathways were very close in terms of identity of amino acid while in *Arabidopsis*-rice pair, NER and NHEJ pathways were closer to each other, while considering the characteristics of amino acid identity.

In terms of absence or presence of a particular protein involved in a given pathway, NER and MMR were found to be most conserved pathways where merely all components were present while NHEJ and BER were least conserved pathways of genome maintenance. All components of both sub-pathways of NER, transcription-coupled repair (TCR) and global genome repair (GGR) pathways were well conserved in both genomes except the xeroderma pigmentosum complementation group A (XPA) gene which is involved in the damage-recognition step of the NER processes, while all genes which are reported to be involved in eukaryotic MMR pathway are present in plants. Difference in term of copy number of genes as seen in BER and NER pathway was not found in MMR specific proteins may suggest the universal and utmost functional importance of this pathway. It was also found that all subunit of a multimeric protein were not conserved with similar rate during evolution. For example, POLD1 subunit of Delta-type DNA polymerase holoenzymes (*pold*) was always more conserved (At-Hs -54.1%, Os-Hs-55.4%) than the other subunits while POLD3 subunit was always least conserved (At-Hs -25.1%, Os-Hs -21.4%) (Figure 1). Rbx1, an ubiquitin ligase, was the most conserved gene in plant DRR pathways. Plants Rbx1 genes showed more than 80% identity to its human homolog. PCNA, DMC and RAD51A were the other much conserved proteins which showed more than 60% identity at amino acid level in plant-human pair. POLD3, PRKDC, RPA3, XRCC4 and NBS1 proteins were the least conserved genes in plant-human pair which shared less than 25% amino acid. In terms of difference in number of paralogs, MMR and NHEJ were much conserved. In both pathways, similar number of paralogs was found in *Arabidopsis *and rice genomes except few exceptions like MRE11 and FEN1. This result may suggest that MMR and NHEJ pathway have been naturally preserved as such through out plant genome evolution. Our analysis suggested that plant DRR machinery is more closely related to human as compared to yeast because plants retain more mammalian homologs than the yeast counterparts.

Plants homologs of bacterial DRR gene have very low sequence similarity but retention of conserved domain probably suggested the functional conservation of those proteins. Bacterial homologs were found in all pathways except NHEJ. These prokaryotic and yeast specific genes are not well explored in plant genomes except the bacterial RecA gene. *RecA*, a gene central to general DNA repair and recombination in bacteria, was found to be most conserved bacterial homolog in plant which showed more than 40% amino acid identity in both genomes in comparison to *RecA *of *E. coli*. *Arabidopsis *encodes orthologs of bacterial RecA proteins which are targeted to chloroplasts [14,15] and mitochondria [16] and reported to be associated with DNA repair [16,17]. Complexes of different proteins plays pivotal role in DNA repair processes. We found that plants appeared to retain a partner of bacterial protein complexes while loose other partners. So, in the absence of partner how these bacterial proteins work would be an important question for further investigation.

### Specificities of plant DRR machinery

Functional studies of different genes involved in core DRR pathways in plants, especially *Arabidopsis*, underline the particular and novel behaviour of DRR in plants, amongst the higher eukaryotes. The function of different DRR pathways in plants has been more difficult to demonstrate. For instance, first step in BER is the removal of a base by a glycosylase. 8-oxo-G is the most widespread type of damage repaired by the BER pathway in DNA [18] and plants possess both animal (OGG1) and bacterial (FPG) homolog that remove 8-oxo-G from DNA. The reason why plants have retained these apparently redundant enzymes is not known. Several novel genes, for instance MSH7, BRU1 and RecQsim which are not present in other eukaryotes, are found to be well conserved in plants. Presence of these plant specific genes may lead to a unique DNA repair processes in plants. For instance, MSH7, a bacterial MutS homolog, has been found to be exclusive to plants. It interacts with AtMSH2, as do AtMSH3 and AtMSH6 proteins (other plant homologs of bacterial MutS). Thus it may have a role in maintaining genomic stability which is exclusive to plants [19].

Several mutations which are reported to be lethal in animals have been found to be non-lethal in *Arabidopsis*. For example, homozygous mouse knockouts for ERCC1 died before weaning [20] while atercc1 plants were found to be phenotypically normal in absence of exogenous DNA damaging agents [21]. Same was true for RAD50, MRE11, RAD51, MUS81, BRCA1 and BARD1 also. CRY proteins, members of flavoproteins superfamily, are reported to be ubiquitous in all kingdoms of life. All members of this superfamily possess the characteristics of an N-terminal photolyase homology (PHR) domain. In bacteria, these enzymes mediate photoreactivation but the homologs of CRY protein, CRY1 and CRY2, in insects, animals and plants play a role in blue-light perception and circadian rhythm entrainment instead of DNA repair [22].

**Figure 2:**
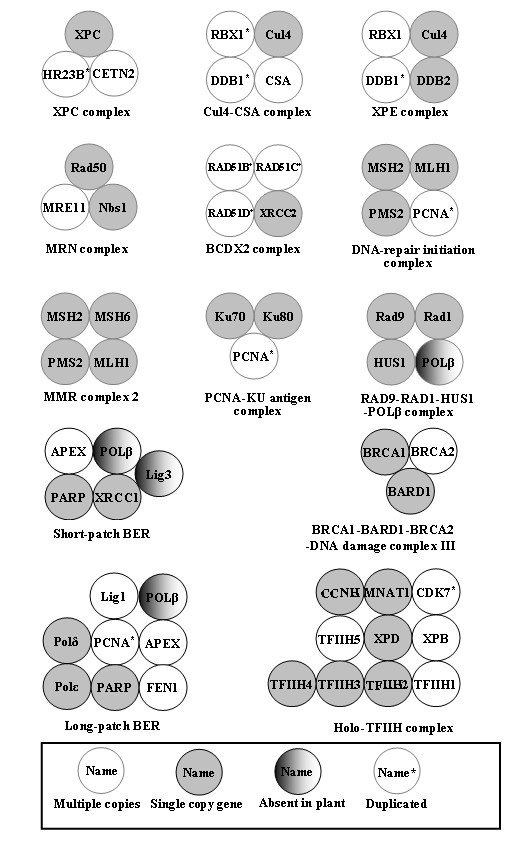
**Conservation and duplication in protein complexes**. Conservation and duplication in known protein complexes in DRR pathways. The way to interpret the figure and special shadings are indicated in the inset.

DNA interstrand cross-links (ICLs) represent lethal DNA damage because they block transcription, replication, and segregation of DNA. Unlike the animals, SNM1A-deficient *Arabidopsis *plants were not found to be hypersensitive to the ICL forming agents like cisplatin and MMC, while displayed a moderate sensitivity to the bleomycin and H_2_O_2 _[23]. In Atsnm1, the frequency of somatic HR (HRF) was also not found to be enhanced as compared with the wild-type plants, suggested the existence of an SNM1-dependent recombinational repair process of oxidatively induced DNA damage in plants [24]. Yeast cells deficient for Ku70 have been shown to possess short-ended telomeres [25], while *Arabidopsis *plants lacking Ku70 or Ku80 found to have longer telomeres than wild-type [26,27]. These results clearly indicated the different mechanisms of telomere maintenance in plants. Beside the above mentioned difference, plants lack many important genes like RAD52, RAD55 and RAD57 which play key role in different DRR pathways in other organisms. Although several complexes that are reported to be involved in DRR other than plants were also well conserved in plant but few members like DNA polymerase β and DNA ligase 3 were absent in plants suggested towards the specificity of plant DRR machinery (Figure 2). Presence of some novel genes and unconventional behaviour of DRR mutant plant lines suggests occurrence of some novel process to cope up with different types of DNA damages in plants.

### Differences in DRR machinery among plants

Despite of strong conservation of DRR proteins, we have observed intriguing difference in DRR genes in plant genomes also. This difference ranges from difference in number of homlogs to the absence or presence of a particular protein (Additional file 1). For example *Arabidopsis *have been found to contain two paralogous copies of xeroderma pigmentosum complementation group B (XPB), a component of the transcription factor IIH (TFIIH), gene. Analysis of the presence of XPB gene in different plant genomes including rice, *Medicago trunculata*, *Carica papaya*, *Vitis venifera*, *Zea mays*, *Glycine max*, sorghum and populus have indicated that beside *Glycine max *none of the analyzed plant genome has two paralogs for XPB genes. Both genes (Joint Genome Institute accession number-Glyma08g01970 and Glyma05g37610) of *Glycine max *were found to be originated from intergenomic duplication from *Arabidopsis*. These results indicated towards genus specific expansion of XPB and worth further investigation. FEN1, a nuclease, show an interesting pattern of evolution. All monocotyledonous genomes we analyzed, rice, *Zea mays*, sorghum and *Brachypodium*, were found to contain two copies of FEN1 and these copies were not the products of intragenomic duplication. On the other hand, all the dicotyledonous genomes that we have studied, Arbidopsis, *Medicago trunculata*, *Vitis venifera*, *Glycine max *and populous, have single copy of FEN1 except *Glycine max*. These copies of FEN1 in *Glycine max *were the product of intragenomic duplication and, unlike monocotyledonous FEN1 genes, acquire same node on phylogenetic tree (our unpublished data). Thus, FEN1 shows lineage specific evolution. Significant difference has also been observed in the duplication level in DRR genes of *Arabidopsis *and rice. Taken together, these results possibly reflecting some differences in the mechanisms and pathways by which damaged DNA are repaired by *Arabidopsis *and rice genomes.

### Functional diversity of DRR genes

**Figure 3:**
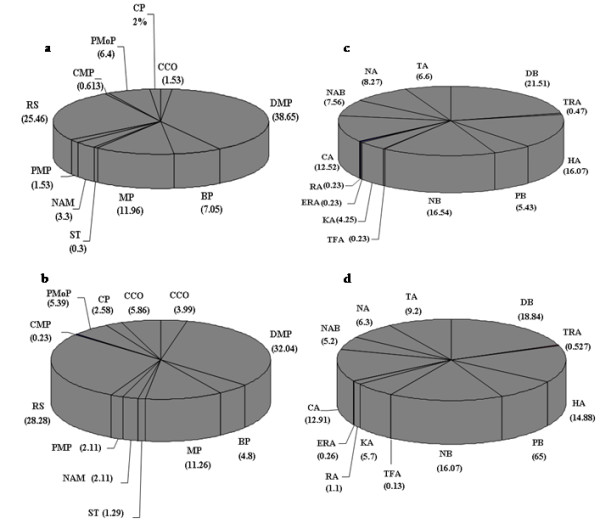
**Functional categorization of rice and *Arabidopsis *DRR genes**: biological process (a, b) and molecular function (c, d) were determined for rice (a, c) and *Arabidopsis *(b, d) DRR genes and percentage of genes included in each category are given. Molecular functions are: DNA binding DB, transcription regulator activity TRA, hydrolase activity HA, protein binding PB, nucleotide binding NB, signal transducer activity TFA, kinase activity KA, receptor activity RA, enzyme regulator activity ERA, catalytic activity CA, nucleic acid binding NAB, nuclease activity NA, transferase activity TA. Biological processes are: cellular component organization CCO, DNA metabolic process DMP, biosynthetic process BP, metabolic process MP, signal transduction ST, nucleobase, nucleoside, nucleotide and nucleic acid metabolic process NAM, protein metabolic process PMP, response to stress RS, carbohydrate metabolic process CMP, protein modification process PMoP, catabolic process CP, cell cycle CCO.

Many DRR proteins display a number of diverse activities unrelated to their DRR processes. Their role in normal plant growth and development is well established [28,29]. We have used DRR genes in plants to investigate functional diversity and complexity of genome maintenance pathways. To gain insight into the functional diversity of predicted rice and *Arabidopsis *DRR, Gene Ontology (GO) annotations (GO Consortium, 2001) were explored. This analysis has revealed that the largest number of DNA replication and repair genes, 21.51% and 18.84% for rice and *Arabidopsis *respectively, fall into the DNA binding molecular functional categories. As expected, relatively good number of genes (38.65% and 32.04% of total DRR genes of rice and *Arabidopsis *respectively) falls in DNA metabolic process biological process category (Figure 3). It was interesting to note that a large fraction, 25.46% of rice and 28.28% of *Arabidopsis *DRR pathway were found to respond against stress. However, a significantly large number of DRR genes fall into cell cycle, protein modification process and biosynthetic process biological process categories compared to total genes analyzed, indicating their crucial role in fundamental cellular processes. Although sixty nine genes of rice were found without any GO annotation but percentage of genes predicted under various molecular function and biological process categories were similar for both rice and *Arabidopsis *probably indicating towards the high conservation of DRR process.

### Gene duplicates and their evolution in DRR genes

**Figure 4:**
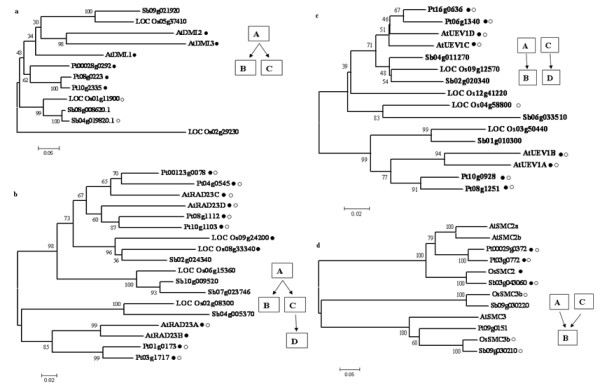
**Phylogenetic analysis of representative duplicated genes family**. Consensus bootstrap Neighbor-Joining phylogenetic tree of representative duplicated genes, DML, RAD23, UEV1, SMC2 and SMC3 gene families. The length of branch lines indicates the extent of divergence according to the scale at the bottom. Name of a gene which shows intrachomosomal segmental duplication is followed by dark circle and which show interchromosomal segmental duplication is followed by white circle. Name of genes which show both phenomena, intra and inter genome segmental duplication, are followed by both dark and white circles. Model of duplication of respective gene families are sketches at right panel (see text for details). (a) Phylogeny of DML. (b) Phylogeny of RAD23. (c) Phylogeny of UEV1 (d) Phylogeny of SMC2 and SMC3.

The importance of gene duplication in supplying raw genetic material to biological evolution has been consensus among researchers. Thus, investigation of gene duplication in the genome not only appropriate for the study of evolutionary processes, but also allow an opening to examine the evolutionary history of multiunit protein complexes that have come up through gene duplication. To counter the adverse conditions, the plant genomes have been shown to follow two mechanisms: one was that there are more copies of the gene families than in animals, and the other was the divergence of genes to fit the pressure of positive selection [30]. Therefore, we have next investigated the duplication in DRR genes in search of evidence on the mechanisms by which plant cells evolve their DRR machinery. Segmental duplication has shown to occur most frequently in plants since most plants are diploidized polyploids and retain numerous duplicated chromosomal blocks in their genomes [31]. Thus in the present study, we considered only segmental duplication and avoided other kinds of duplications. To gain insight into the expansion mechanism of DNA repair genes we investigated their chromosome locations and gene structures also (Additional files 4, 5, 6, 7, and 8). We found that in *Arabidopsis *BER pathway, 5 genes namely Tag, NTH, DML1, HMGB1 and MAGLP are duplicated while none of the BER pathways genes have been duplicated in rice. MAGLP, Tag and DML1 are duplicated multiple times in *Arabidopsis*. MAGLP (locus AT1G19480) duplicated twice into AT3G50880 and AT1G75230. Although these three genes were found to be located in a paralogous cluster in the phylogenetic tree (data not shown), AT1G19480 and AT1G75230 showed the closest paralogous relations in the terminal. Both AT1G19480 and AT1G75230 followed the same intergenic regions. Eight and nine genes in their upstream and downstream respectively were highly conserved suggesting the segmental duplication origin of both loci. Although AT3G50880 faced an intron loss but it retained the functional domain. Among seven predicted candidates for Tag in *Arabidopsis*, four loci were found to be originated from duplication at different time interval. Locus AT1G15970-AT1G80850 (Ks value 0.75) and locus AT1G15970-AT1G75090 were came into origin very recently in comparison to locus AT1G15970-AT3G12710 (Ks value -1). The phylogenetic distribution of different loci also supported the same assumptions. In loci AT1G15970-AT1G80850 and loci AT1G15970- AT1G75090 pairs, AT1G80850 and AT1G75090 showed an intron gain while in loci AT1G15970-AT3G12710 pair, loci AT3G12710 showed an intron loss during the long evolutionary period. Eight genes in upstream and three genes in downstream of loci AT1G15970-AT1G80850 were found to be completely conserved suggesting that this block was also originated by segmental duplication. NTH gene pair (Ks value 0.55) was a result of recent duplication and locus AT2G31450 showed two intron gain events in comparison to locus AT1G05900. HMGB1 gene pair (Ks value -1) was found to be an ancient duplication where locus AT4G35570 showed an intron loss event in comparison to locus AT3G51880. In DML1 gene, both duplication events, loci AT2G36490-AT3G10010 (DML1-DML2) (Ks value 2.69) and loci AT2G36490- AT5G04560 (DML1-DML3) (Ks value 2.69), seemed to occur in very close time span while DML3 and DML2 were sharing same node on phylogenetic tree probably because of old duplication event by which these two non-duplicates come closer than DML1 (Figure 4a).

In the sense of duplication, we found NER as a unique pathway where eight genes, CDK7, PCNA, RPA1, RBX, RAD23, LIG1 and DDB1, were found to be duplicated in *Arabidopsis *while RAD23 and DMC1 were duplicated in rice genome. CDK7, RBX1 and RAD23 paralogs were duplicated many times in course of *Arabidopsis *evolution. DDB1 (Ks value 0.57) and RPA1 (Ks value 0.79) duplicated quite recently than PCNA (Ks value 1.13) but the all three genes pairs retained same gene structure (Additional file 5). LIG1 duplication (Ks value 0.39) was quite newer but locus AT1G49250 showed an intron gain in comparison to locus AT1G08130. CDK7 duplicated three times. Among three gene pairs, locus AT1G18040-AT1G73690 pair appears to duplicated very recently and locus AT1G73690 maintained same gene structure as AT1G18040. On the other hand, other two pairs AT1G18040- AT1G67580 and AT1G18040-AT3G48750, ancient duplicates, showed intron loss and intron gain events during evolution. RBX1 and RAD23 showed relatively complex trend of evolution. Four paralogs of RAD23 are present in *Arabidopsis *which appear to be originated in different duplication events. Gene pair, locus AT1G16190-AT1G79650 (RAD23A-RAD23B) duplicated recently (Ks value 0.6) and retained the similar gene structure. Locus AT3G02540, RAD23C, was involved in two different duplication events with AT5G16090, RAD23A, (Ks value 0.63) and AT5G38470, RAD23 D, (Ks value 1.49) at two different times. It was worth notice that RAD23C - RAD23 D pair, an ancient duplication event, retained the same gene structure but RAD23C - RAD23A differ in their gene structure. Among rice RAD23 paralogs, loci LOC_Os08g33340 and LOC_Os09g24200 were found to be duplicated to each other and maintained the similar gene structure (Figure 4b, Additional file 5).

**Figure 5:**
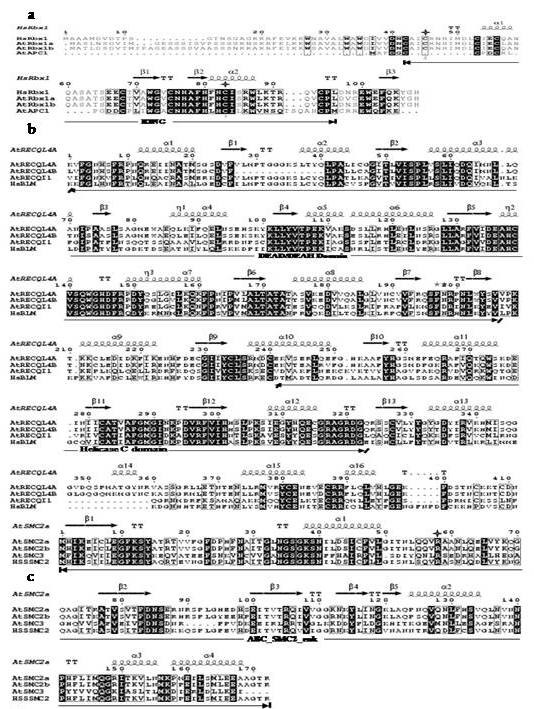
**Multiple sequence alignments of those duplicated *Arabidopsis *genes which show subfunctionalization**. Black shading indicates identical residues in all aligned sequences. Residues within columns show similar chemical properties. Secondary structure elements of respective proteins have been given above alignment. Experimentally proven important residues for the function of particular proteins are marked with stars. Only the well-conserved part of the protein containing important domains is shown for AtRECQL4 and AtSMC2 while full length alignments has been given for AtRBX1 (see Additional file 10 for detail methods). (a) RBX1 (b) AtRECQL4 (c) AtSMC2.

It is also interesting to note that RAD23 was found to be duplicated in all four genomes, *Arabidopsis*, rice, Populus and Sorghum. Populus was unique among four genome as it seems to possess more than four paralogs for RAD23 and all of them are originate by inter and intra-genomic gene duplication events (data not shown here). Two candidate loci, AT3G42830 and AT5G20570, for RBX1 were duplicated relatively recently (Ks value 1.21) but AT5G20570 was itself derived from the duplication of locus AT3G05870 very anciently (Ks value -1) and showed three intron gain events. Although it retained the ancient RBX1 (RING-BOX 1) domain but found to be involved in new functions and pathway probably indicating the subfunctionalization (Figure 5a). Phylogenetic study has supported the idea that loci AT3G42830 and AT5G2057 are relatively closer than locus AT3G05870 (data not shown). Another gene DMC1 was also found to be duplicated in rice and maintain the same gene structure in evolution (Additional file 6).

Beside the above discussed genes, Muts like protein (in MMR), RAD21 (in NHEJ), RecQl4 and RecA (in HR) UBC1, UBE2N and MMS2 (in RAD6 pathway), CHEK1, CHEK2, CLK2A, PR19A/PUB60-1, AXR1, SMC2 and SMC3 (in other conserved DNA damage response genes) were found to be duplicated in *Arabidopsis *at different time during evolution (Additional file 5). Among these genes, RecQl4, SMC2 and MMS2 of *Arabidopsis *deserve special description because of their complex mode of evolution. Two loci, AT1G10930 and AT1G60930, were predicted as putative candidates for RecQl4 in *Arabidopsis*. Both loci appear to be duplicated recently while the locus AT1G10930 was duplicated from locus AT3G05740, candidate gene for RecQI1, very earlier and acquire many introns and different function also (Figure 5b). Functional diversion of these two loci, AT1G10930 and AT1G60930, has been discussed in great detail else where [32]. The similar kind of evolution pattern was also found in SMC2 and SMC3 where both candidate gene for SMC2, AT3G47460 and AT5G62410, were duplicated recently while locus AT5G62410 itself duplicated from locus AT2G27170, SMC3, (Figure 4c and Figure 5c). MMS2 (UEV1) gene family has four members and all of them were duplicated in pair-wise manner to each other. This assumption was also supported by the phylogenetic analysis data where duplicated pair acquires the nearest nodes of its duplicated partners (Figure 4d). In Rice, UBC, SSPR1 and CHEK1 genes were found to be duplicated and surprisingly most of the genes maintained the same gene structure.

### DRR in chloroplast and mitochondria

**Figure 6:**
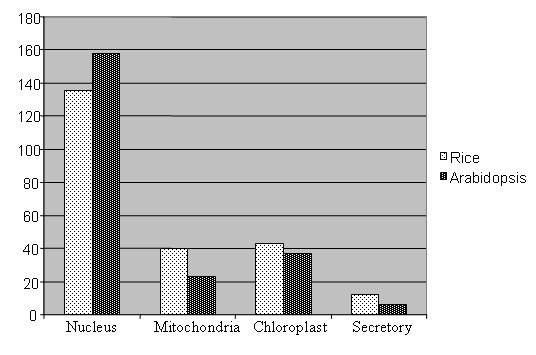
**Sub-cellular distributions of DRR genes**. Distribution of rice and *Arabidopsis *DRR genes in different sub-cellular compartments.

According to the endosymbiotic theory of organelle evolution, plastids and mitochondria are descendants of a prokaryotic photosynthetic organism that established a symbiotic relationship with an early eukaryotic host [33]. Both organelles maintain a separate genome. Organelle genomes have not retained all genes of its ancestor because part of genes may have been lost, some of them functionally replaced by genes in the nucleus, while others have been transmitted to the nucleus [34]. Coordination of gene expression among nucleus, plastids and mitochondria is critical for plant development and survival. Although DRR in animal organelles has been studied up to some extent [35] but the role of organelle DRR in the survival and evolution of plants is less explored. However very recently few researchers have successfully demonstrated the presence of DRR processes in mitochondria and chloroplast [36,37]. Therefore, in the present study we have further predicted the cellular localization of all putative DRR genes in higher plants to gain the insight into organelles DRR. TargetP [38] prediction suggested that 17% and 10% of all DRR genes studied here in *Arabidopsis *were of chloroplast and mitochondrial origin respectively while 19% and 17% of rice genes were chloroplast and mitochondrial origin respectively (Figure 6). These significant similarities in number of organelle's gene probably indicated towards their strong conservation during evolution and needs further detail studies. 24 and 21 genes among *Arabidopsis *and rice DRR genes were prokaryotic specific. Four and eight genes among prokaryotic specific genes were predicted to be non-organeller gene. The presence of prokaryotic specific genes in nuclear genome may be due to the transfer of these genes from organelle's genome to nuclear genome during course of evolution because the migration of genetic information between mitochondria, chloroplasts and nuclei have been fundamental part of evolution and has a ongoing impact on the biology of cells.

## Conclusion

We have utilized the benefits of presence of whole genome sequence of rice and *Arabidopsis *to analyze the uniformity and ubiquity of DRR genes and pathways in higher plant genome. Additionally, extension of many previously reported components have facilitated comparison within plants and between plants and other eukaryotes. Through genome-wide bioinformatic analysis and a comprehensive review of the existing literature, we have described that the core DRR machinery is highly conserved across plant species and displays many features in regular with other eukaryotes and some characteristics that are unique to plants. The absence and the presence of certain genes are discussed and predictions are made considering the particular aspects of the human/*Saccharomyces cerevisiae/E. coli*. The detected similarity and differences broaden present information about what is known for DRR in the plants, and offer a constructive framework for further experimental studies in these organisms. Generalization to other plant species is affirmed by the inclusion of both a monocot and a dicot in this analysis. Finally, extensive comparison of rice and *Arabidopsis *genome in respect to presence and absence of DRR genes may help understanding the basic difference between monocots and dicots. Furthermore, the datasets provided can serve as valuable source for further comparative, evolutionary and functional studies. A detailed understanding of the core DRR machinery in plants will provide researchers with an important tool for understanding what makes plants unique with respect to repair and developmental competence and for investigating how plant genome maintenance strategies differ from the mechanisms employed by animals.

## Methods

### Identification of DRR genes

All the annotated gene and protein sequences of rice chromosomes (TIGR release 6) were downloaded from The Institute for Genomic Research (TIGR) Rice Genome Annotation website [39]. For *Arabidopsis*, all the annotated gene and protein sequences (TAIR release 8) were downloaded from The *Arabidopsis *Information Resource (TAIR) database [40]. The redundant and genes annotated as transposable elements were removed to get a final list of non-redundant genes in rice and *Arabidopsis*. To identify DRR genes we used two different criteria. To select the correct putative orthologue first, we performed global alignments using the FASTA BLAST suit [41] and rejected all alignments with less than 20% identity, unless a portion of the protein showed a very strong similarity. Secondly, a possible conserved motif was searched using the Conserved Domain Database (CDD) [42]. When none of these two approaches produced significant result, the corresponding gene was discarded. Primarily, Potential DRR genes in rice and *Arabidopsis *genome were identified by sequence similarity searches using the DNA repair genes of human, *Saccharomyces cerevisiae *and *E. coli *as seed sequences orthologs and keyword searches. Secondly *Arabidopsis *DRR genes were used to identify the putative DNA repair genes in rice genome. We, next, we searched the primary literature, and when presented, included experimental results that concerned to plant systems to authenticate the annotation. Kyoto Encyclopedia of Genes and Genomes (KEGG) pathway database [43] was used as guide for the gene involve in main repair and recombination pathway while we follow the classification of Wood et al. for the DNA repair genes[44].

### Analysis of DRR genes

The percentage of amino acid identity between a given human/*Saccharomyces cerevisiae/E. coli *gene and its corresponding orthologue in *Arabidopsis *and rice was obtained from the Smith-Waterman alignment between the two sequences. Means were calculated using these values.

Duplicated genes and their Ka, Ks and e-values in DRR proteins of rice, *Arabidopsis*, sorghum and populus were extracted out from the Plant Genome Duplication Database (PGDD) [45]. PGDD is a public database to identify and catalogue plant genes in terms of intra or inter-genome syntenic relationships.

Phylogenetic trees were generated for a group of DNA repair protein homologs. Protein sequences were aligned using the Clustal W multiple sequence alignment program [46]. Only unambiguously aligned positions (excluding poorly conserved and gap regions) were used in phylogenetic analysis, which was performed using the MEGA4 [47]. Neighbor-Joining and Parsimony methods were used for phylogenetic tree searching and inference. The phylogenetic trees were tested by bootstrap analysis with 10000 replications and strict consensus trees were constructed. Similar topologies were found for both algorithms employed, and only Neighbor-Joining being displayed. We used PGDD naming convention for the populus and sorghum dataset in this analysis as the original naming style for predicted models were un-informative. The respective accession numbers used in this analysis and the original naming of predicted genes are given in Additional file 9.

Functional diversity of DRR proteins was carried out according to the GO rules using the gene ontology tools at http://www.agbase.msstate.edu. Only two independent sets of ontologies were used to describe a gene product: (1) the biological process in which the gene product participates and (2) the molecular function that describes the gene product activities.

Additional methods are given in Additional file 10.

## Authors' contributions

SKS and SR designed and performed most of the experiments. SKS, SR and SRC analyzed the result and wrote the initial version of the manuscript. DNSG supervised this study and gave textual advice. All the authors read and approved the final manuscript.

## Supplementary Material

Additional file 1 **DNA repair and recombination related genes in *Arabidopsis *and rice**. Click here for file

Additional file 2 **List of DRR genes of *Arabidopsis *and rice**. Click here for file

Additional file 3 **Chromosome wise distribution of DRR genes in (a) *Arabidopsis *(b) rice. Values in brackets are in percentage**. Click here for file

Additional file 4 **Location of DRR genes on different chromosome of *Arabidopsis *by (a) their function in different pathway and (b) by their TAIR accession number**. Click here for file

Additional file 5 **Gene structure of the intragenomic duplicated DRR gene in *Arabidopsis***. Click here for file

Additional file 6 **Gene structure of the intragenomic duplicated DRR gene in rice**. Click here for file

Additional file 7 **Gene structure of the intergenomic duplicated DRR gene in *Arabidopsis *and rice**. Click here for file

Additional file 8 **Gene name, E-value, Ka value and Ks value of Intra and intergenomic duplication in rice and *Arabidopsis *DRR genes**. Click here for file

Additional file 9 **Accession numbers for Populous and Sorghum**. Click here for file

Additional file 10 **Additional methods**. Click here for file
