# Supplementary material for: DNA repair and recombination in higher plants: insights from comparative genomics of arabidopsis and rice
Source: BMC Genomics. 2010 Jul 21;11:443. doi: 10.1186/1471-2164-11-443 (PMC3091640; doi:10.1186/1471-2164-11-443)
Supplement: Additional file 1 — DNA repair and recombination related genes in Arabidopsis and rice. [file 1471-2164-11-443-S1.DOC]

**Additional file 1**: DNA repair and recombination related genes in Arabidopsis and rice

**Gene Name^3^ Function^2^ Main domain^7^ At^1^ Os^1^**

**Base Excision Repair (BER)**

APE1 Lyase cl00490, cl02640 1 2

APE2 Lyase cl00490 1 1

FPG Glycosylase pfam01149, pfam06831 1 2

MAGLP/AlkA^5^ Endonuclease cd00056 3 2

MBD4 -do- -do- 1 1

MAG/MPG Glycosylase cd00540 1 1

MUTY Endonuclease cd00056, cd03431 1 1

NTH^5^ -do- cd00056 2 1

OGG1 Glycosylase cd00056, pfam07934 1 1

Tag^5^ -do- cl01059 7 6

UNG -do- cl00483 1 1

XRCC1 Transcription cl00038 1 1

coactivator

HMGB1^5^ DNA binding cl00082 1 1

**BER related**

PARP1 Ribosyltransferase cd01437, pfam08063 1 1

PARP2 -do- cd01437 1 1

PARP3 -do- cd01437, pfam08063 1 1

DML1^5, d^ Endonuclease cd00056 3 3

PNKP Phosphoesterase cd01427, pfam00645 1 1

TDP1-do- pfam06087, cl00062 1 1

APTX DNA- binding cd01278, cl00019 1 1

APE1L 1 1

**Nucleotide Excision Repair (NER)**

CCNH Kinase cd00043 1 1

CDK7^5^ -do- cd00180, pfam00069 3 3

CSA Nucleotide binding cl02567 2 2

CSB/ERCC6 DNA binding cd00079, cd00046 1 1

CUL4 - pfam00888 1 1

DDB1^5^ Nucleotide binding COG5161 2 1

DDB2 -do- cl02567 1 1

GTF2H1 Transcription factor cl02772 2 1

-related

GTF2H2 -do- cd01453 1 1

GTF2H3 -do- cl02173 1 1

GTF2H4 -do- cl04289 1 1

GTF2H5/TTDA DNA binding pfam06331 2 2

LIG1^5^ Ligase pfam04675, cl00624 1 1

RAD1/UVH1 Deoxyribonuclease pfam02732 1 1

/ERCC4/XPF

RBX1^5^ Ligase cl09104 2 2

RFC^e^ ATPase cd00009, pfam08542 5 5

RPA1^e, 5^ Nucleic acid binding cd04476, cd04474, 5 3

cd04475, cd04477

RPA2^e^  Protein binding cd04478 2 3

RPA3 2 1

XPD/UVH6 Helicase cl10452 1 1

/ERCC2

UVR7/ERCC1 Endonuclease pfam03834 1 1

XPB/ERCC3 Helicase cd00079, cd00046 2 1

UVR1/UVH3/ Nuclease cd00128 1 1

XPG/ERCC5

XAB2 Binding cd00189 1 2

MNAT 1 Kinase pfam06391 1 1

XPC DNA binding pfam10405, pfam03835 1 1

RAD23^a,5,6^ DNA binding cd01805 4 4

**NER Related**

UvrD-E.coli ATP binding cl09979 1 1

RAD16-S.cerevisiae Helicase cd00079, cd00046 2 1

MMS19 Binding NCD 1 1

Mfd-E.coli Helicase cd00079, cd00046 1 1

CETN2 Calcium ion binding cd00051 2 2

**Homologous Recombination (HR)**

BRCA1 Ligase cd0002, cd00162 1 1

BRCA2 Protein binding cd04494, cd04493 2 1

MRE11A Phosphatase pfam04152 1 2

MUS81 Endonuclease pfam02732 1 1

RAD50 Nuclease cd03240 1 1

RAD51 DNA binding cd01123 1 2

RAD51B Recombinase cd01123 1 1

RAD51C DNA binding cd01123 1 1

RAD51D DNA binding cl09099, PRK09361 1 1

RAD54L Helicase cd00079, cd00046 1 1

NBS1 - cd00060 1 1

BLM/RecQl4^5^  Helicase cd00079, cd00046 2 1

SHFM1/DSS1 - NCD 2 1

XRCC2 - cl09099 1 1

XRCC3 DNA binding cd01123 1 1

TOP3 Topoisomerase COG0550, cd00186 2 2

SSB SS DNA binding cd04496 2 2

EME1 Endonuclease NCD 2 1

**HR related**

DMC1^6^ DNA binding cd01123 1 2

MND1 - COG5124 1 1

RecA-E.coli^5^ ATPase cd00983 3 4

RecG-E.coli Helicase COG1200 1 1

BARD1 Transcription cd00027 1 2

Coactivator

MIM ATP binding COG1196, cd03276 1 - , pfam10359

**Mismatch Excision Repair (MMR)**

MLH1 Protein binding cd03483 1 1

MLH3 ATP binding pfam08676, cl02783 1 1

MSH1 DNA binding cd03243 1 1

MSH2 -do- cd03285, cl04977 1 1

MSH3 -do- cd03287 1 1

MSH4 -do- cl09099, cl04977 1 1

MSH5 -do- cd03281 1 1

MSH6 -do- pfam01624, cl09099 1 1

MSH7 -do- pfam01624 1 1

PMS1   ATP binding cd03484 1 1

Muts like DNA binding cd03280 2 2

Protein^5^

**Non-Homologous End-Joining (NHEJ)**

Ku70 DNA binding, cd00788 1 1

Ku80 -do- cd00873 1 1

Lig4 Ligase cd00027, cl00624 1 1

pfam04679

PRKDC Kinase cd05169 1 1

XRCC4 Protein binding NCD 1 1

**NHEJ related**

RAD21 - pfam04824, pfam04825 3 3

**Editing and processing nucleases**

FLJ35220 Endonuclease cl00653 1 1

HEX1/EXO1 Nuclease cd00128 2 1

SPO11b Topoisomerase cd00223 3 3

FEN1 Nuclease cd00128, PRK03980 1 2

GEN^c^  -do- cd00128 2 2

**Modulation of nucleotide pools**

DUT1 dUTP diphosphatase cl00493 1 1

RNR1 Ribonucleoside cd01049 1 2

(Large subunit) -diphosphate reductase

RNR2 -do- cd01049 3 2

(Large subunit)

NUDX1 pyrophosphohydrolase cd04678 1 1

**DNA polymerases (catalytic subunits)**

POLD1 -do- COG0417, cd05777, cd05533, 1 1

POLD2 -do- COG1311, pfam04042 1 1

POLD3 -do- pfam09507 1 1

POLD4 -do- pfam04081 1 2

POLE1 -do- cd05779, cd05535, pfam031041 1

POLE2 -do- -do- 1 1

POLE3 - pfam04042 1 2

POLH -do- cd01702 1 1

POLL -do- cl10526 1 1

REV1 Polymerase cd00027, cd01701 1 1

REV3 -do- COG0417, cd05778, cd05534 1 2

REV7 DNA binding pfam02301 1 1

PCNA^5^ -do- cd00577 1 2

POLK - cd03586 1 1

**Rad6 pathway**

MMS2^5^ Protein binding cl00154 4 4

UBC^5,6^ Ligase cd00195 3 3

UBE2N^5^ -do- cd00195 2 1

**Direct reversal of damage**

CRY1^4^ Blue light COG0415, pfam00875 1 2

Photoreceptor , pfam03441

CRY2^4^ -do- -do- 1 1

CRY3^4^ DNA binding -do- 1 1

PHR1 DNA photolyase -do- 1 1

PHR2 -do- -do- 1 1

AlkB - COG3145 1 1

ABH3/ AlkB homolog 3 - -do- 1 1

UVR3 Photolyase pfam00875, pfam03441 1 1

**Genes defective in diseases associated with sensitivity to DNA damaging agents**

WRN Exonuclease cd06141 1 1

ATM Kinase pfam02259, cl02554 1 1

**Other conserved DNA damage response genes**

RECQI1 Helicase cd00046, cd00079 1 1

RECQL2 -do- -do- 1 1

ReCQl3 -do-` -do- 1 1

RECQSIM -do- -do- 1 1

SNM1 - COG1236, pfam07522 2 2

SNM1B - -do- 1 1

ReCQ886 - cd00079, cd00046 - 1

ReCQL5 Helicase cd00046, cd00079 1 1

ATR Kinase pfam02259, cd00892 1 1

RAD9 - pfam04139, cl09515 1 1

RAD17 - pfam03215 1 1

CHEK1^5,6^ Kinase cd00180 1 1

CHEK2^5^ -do- cd00051, cd00180 1 1

CLK2A^5^ -do- cd00180 1 1

CLK2B -do- -do- 1 1

CLK2C -do- -do- 1 1

RAD1 DNA binding cd00577 1 1

HUS1 - pfam04005 1 1

AXR1^5^ Small protein cd01493 1 1

activating enzyme

SRPP1^6^ Transcription cd01390 1 2

factor activity

PR19A/PUB60-1^5^ Nucleotide binding cd00200 1 1

PR19B/PUB60-2 -do- -do- 1 1

SM3L2*/RAD5a* DNA binding cd00046, cd00079 1 1

SM3L3*/RAD5a* -do- -do- 1 1

DRT100 Nucleotide binding COG4886, pfam08263 1 1

DRT102 - cl00485, cl09118 1 1

DRT111 - cl02586, cl02611 1 1

DRT101 Phosphotransferases COG1109, cd03086 1 1

DET1 - pfam09737 1 1

COP1 Protein binding cd00162, cd00200 1 1

BRU1 protein binding cd00189, cl02423 1 2

SMC1 ATP binding cd03275 1 1

SMC2^5^ Transporter activity cd03273 2 1

SMC3^5^ ATP binding cd03272 1 1

SMC4 ATP binding cd03274 1 1

SMC5 ATP binding cd03277 1 1

SMC6 ATP binding cd03276 1 1

PRD1 Protein binding NCD 1 1

^1^ At and Os are *Arabidopsis thaliana* and *Oryza sativa* respectively. NCD- No Conserved Domain

^2^ Functions are indicated when known either experimentally or putatively. Functions were searched out by using the Arabidopsis gene only.

^3^Genes which are not included in main pathway by KEGG pathway database are categorized as ‘related’ genes of respective pathway.e.g. BER related.

^4^These genes are reported to be not involved in repair processes in plants.

^5^ These genes are duplicated in Arabidopsis.

^6^ These genes are duplicated in rice.

^7^CDD accession number

^a^RAd23A, RAd23B, RAd23C, RAd23D ^b^SPO11-1, SPO11-2, SPO11-3

^c^ GEN1,GEN2

^d^ DML1,DML2,DML3

^e^ Include all subunits.
