# Supplementary material for: DNA repair and recombination in higher plants: insights from comparative genomics of arabidopsis and rice
Source: BMC Genomics. 2010 Jul 21;11:443. doi: 10.1186/1471-2164-11-443 (PMC3091640; doi:10.1186/1471-2164-11-443)
Supplement: Additional file 2 — List of DRR genes of Arabidopsis and rice. [file 1471-2164-11-443-S2.DOC]

| **Gene Name**  Additional file 1: List of DNA repair and recombination genes of Arabidopsis and rice. | **AGI Accession number** | **TIGR Accession number** | **e-value** | **Percentage amino acid identity** | **References** |
| --- | --- | --- | --- | --- | --- |
| **Base excision repair (BER)** |  |  |  |  |  |
| *APE1* | AT2G41460 | LOC_Os01G58680 | e-143 | 252/460 (54%) | 2 |
|  |  | LOC_Os01G58690 | e-113 | 187/293 (63%) |  |
| *APE2* | AT4G36050 | LOC_Os09g36530 | e-131 | 263/611 (43%) | 2 |
| *FPG* | AT1G52500 | LOC_Os06g43610 | e-110 | 182/258 (70%) | 3,4,5,180 |
|  |  | LOC_Os08g21330 | e-100 | 168/254 (66%) |  |
| *MAGLP/AlkA* | AT1G75230 | LOC_Os05G50290 | 7.00E-78 | 139/216 (64%) |  |
|  |  | LOC_Os05G49250 | 5.00E-53 | 110/211 (52%) |  |
|  | AT1G19480 | LOC_Os05G50290 | 1.00E-83 | 146/215 (67%) |  |
|  |  | LOC_Os05G49250 | 5.00E-57 | 118/233 (50%) |  |
|  | AT3G50880 | LOC_Os05G50290 | 2.00E-74 | 137/227 (60%) |  |
|  |  | LOC_Os05G49250 | 2.00E-63 | 127/246 (51%) |  |
| *MBD4* | AT3G07930 | LOC_Os09g01290 | 4.00E-10 | 38/81 (46%) |  |
| *MPG/MAG* | AT3G12040 | LOC_Os02G53430 | 3.00E-87 | 151/195 (77%) | 8 |
| *MUTY* | AT4G12740 | LOC_Os12G10850 | e-102 | 204/436 (46%) |  |
| *NTH* | AT1G05900 | LOC_Os11G16580 | 2.00E-93 | 170/322 (52%) | 7 |
|  | AT2G31450 | LOC_Os11G16580 | 9.00E-87 | 145/276 (52%) |  |
| *OGG1* | AT1G21710 | LOC_Os02G34750 | 2.00E-98 | 182/312 (58%) | 4-6,18 |
| *Tag* | AT1G15970 | LOC_Os06g44050 | 8.00E-62 | 108/213 (50%) |  |
|  | AT1G75090 | LOC_Os03G10220 | 1.00E-73 | 121/208 (58%) |  |
|  | AT1G80850 | LOC_Os09g25290 | 1.00E-61 | 101/188 (53%) |  |
|  | AT3G12710 | LOC_Os01G58550 | 5.00E-79 | 140/275 (50%) |  |
|  | AT5G57970 | LOC_Os04G42290 | 4.00E-87 | 141/187 (75%) |  |
|  | AT5G44680 | None |  |  |  |
|  | AT1G13635 | LOC_Os08g38170 | 1.00E-76 | 124/206 (60%) |  |
| *UNG* | AT3G18630 | LOC_Os04G57730 | 2.00E-99 | 173/266 (65%) | 206 |
| *XRCC1* | AT1G80420 | LOC_Os06g05190 | 4.00E-90 | 176/360 (48%) | 17 |
| *HMGB1* | AT3G51880 | LOC_Os09g37910 | 5.00E-13 | 38/86 (44%) | 15-16 |
| **Other BER factors** |  |  |  |  |  |
| *PARP1* | AT2G31320 | LOC_Os07g23110 | 0 | 589/1002 (58%) | 9,10 |
| *PARP2* | AT4G02390 | LOC_Os01G24940 | 0 | 380/677 (56%) |  |
| *PARP3* | AT5G22470 | LOC_Os02G32860 | 0 | 456/830 (54%) |  |
| *DML1* | AT2G36490 | LOC_Os01G11900 | 0 | 340/539 (63%) | 11-14 |
|  |  | LOC_Os05G37410 | e-180 | 311/543 (57%) |  |
|  |  | LOC_Os02G29230 | e-113 | 223/451 (49%) |  |
| *DML2* | AT3G10010 | LOC_Os01G11900 | e-129 | 246/526 (46%) |  |
|  |  | LOC_Os05G37410 | e-110 | 226/529 (42%) |  |
|  |  | LOC_Os02G29230 | 3.00E-78 | 182/472 (38%) |  |
| *DML3* | AT4G34060 | LOC_Os01G11900 | e-142 | 289/662 (43%) |  |
|  |  | LOC_Os05G37410 | e-123 | 246/539 (45%) |  |
| *PNKP* | AT3G14890 | LOC_Os01G53560 | e-119 | 234/509 (45%) |  |
| *APTX* | AT5G01310 | LOC_Os03G18210 | 0 | 402/737 (54%) |  |
| *TDP1* | AT5G15170 | LOC_Os07g34598 | 0 | 346/644 (53%) |  |
| *APE1L* | AT3G48425 | LOC_Os12g18200 | e-149 | 249/370 (67%) | 1, 2 |
| **Nucleotide excision repair (NER)** |  |  |  |  |  |
| CCNH | AT5G27620 | LOC_Os03G52750 | e-103 | 178/305 (58%) |  |
| CDK7 | AT1G73690 | LOC_Os05G32600 | e-160 | 283/407 (69%) | 19 |
|  |  | LOC_Os03G02680 | 1.00E-78 | 140/286 (48%) |  |
|  |  |  |  |  |  |
|  |  | LOC_Os03G01850 | 1.00E-77 | 139/282 (49%) |  |
|  | AT1G66750 | LOC_Os05G32600 | e-151 | 247/344 (71%) |  |
|  |  | LOC_Os03G02680 | 4.00E-80 | 147/287 (51%) |  |
|  |  | LOC_Os03G01850 | 3.00E-79 | 147/278 (52%) |  |
|  |  |  |  |  |  |
|  | AT1G18040 | LOC_Os05G32600 | e-165 | 285/405 (70%) |  |
|  |  | LOC_Os03G02680 | 4.00E-79 | 140/286 (48%) |  |
|  |  | LOC_Os03G01850 | 4.00E-78 | 139/282 (49%) |  |
| CSA | AT1G19750 | LOC_Os02G20430 | e-119 | 220/456 (48%) |  |
|  |  | LOC_Os01G63900 | 3.00E-50 | 91/187 (48%) |  |
|  | AT1G27840 | LOC_Os02G20430 | 2.00E-88 | *154/260 (59%)* |  |
|  |  | LOC_Os01G63900 | 8.00E-53 | *87/143 (60%)* |  |
| CSB/ERCC6 | AT2G18760 | LOC_Os05G05230 | 2.00E-74 | 174/511 (34%) | 20 |
| CUL4 | AT5G46210 | LOC_Os03G57290 | 0 | 569/736 (77%) | 22,24-25,201 |
| DDB1 | AT4G05420 | LOC_Os05G51480 | 0 | 901/1090 (82%) | 21-25,201 |
|  |  | LOC_Os01G40320 | 6.00E-26 | 61/127 (48%) |  |
|  | AT4G21100 | LOC_Os05G51480 | 0 | 867/1090 (79%) |  |
|  |  | LOC_Os01G40320 | 2.00E-25 | 47/92 (51%) |  |
| DDB2 | AT5G58760 | LOC_Os01G04870 | 0 | 296/465 (63%) | 23,24,26,27 |
| GTF2H1 | AT1G55750 | LOC_Os08g25060 | e-168 | 296/605 (48%) |  |
|  | AT3G61420 | LOC_Os08g25060 | e-140 | 264/572 (46%) |  |
| GTF2H2 | AT1G05055 | LOC_Os04G42990 | e-156 | 263/385 (68%) | 40 |
| GTF2H3 | AT1G18340 | LOC_Os02G03340 | 3.00E-86 | 164/279 (58%) |  |
| GTF2H4 | AT4G17020 | LOC_Os04G58350 | 0 | 313/447 (70%) |  |
| GTF2H5 | AT1G12400 | LOC_Os07g38600 | 6.00E-32 | 54/70 (77%) |  |
|  | AT1G62886 | LOC_Os05G10980 | 7.00E-24 | 45/70 (64%) |  |
| LIG1 | AT1G08130 | LOC_Os10g34750 | 0 | 449/627 (71%) | 29 |
|  | AT1G49250 | LOC_Os10g34750 | 0 | 364/589 (61%) |  |
| RAD1/UVH1/ERCC4/XPF | AT5G41150 | LOC_Os03G01100 | 0 | 511/927 (55%) | 30-33 |
| RBX1 | AT3G42830 | LOC_Os02G47870 | 2.00E-48 | 83/89 (93%) | 34 |
|  |  | LOC_Os01G01700 | 3.00E-48 | 84/89 (94%) |  |
|  | AT5G20570 | LOC_Os02G47870 | 2.00E-50 | 86/88 (97%) |  |
|  |  | LOC_Os01G01700 | 3.00E-50 | 87/88 (98%) |  |
| RFC2 | AT1G21690 | LOC_Os12G07720 | e-167 | 277/337 (82%) | 38 |
| RFC4 | AT1G63160 | LOC_Os04G48060 | e-175 | 283/331 (85%) |  |
| RFC3 | AT1G77470 | LOC_Os02G53500 | e-151 | 254/342 (74%) |  |
| RFC1 | AT5G22010 | LOC_Os11G36390 | 0 | 459/749 (61%) |  |
| RFC5 | AT5G27740 | LOC_Os03g57870 | e-164 | 261/354 (73%) |  |
| RPA1 | AT2G06510 | LOC_Os02G53680 | 0 | 389/668 (58%) | 37,126,183,184 |
|  |  | LOC_Os05G02040 | e-144 | 242/472 (51%) |  |
|  |  | LOC_Os03G11540 | 1.00E-90 | 207/647 (31%) |  |
|  |  | LOC_Os05G02030 | 9.00E-28 | 51/105 (48%) |  |
|  | AT4G19130 | LOC_Os02G53680 | e-166 | 301/674 (44%) |  |
|  |  | LOC_Os05G02040 | e-155 | 266/547 (48%) |  |
|  |  | LOC_Os03G11540 | 7.00E-71 | 152/467 (32%) |  |
|  |  | LOC_Os05G02030 | 2.00E-29 | 54/105 (51%) |  |
|  | AT5G08020 | LOC_Os03G11540 | 0 | 373/622 (59%) |  |
|  |  | LOC_Os02G53680 | 2.00E-75 | 189/663 (28%) |  |
|  |  | LOC_Os05G02040 | 8.00E-70 | 152/450 (33%) |  |
|  |  | LOC_Os05G02030 | 1.00E-19 | 43/96 (44%) |  |
|  | AT5G45400 | LOC_Os05G02040 | 0 | 353/854 (41%) |  |
|  |  | LOC_Os02G53680 | e-153 | 264/531 (49%) |  |
|  |  | LOC_Os03G11540 | 5.00E-76 | 157/466 (33%) |  |
|  |  | LOC_Os05G02030 | 4.00E-30 | 54/105 (51%) |  |
|  | AT5G61000 | LOC_Os03G11540 | 0 | 366/624 (58%) |  |
|  |  | LOC_Os02G53680 | 3.00E-77 | 186/642 (28%) |  |
|  |  | LOC_Os05G02040 | 1.00E-69 | 147/451 (32%) |  |
|  |  | LOC_Os05G02030 | 5.00E-19 | 41/96 (42%) |  |
| RPA2 | AT2G24490 | LOC_Os02G58220 | 7.00E-47 | 95/252 (37%) | 35,36 |
|  |  | LOC_Os02G42230 | 2.00E-44 | 88/245 (35%) |  |
|  |  | LOC_Os06g47830 | 2e-022 | 58/149 (38%) |  |
|  | AT3G02920 | LOC_Os02G58220 | 4.00E-45 | 102/271 (37%) |  |
|  |  | LOC_Os02G42230 | 7.00E-44 | 97/268 (36%) |  |
|  |  | LOC_Os06g47830 | 3e-019 | 42/103 (40%) |  |
| RPA3 | AT3G52630 | LOC_Os01g14980 | 6e-029 | 56/107 (52%) |  |
|  | AT4G18590 | LOC_Os01g14980 | 6e-030 | 55/106 (51%) |  |
| XPD/UVH6/ERCC2 | AT1G03190 | LOC_Os05G05260 | 0 | 587/761 (77%) | 39,40 |
| UVR7/ERCC1 | AT3G05210 | LOC_Os10g37490 | e-104 | 161/201 (80%) | 41-43 |
| XPB/ERCC3 | AT5G41370 | LOC_Os01G49680 | 0 | 582/719 (80%) | 44-46 |
|  | AT5G41360 | LOC_Os01G49680 | 0 | 585/715 (81%) |  |
| UVR1/UVH3/XPG/ERCC5 | AT3G28030 | LOC_Os03G10780 | e-124 | 289/686 (42%) | 47 |
| XAB2 | AT5G28740 | LOC_Os07g44970 | 0 | 587/920 (63%) |  |
|  |  | LOC_Os06g12910 | 0 | 397/794 (50%) |  |
| MNAT1 | AT4G30820 | LOC_Os11G28350 | 2.00E-42 | 86/183 (46%) |  |
| XPC | AT5G16630 | LOC_Os08g33082 | e-170 | 332/788 (42%) | 49 |
| RAD23A | AT1G79650 | LOC_Os02G08300 | e-114 | 222/374 (59%) | 48,176 |
| RAD23B | AT1G16190 | LOC_Os09g24200 | 6.00E-93 | 186/395 (47%) |  |
| RAD23C | AT3G02540 | LOC_Os06g15360 | 6.00E-86 | 164/267 (61%) |  |
| RAD23D | AT5G38470 | LOC_Os09g24200 | 3.00E-99 | 196/392 (50%) |  |
| **NER related** |  |  |  |  |  |
| UvrD - *E. coli* | AT4G25120 | LOC_Os07g30980 | 0 | 533/913 (58%) |  |
| RAD16 - *S. cerevisiae* | AT1G02670 | LOC_Os07g32730 | e-100 | 188/337 (55%) |  |
|  | AT1G05120 | LOC_Os07g32730 | e-145 | 248/369 (67%) |  |
| MMS19 | AT5G48120 | LOC_Os07g08050 | e-107 | 241/661 (36%) |  |
| Mfd - *E. coli* | AT3G02060 | LOC_Os11G32880 | 0 | 462/777 (59%) |  |
| CETN2 | AT3G50360 | LOC_Os07g42660 | 4.00E-71 | 128/167 (76%) | 49,50 |
|  |  | LOC_Os10g25010 | 4.00E-59 | 109/159 (68%) |  |
|  | AT4G37010 | LOC_Os07g42660 | 3.00E-58 | 104/168 (61%) |  |
|  |  | LOC_Os10g25010 | 1.00E-53 | 99/148 (66%) |  |
| **Homologous Recombination (HR)** |  |  |  |  |  |
| BRCA1 | AT4G21070 | LOC_Os05G43610 | e-108 | 197/403 (48%) | 51 |
| BRCA2 | AT5G01630 | LOC_Os01G07110 | e-160 | 413/1185 (34%) | 52,53 |
|  | AT4G00020 | LOC_Os01G07110 | e-157 | 401/1165 (34%) |  |
| MRE11A | AT5G54260 | LOC_Os04G54340 | 0 | 449/599 (74%) | 54-56,179 |
|  |  | LOC_Os08g08030 | 0 | 302/525 (57%) |  |
| MUS81 | AT4G30870 | LOC_Os01G71960 | e-155 | 311/673 (46%) | 57-59,90 |
| RAD50 | AT2G31970 | LOC_Os02G29464 | 0 | 786/1316 (59%) | 60-64 |
| RAD51A | AT5G20850 | LOC_Os11G40150 | e-176 | 292/321 (90%) | 53,65-74,174,175 |
|  |  | LOC_Os12G31370 | e-173 | 288/320 (90%) |  |
| RAD51B | AT2G28560 | LOC_Os05G03050 | e-114 | 207/360 (57%) |  |
| RAD51C | AT2G45280 | LOC_Os01G39630 | e-123 | 213/349 (61%) |  |
| RAD51D | AT1G07745 | LOC_Os09g01680 | 3.00E-60 | 116/253 (45%) |  |
| RAD54L | AT3G19210 | LOC_Os02G52510 | 0 | 506/800 (63%) | 75,76 |
| NBS1 | AT3G02680 | LOC_Os10g34580 | 2e-081 | 194/572 (33%) | 77,78 |
| BLM/RecQl4 | AT1G10930 | LOC_Os04G35420 | e-119 | 234/464 (50%) | 57, 79 |
|  | AT1G60930 | LOC_Os04G35420 | e-104 | 192/336 (57%) |  |
| SHFM1/DSS1 | AT5G45010 | LOC_Os01G16640 | 8.8 | 16/44 (36%) | 53 |
|  | AT1G64750 | LOC_Os01G16640 | 0.82 | 18/44 (40%) |  |
| XRCC2 | AT5G64520 | LOC_Os01G64990 | 2.00E-31 | 72/151 (47%) |  |
| XRCC3 | AT5G57450 | LOC_Os02G35450 | 7.00E-45 | 118/300 (39%) | 86 |
| TOP3 | AT5G63920 | LOC_Os03G06900 | 0 | 521/771 (67%) |  |
|  | AT2G32000 | LOC_Os09g32450 | 0 | 600/863 (69%) |  |
| SSB | AT4G11060 | LOC_Os05g43440 | 1e-071 | 136/208 (65%) | 82 |
|  | AT3G18580 | LOC_Os01g45530 | 1e-057 | 101/153 (66%) |  |
| EME1 | AT2G21800 | LOC_Os04g55500 | 6e-077 | 140/298 (46%) | 59 |
|  | AT2G22140 | LOC_Os04g55500 | 4e-074 | 140/294 (47%) |  |
| **HR-related** |  |  |  |  |  |
| DMC | AT3G22880 | LOC_Os11G04954 | e-164 | 282/344 (81%) | 53,72,80,187,199,203 |
|  |  | LOC_Os12G04980 | e-161 | 279/344 (81%) |  |
| MND1 | AT4G29170 | LOC_Os09g10850 | 4.00E-90 | 154/207 (74%) | 87-89 |
| RecA – *E. coli* | AT2G19490 | LOC_Os01g67510 | e-134 | 233/356 (65%) | 83-85 |
|  |  | LOC_Os11g19720 | e-128 | 222/358 (62%) |  |
|  |  | LOC_Os03g43850 | 4.00E-72 | 148/335 (44%) |  |
|  |  | LOC_Os01g71690 | 2.00E-70 | 144/327 (44%) |  |
|  | AT1G79050 | LOC_Os03g43850 | e-178 | 300/345 (86%) |  |
|  |  | LOC_Os01g67510 | 4.00E-73 | 159/327 (48%) |  |
|  |  | LOC_Os11g19720 | 6.00E-67 | 151/358 (42%) |  |
|  |  | LOC_Os01g71690 | 3.00E-47 | 133/358 (37%) |  |
|  | AT3G10140 | LOC_Os01g71690 | 6.00E-95 | 185/391 (47%) |  |
|  |  | LOC_Os11g19720 | 1.00E-83 | 154/330 (46%) |  |
|  |  | LOC_Os01g67510 | 4.00E-79 | 149/318 (46%) |  |
|  |  | LOC_Os03g43850 | 5.00E-48 | 122/335 (36%) |  |
|  | AT3G32920 | LOC_Os01g67510 | 6.00E-69 | 137/217 (63%) |  |
|  |  | LOC_Os11g19720 | 2.00E-63 | 131/217 (60%) |  |
|  |  | LOC_Os01g71690 | 3.00E-38 | 92/206 (44%) |  |
|  |  | LOC_Os03g43850 | 2.00E-37 | 92/205 (44%) |  |
| RecG – *E. coli* | AT2G01440 | LOC_Os02G48100 | e-155 | 265/410 (64%) |  |
| BARD1 | AT1G04020 | LOC_Os05G40810 | 8e-078 | 156/386 (40%) | 81 |
|  |  | LOC_Os04G43300 | 3e-092 | 170/384 (44%) |  |
| MIM | AT5G61460 | none |  |  |  |
| **Mismatch excision repair (MMR)** |  |  |  |  |  |
| *MLH1* | AT4G09140 | LOC_Os01G72880 | 0 | 67/726 (64%) | 93,94 |
| *MLH3* | AT4G35520 | LOC_Os09g37930 | 3.00E-71 | 179/435 (41%) | 96 |
| *MSH1* | AT3G24320 | LOC_Os04G42784 | 0 | 677/1093 (61%) | 177 |
| *MSH2* | AT3G18524 | LOC_Os05G19270 | 0 | 600/943 (63%) | 95,97,98,99,100,101,103,177 |
| *MSH3* | AT4G25540 | LOC_Os04G58630 | 0 | 567/1037 (54%) | 99,101 |
| *MSH4* | AT4G17380 | LOC_Os07g30240 | e-164 | 283/439 (64%) | 102 |
| *MSH5* | AT3G20475 | LOC_Os05G41880 | e-115 | 214/435 (49%) | 102,104 |
| *MSH6* | AT4G02070 | LOC_Os09g24220 | 0 | 83/1009 (57%) | 99,101,103 |
| *MSH7* | AT3G24495 | LOC_Os01G08540 |  | 555/990 (56%) | 99,101,103,178 |
| *PMS1* | AT4G02460 | LOC_Os02G37920 | 0 | 442/945 (46%) | 105,106 |
| *Muts like*  *protein* | AT5G54090 | LOC_Os10g36530 | e-115 | 286/566 (50%) |  |
|  | AT1G65070 | LOC_Os04g58410 | e-173 | 339/785 (43%) |  |
| **Non-homologous end-joining (NHEJ)** |  |  |  |  |  |
| *KU70* | AT1G16970 | LOC_Os07g08729 | 0 | 373/617 (60%) | 107-109, 112,115 |
| *KU80* | AT1G48050 | LOC_Os03G63920 | 0 | 372/691 (53%) | 107,108,110-115 |
| *LIG4* | AT5G57160 | LOC_Os04G51700 | 0 | 431/697 (61%) | 114,116-118 |
| *PRKDC* | AT1G50030 | LOC_Os05G14550 | 0 | 1018/1357 (75%) |  |
| *XRCC4* | AT3G23100 | LOC_Os03G53000 | 2.00E-62 | 103/185 (55%) |  |
| **NHEJ related** |  |  |  |  |  |
| *ATRAD21.1* | AT5G40840 | LOC_Os01g67250 | 2e-027 | 71/173 (41%) | 119-120 |
|  |  | LOC_Os08g16610 | 3e-021 | 53/128 (41%) |  |
|  |  | LOC_Os04g41110 | 3e-013 | 68/238 (28%) |  |
| *ATRAD21.2* | AT3G59550 | LOC_Os08g16610 | 2e-052 | 212/726 (29%) |  |
|  |  | LOC_Os01g67250 | 1e-032 | 66/144 (45%) |  |
|  |  | LOC_Os04g41110 | 3e-007 | 28/76 (36%) |  |
| *ATRAD21.3* | AT5G16270 | LOC_Os01g67250 | 2e-087 | 154/185 (83%) |  |
|  |  | LOC_Os08g16610 | 5e-041 | 82/144 (56%) |  |
|  |  | LOC_Os04g41110 | 6e-009 | 30/80 (37%) |  |
| **Editing and processing nucleases** |  |  |  |  |  |
| *FLJ35220* | AT4G31150 | LOC_Os06g45330 | 2.00E-52 | 99/156 (63%) |  |
| *HEX1/EXO1* | AT1G29630 | LOC_Os01G56940 | 2.00E-91 | 161/276 (58%) | 126 |
|  | AT1G18090 | LOC_Os01G56940 | 5e-080 | 151/347 (43%) |  |
| *SPO11-1* | AT3G13170 | LOC_Os03G54091 | e-106 | 185/353 (52%) | 123-125 |
| *SPO11-2* | AT1G63990 | LOC_Os08g06050 | e-136 | 237/385 (61%) |  |
| *SPO11-3* | AT5G02820 | LOC_Os03G17610 | e-171 | 275/355 (77%) |  |
| *FEN1* | AT5G26680 | LOC_Os05G46270 | e-171 | 288/341 (84%) | 121,134,185 |
|  |  | LOC_Os03G61820 | e-139 | 236/351 (67%) |  |
| *GEN1* | AT1G01880 | LOC_Os09g35000 | e-163 | 306/639 (47%) | 122 |
| *GEN2* | AT3G48900 | LOC_Os08g01130 | e-135 | 230/426 (53%) |  |
| **Modulation of nucleotide pools** |  |  |  |  |  |
| *DUT1* | AT3G46940 | LOC_Os03G46640 | 3.00E-49 | 98/137 (71%) |  |
| *RNR1* | AT2G21790 | LOC_Os06g07210 | 0 | 698/816 (85%) | 127,188-193 |
|  |  | LOC_Os02g56100 | 0 | 681/816 (83%) |  |
| *TSO2* | AT3G27060 | LOC_Os06g14620 | e-148 | 258/340 (75%) |  |
|  |  | LOC_Os06g03720 | e-147 | 254/327 (77%) |  |
| *RNR2a* | AT3G23580 | LOC_Os06g03720 | e-137 | 231/329 (70%) |  |
|  |  | LOC_Os06g14620 | e-137 | 231/326 (70%) |  |
| *RNR2b* | AT5G40942 | LOC_Os06g03720 | e-148 | 248/328 (75%) |  |
|  |  | LOC_Os06g14620 | e-148 | 250/341 (73%) |  |
| *NUDX1* | AT1G68760 | LOC_Os09g38040 | 7e-005 | 22/59 (37%) | 128 |
| **DNA polymerases (catalytic subunits)** |  |  |  |  |  |
| *POLD 1* | AT5G63960 | LOC_Os11G08330 | 0 | 308/429 (71%) | 136 |
| *POLD* 2 | AT2G42120 | LOC_Os03G03650 | 0 | 180/515 (34%) |  |
| *POLD* 3 | AT1G78650 | LOC_Os01G10690 | 5.00E-62 | 49/126 (38%) |  |
| *POLD* 4 | AT1G09815 | LOC_Os09g34850 | 5.00E-21 | 54/145 (37%) |  |
|  |  | LOC_Os08g43080 | 6.00E-14 | 1336/2247 (59%) |  |
| *POLE* | AT2G27120 | LOC_Os02G30800 | 0 | 1332/2337 (56%) | 133,204,205 |
|  | AT1G08260 | LOC_Os02G30800 | 0 | 330/513 (64%) |  |
|  | AT5G22110 | LOC_Os05G06840 | 0 | 90/145 (62%) |  |
|  |  | LOC_Os08g36330 | 2.00E-49 | 294/588 (50%) |  |
| *POLH* | AT5G44740 | LOC_Os01G55300 | e-143 | 326/537 (60%) | 129 |
| *POLL* | AT1G10520 | LOC_Os06g13020 | e-178 | 1139/2205 (51%) | 126,135 |
| *REV1* | AT5G44750 | LOC_Os06g47580 | 0 | 76/134 (56%) | 130,131 |
| *REV3* | AT1G67500 | LOC_Os07g47280 | 2.00E-39 | 56/94 (59%) | 132 |
| *REV7* | AT1G16590 | LOC_Os04G40940 | 0.002 | 228/402 (56%) | 130 |
| PCNA | AT1G07370 | LOC_Os02G56130 | e-138 | 231/260 (88%) | 134,202 |
|  | AT2G29570 | LOC_Os02G56130 | e-139 | 234/262 (89%) |  |
| Polk | AT1G49980 | LOC_Os03g42010 | e-169 | 322/676 (47%) |  |
| **Rad6 pathway** |  |  |  |  |  |
| MMS2 | AT1G70660 | LOC_Os03G50440 | 3.00E-60 | 108/161 (67%) | 139 |
|  |  | LOC_Os12G41220 | 4.00E-57 | 99/134 (73%) |  |
|  |  | LOC_Os09g12570 | 6.00E-55 | 96/135 (71%) |  |
|  |  | LOC_Os04G58800 | 8.00E-52 | 93/142 (65%) |  |
|  | AT1G23260 | LOC_Os03G50440 | 3.00E-62 | 107/151 (70%) |  |
|  |  | LOC_Os12G41220 | 2.00E-57 | 98/140 (70%) |  |
|  |  | LOC_Os09g12570 | 3.00E-55 | 97/140 (69%) |  |
|  |  | LOC_Os04G58800 | 8.00E-54 | 95/140 (67%) |  |
|  | AT2G36060 | LOC_Os09g12570 | 2.00E-71 | 127/148 (85%) |  |
|  |  | LOC_Os12G41220 | 6.00E-67 | 116/142 (81%) |  |
|  |  | LOC_Os04G58800 | 6.00E-66 | 122/148 (82%) |  |
|  |  | LOC_Os03G50440 | 3.00E-55 | 100/139 (71%) |  |
|  | AT3G52560 | LOC_Os09g12570 | 6.00E-71 | 124/148 (83%) |  |
|  |  | LOC_Os12G41220 | 8.00E-66 | 112/139 (80%) |  |
|  |  | LOC_Os04G58800 | 5.00E-64 | 116/148 (78%) |  |
|  |  | LOC_Os03G50440 | 3.00E-58 | 104/143 (72%) |  |
| UBC1 | AT1G14400 | LOC_Os07g07240 | 7.00E-84 | 143/152 (94%) | 137-138,181 |
|  |  | LOC_Os05G08960 | 1.00E-83 | 143/152 (94%) |  |
|  |  | LOC_Os03G57790 | 3.00E-83 | 142/152 (93%) |  |
| UBC2 | AT2G02760 | LOC_Os07g07240 | 2.00E-76 | 135/152 (88%) |  |
|  |  | LOC_Os05G08960 | 2.00E-76 | 135/152 (88%) |  |
|  |  | LOC_Os03G57790 | 6.00E-76 | 134/152 (88%) |  |
| UBC3 | AT5G62540 | LOC_Os03G57790 | 3.00E-77 | 133/150 (88%) |  |
|  |  | LOC_Os05G08960 | 5.00E-77 | 133/150 (88%) |  |
|  |  | LOC_Os07g07240 | 5.00E-77 | 133/150 (88%) |  |
| UBE2N | AT1G16890 | LOC_Os01G48280 | 5.00E-66 | 117/120 (97%) | 140,186 |
|  | AT1G78870 | LOC_Os01G48280 | 4.00E-84 | 148/153 (96%) |  |
| **Direct reversal of damage** |  |  |  |  |  |
| *CRY1* | AT4G08920 | LOC_Os04G37920 | 0 | 449/692 (64%) | 165,167 |
|  |  | LOC_Os02G36380 | 0 | 436/701 (62%) |  |
| *CRY2* | AT1G04400 | LOC_Os02G41550 | 0 | 331/540 (61%) | 166-168 |
| *CRY3* | AT5G24850 | LOC_Os06g45100 | 0 | 336/507 (66%) | 169,170 |
| *PHR1* | AT1G12370 | LOC_Os10g08580 | e-176 | 316/486 (65%) | 171,172 |
| *PHR2* | AT2G47590 | LOC_Os03G22330 | e-110 | 185/331 (55%) | 173 |
| *AlkB* | AT1G11780 | LOC_Os11G29690 | 3.00E-52 | 93/129 (72%) |  |
| *ABH3/* *AlkB homolog 3* | AT2G22260 | LOC_Os06g17830 | 6.00E-83 | 145/219 (66%) |  |
| *UVR3* | AT3G15620 | LOC_Os02G10990 | 0 | 342/528 (64%) | 156 |
| **Genes defective in diseases associated with sensitivity to DNA damaging agents** |  |  |  |  |  |
| WRN | AT4G13870 | LOC_Os04g03990 | 3.00E-54 | 106/200 (53%) | 112,147, |
| ATM | AT3G48190 | LOC_Os01G01689 | 0 | 828/2251 (36%) | 148,149,150,151 |
| **Other conserved DNA damage response genes** |  |  |  |  |  |
| *RECQI1* | AT3G05740 | LOC_Os11g44910 | 0 | 370/601 (61%) | 141-143,146 |
| *RECQL2* | AT1G31360 | LOC_Os11g48090 | 0 | 473/714 (66%) |  |
| *RecQl3* | AT4G35740 | LOC_Os02G54020 | 0 | 374/718 (52%) |  |
| RECQSIM | AT5G27680 | LOC_Os05g05810 | 0 | 421/841 (50%) |  |
| *SNM1* | AT3G26680 | LOC_Os04G32930 | e-143 | 255/449 (56%) | 144,145 |
| *SNM1B* | AT1G27410 | LOC_Os01G57440 | e-100 | 184/374 (49%) |  |
| *RecQ886* | none | LOC_Os07g48360 |  |  | 146 |
| *RECQL5* | AT1G27880 | LOC_Os04g40970 | 0 | 361/774 (46%) | 142 |
| *ATR* | AT5G40820 | LOC_Os06g50910 | 0 | 1461/2753 (53%) | 149,152 |
| *RAD9* | AT3G05480 | LOC_Os03G22450 | e-113 | 223/438 (50%) |  |
| *RAD17* | AT5G66130 | LOC_Os03G13850 | e-119 | 230/490 (46%) | 153 |
| *CHEK1* | AT2G26980 | LOC_Os12G03810 | 0 | 321/435 (73%) |  |
| *CHEK2* | AT4G04720 | LOC_Os07g33110 | 0 | 370/462 (80%) |  |
| *CLK2A* | AT4G24740 | LOC_Os12G27520 | 0 | 301/424 (70%) |  |
| *CLK2B* | AT3G53570 | LOC_Os01G62080 | 0 | 334/435 (76%) |  |
| *CLK2C* | AT4G32660 | LOC_Os01G40840 | e-153 | 255/348 (73%) |  |
| *RAD1* | AT4G17760 | LOC_Os06g04190 | e-107 | 176/244 (72%) |  |
| *HUS1* | AT1G52530 | LOC_Os04G44620 | e-109 | 189/326 (57%) |  |
| *AXR1* | AT1G05180 | LOC_Os03G60550 | 0 | 355/522 (68%) |  |
| *SSRP1* | AT3G28730 | LOC_Os01G08970 | 0 | 322/477 (67%) |  |
|  |  | LOC_Os05G08970 | 0 | 313/477 (65%) |  |
| *PR19A/PUB60-1* | AT1G04510 | LOC_Os10g32880 | 0 | 370/527 (70%) |  |
| *PR19B/PUB60-2* | AT2G33340 | LOC_Os10g32880 | 0 | 358/507 (70%) |  |
| *SM3L2/RAD5a* | AT5G22750 | LOC_Os02G32570 | 0 | 606/868 (69%) | 157 |
| *SM3L3/ RAD5b* | AT5G43530 | LOC_Os04G09800 | 0 | 567/914 (62%) |  |
| *DRT100* | AT3G12610 | LOC_Os08g39550 | e-115 | 204/345 (59%) | 154 |
| *DRT102* | AT3G04880 | LOC_Os01G36090 | 8.00E-94 | 181/314 (57%) |  |
| *DRT111* | AT1G30480 | LOC_Os01G34190 | 2.00E-79 | 189/391 (48%) |  |
| *DRT101* | AT5G18070 | LOC_Os07g09720 | 0 | 305/542 (56%) |  |
| *DET1* | AT4G10180 | LOC_Os01G01484 | 0 | 329/543 (60%) | 23,25,28,197-199,201 |
| *COP1* | AT2G32950 | LOC_Os02G53140 | 0 | 473/674 (70%) | 155,165 |
| *BRU1* | AT3G18730 | LOC_Os02g54170 | e-103 | 216/453 (47%) | 159,160 |
|  |  | LOC_Os02g54280 | 0 | 497/1250 (39%) |  |
| *SMC1* | AT3G54670 | LOC_Os12g44390 | e-146 | 276/556 (49%) | 162-164,194-196 |
| *SMC2* | AT5G62410 | LOC_Os01g67740 | 0 | 738/1170 (63%) |  |
|  | AT3G47460 | LOC_Os01g67740 | 0 | 763/1170 (65%) |  |
| *SMC3* | AT2G27170 | LOC_Os02g04050 | 0 | 397/697 (56%) |  |
| *SMC4* | AT5G48600 | LOC_Os05g41750 | 0 | 327/477 (68%) |  |
| *SMC5* | AT5G15920 | LOC_Os05g51790 | 0 | 588/1057 (55%) |  |
| *SMC6* | AT5G07660 | LOC_Os09g03370 | 2e-044 | 82/117 (70%) |  |
| *PRD1* | AT4G14180 | LOC_Os04g28020 | 1e-066 | 143/337 (42%) | 158 |
| *REX1* | AT5G04910 | LOC_Os01g16080 | 1e-050 | 92/176 (52%) | 161 |

1.**Babiychuk E, Kushnir S, Van MM, Inzé D** (1994) The Arabidopsis thaliana apurinic endonuclease Arp reduces human transcription factors Fos and Jun.Proc Natl Acad Sci U S A**. 91(8):**3299-3303.

2.**Murphy TM, Belmonte M, Shu S, Britt AB, Hatteroth J** (2009) Requirement for Abasic Endonuclease Gene Homologues in Arabidopsis Seed Development. PLoS ONE 4(1): e4297.

3. **Murphy TM, Gao MJ** (2001) Multiple forms of formamidopyrimidine-DNA glycosylase produced by alternative splicing in [Arabidopsis](javascript:if(window.name=='') {{ window.location.href='./nil'; }} else {{ NPEml('MeSH',12328); }}) thaliana. J Photochem Photobiol B. **61(3):**87-93.

4. **Murphy TM** (2005) what’s base excision repair good for? Knockout mutants for FPG and OGG glycosylase genes in Arabidopsis. Physiol Plant. **123**:227–232.

5. **Murphy TM, George AA** (2005) Comparison of two DNA [base excision repair](javascript:if(window.name=='') {{ window.location.href='./nil'; }} else {{ NPEml('MeSH',823); }}) glycosylases from [Arabidopsis](javascript:if(window.name=='') {{ window.location.href='./nil'; }} else {{ NPEml('MeSH',12328); }}) thaliana. Biochem. Biophys.Res. Commun. **329(3):**869-872.

6. **Dany AL, Tissier AA** (2001) functional OGG1 homologue from Arabidopsis thaliana. Mol Genet Genomics. **265(2):**293-301.

7. **Roldan-Arjona T, Garcia-Ortiz MV, Ruiz-Rubio M, Ariza RR** (2000). cDNA cloning, expression and functional characterization of an Arabidopsis thaliana homologue of the Escherichia coli DNA repair enzyme endonuclease III. Plant Mol Biol. **44**: 43-52.

8. **Santerre A, Britt AB** (1994) Cloning of a 3-methyladenine-DNA glycosylase from Arabidopsis thaliana. *Proc. Natl. Acad. Sci. U.S.A.* **91**: 2240-2244

9**.Doucet-Chabeaud G, Godon C, Brutesco C, de Murcia G, Kazmaier,M** (2001) Ionising radiation induces the expression of PARP-1 and PARP-2 genes in Arabidopsis. Mol. Genet. Genomics **265**:954-963.

10.**Lepiniec L, Babiychuk E, Kushni S, van Montagu M, Inze D** (1995) Characterization of an Arabidopsis thaliana cDNA homologue to animal poly(ADP-ribose) polymerase. FEBS Lett. **364**:103-108.

11.**Gong Z, Morales-Ruiz T, Ariza RR, Roldan-Arjona T, David L, Zhu J-K** (2002) ROS1, a repressor of transcriptional gene silencing in Arabidopsis, encodes a DNA glycosylase/lyase. Cell **111**:803-814.

12.**Choi Y, Gehring M, Johnson L, Hannon M, Harada JJ, Goldberg RB, Jacobsen SE, Fischer RL** (2002) DEMETER, a DNA glycosylase domain protein, is required for endosperm gene imprinting and seed viability in Arabidopsis. Cell **110**:33-42.

13.**Morales-Ruiz T, Ortega-Galisteo AP, Ponferrada-Marin MI, Martinez-Macias MI, Ariza RR, Roldan-Arjona T** (2006) DEMETER and REPRESSOR OF SILENCING 1 encode 5-methylcytosine DNA glycosylases. Proc. Natl. Acad. Sci. U.S.A. **103**:6853-6858.

14. **Ortega-Galisteo AP, Morales-Ruiz T, Ariza RR, Roldán-Arjona T** (2008) Arabidopsis DEMETER-LIKE proteins DML2 and DML3 are required for appropriate distribution of DNA methylation marks. Plant Mol Biol. **67(6):**671-681.

15**. Lildballe DL, Pedersen DS, Kalamajka R, Emmersen J, Houben A, Grasser KD** (2008) The expression level of the chromatin-associated HMGB1 protein influences growth, stress tolerance, and transcriptome in Arabidopsis. J Mol Biol. **384(1)**:9-21.

16.**Launholt D, Merkle T, Houben A, Schulz A, Grasser KD** (2006) Arabidopsis chromatin-associated HMGA and HMGB use different nuclear targeting signals and display highly dynamic localization within the nucleus. Plant Cell **18(11):**2904-2918.

17.[**Uchiyama Y**](http://www.ncbi.nlm.nih.gov/sites/entrez?Db=pubmed&Cmd=Search&Term="Uchiyama Y"%5BAuthor%5D&itool=EntrezSystem2.PEntrez.Pubmed.Pubmed_ResultsPanel.Pubmed_DiscoveryPanel.Pubmed_RVAbstractPlus)**,** [**Suzuki Y**](http://www.ncbi.nlm.nih.gov/sites/entrez?Db=pubmed&Cmd=Search&Term="Suzuki Y"%5BAuthor%5D&itool=EntrezSystem2.PEntrez.Pubmed.Pubmed_ResultsPanel.Pubmed_DiscoveryPanel.Pubmed_RVAbstractPlus)**,** [**Sakaguchi K**](http://www.ncbi.nlm.nih.gov/sites/entrez?Db=pubmed&Cmd=Search&Term="Sakaguchi K"%5BAuthor%5D&itool=EntrezSystem2.PEntrez.Pubmed.Pubmed_ResultsPanel.Pubmed_DiscoveryPanel.Pubmed_RVAbstractPlus) (2008)Characterization of plant XRCC1 and its interaction with proliferating cell nuclear antigen. [Planta.](javascript:AL_get(this, 'jour', 'Planta.');) **227(6)**:1233-41.

18.[**García-Ortiz MV**](http://www.ncbi.nlm.nih.gov/sites/entrez?Db=pubmed&Cmd=Search&Term="García-Ortiz MV"%5BAuthor%5D&itool=EntrezSystem2.PEntrez.Pubmed.Pubmed_ResultsPanel.Pubmed_DiscoveryPanel.Pubmed_RVAbstractPlus)**,** [**Ariza RR**](http://www.ncbi.nlm.nih.gov/sites/entrez?Db=pubmed&Cmd=Search&Term="Ariza RR"%5BAuthor%5D&itool=EntrezSystem2.PEntrez.Pubmed.Pubmed_ResultsPanel.Pubmed_DiscoveryPanel.Pubmed_RVAbstractPlus)**,** [**Roldán-Arjona T**](http://www.ncbi.nlm.nih.gov/sites/entrez?Db=pubmed&Cmd=Search&Term="Roldán-Arjona T"%5BAuthor%5D&itool=EntrezSystem2.PEntrez.Pubmed.Pubmed_ResultsPanel.Pubmed_DiscoveryPanel.Pubmed_RVAbstractPlus) (2001) An OGG1 orthologue encoding a functional 8-oxoguanine DNA glycosylase/lyase in Arabidopsis thaliana. [Plant Mol Biol.](javascript:AL_get(this, 'jour', 'Plant Mol Biol.');) 47(6):795-804.

19.**Shimotohno A, Matsubayashi S, Yamaguchi M, Uchimiya H, Umeda M** (2003) Differential phosphorylation activities of CDK-activating kinases in Arabidopsis thaliana. FEBS Lett. **534**:69-74

20. **Shaked H, Avivi-Ragolsky N, Levy AA** (2006) Involvement of the Arabidopsis SWI2/SNF2 chromatin remodeling gene family in DNA damage response and recombination. Genetics 2006 **173(2):**985-994.

21.[**Al Khateeb WM**](http://www.ncbi.nlm.nih.gov/sites/entrez?Db=pubmed&Cmd=Search&Term="Al Khateeb WM"%5BAuthor%5D&itool=EntrezSystem2.PEntrez.Pubmed.Pubmed_ResultsPanel.Pubmed_DiscoveryPanel.Pubmed_RVAbstractPlus)**,** [**Schroeder DF**](http://www.ncbi.nlm.nih.gov/sites/entrez?Db=pubmed&Cmd=Search&Term="Schroeder DF"%5BAuthor%5D&itool=EntrezSystem2.PEntrez.Pubmed.Pubmed_ResultsPanel.Pubmed_DiscoveryPanel.Pubmed_RVAbstractPlus) (2009) Overexpression of Arabidopsis damaged DNA binding protein 1A (DDB1A) enhances UV tolerance. [Plant Mol Biol.](javascript:AL_get(this, 'jour', 'Plant Mol Biol.');) (in press)

22. **Zhang Y, Feng S, Chen F, Chen H, Wang J, McCall C, Xiong Y, Deng XW** ( 2008) Arabidopsis DDB1-CUL4 ASSOCIATED FACTOR1 forms a nuclear E3 ubiquitin ligase with DDB1 and CUL4 that is involved in multiple plant developmental processes. Plant Cell **20(6):**1437-1455.

23. **Al Khateeb WM, Schroeder DF** (2007) DDB2, DDB1A and DET1 exhibit complex interactions during Arabidopsis development. Genetics **176**:231-242.

24. **Molinier J, Lechner E, Dumbliauskas E, Genschik P** (2008) Regulation and role of Arabidopsis CUL4-DDB1A-DDB2 in maintaining genome integrity upon UV stress. PLoS Genet. **4**:0-E1000093.

25.**Bernhardt A, Lechner E, Hano P, Schade V, Dieterle M, Anders M, Dubin MJ, Benvenuto G, Bowler C, Genschik P, Hellmann H** (2006 ) CUL4 associates with DDB1 and DET1 and its downregulation affects diverse aspects of development in Arabidopsis thaliana. Plant J. **47(4):**591-603.

26.**Koga A, Ishibashi T, Kimura S, Uchiyama Y, Sakaguchi K** (2006) Characterization of T-DNA insertion mutants and RNAi silenced plants of Arabidopsis thaliana UV-damaged DNA binding protein 2 (AtUV-DDB2). Plant Mol Biol. **61(1-2):**227-240.

27.[**El-Mahdy MA**](http://www.ncbi.nlm.nih.gov/sites/entrez?Db=pubmed&Cmd=Search&Term="El-Mahdy MA"%5BAuthor%5D&itool=EntrezSystem2.PEntrez.Pubmed.Pubmed_ResultsPanel.Pubmed_DiscoveryPanel.Pubmed_RVAbstractPlus)**,** [**Zhu Q**](http://www.ncbi.nlm.nih.gov/sites/entrez?Db=pubmed&Cmd=Search&Term="Zhu Q"%5BAuthor%5D&itool=EntrezSystem2.PEntrez.Pubmed.Pubmed_ResultsPanel.Pubmed_DiscoveryPanel.Pubmed_RVAbstractPlus)**,** [**Wang QE**](http://www.ncbi.nlm.nih.gov/sites/entrez?Db=pubmed&Cmd=Search&Term="Wang QE"%5BAuthor%5D&itool=EntrezSystem2.PEntrez.Pubmed.Pubmed_ResultsPanel.Pubmed_DiscoveryPanel.Pubmed_RVAbstractPlus)**,** [**Wani G**](http://www.ncbi.nlm.nih.gov/sites/entrez?Db=pubmed&Cmd=Search&Term="Wani G"%5BAuthor%5D&itool=EntrezSystem2.PEntrez.Pubmed.Pubmed_ResultsPanel.Pubmed_DiscoveryPanel.Pubmed_RVAbstractPlus)**,** [**Praetorius-Ibba M**](http://www.ncbi.nlm.nih.gov/sites/entrez?Db=pubmed&Cmd=Search&Term="Praetorius-Ibba M"%5BAuthor%5D&itool=EntrezSystem2.PEntrez.Pubmed.Pubmed_ResultsPanel.Pubmed_DiscoveryPanel.Pubmed_RVAbstractPlus)**,** [**Wani AA**](http://www.ncbi.nlm.nih.gov/sites/entrez?Db=pubmed&Cmd=Search&Term="Wani AA"%5BAuthor%5D&itool=EntrezSystem2.PEntrez.Pubmed.Pubmed_ResultsPanel.Pubmed_DiscoveryPanel.Pubmed_RVAbstractPlus) (2006) Cullin 4A-mediated proteolysis of DDB2 protein at DNA damage sites regulates in vivo lesion recognition by XPC. [J Biol Chem.](javascript:AL_get(this, 'jour', 'J Biol Chem.');) **281(19):**13404-11.

28. **Schroeder DF, Gahrtz M, Maxwell BB, Cook RK, Kan JM, Alonso JM, Ecker JR, Chory J** (2002) De-etiolated 1 and damaged DNA binding protein 1 interact to regulate Arabidopsis photomorphogenesis. Curr. Biol. **12**:1462-1472.

29. **Taylor RM, Hamer MJ, Rosamond J, Bray CM** (1998) Molecular cloning and functional analysis of the Arabidopsis thaliana DNA ligase I homologue. Plant J. **14(1):** 75-81

30.**Harlow GR, Jenkins ME, Pittalwala TS, Mount DW** (1994) Isolation of uvh1, an Arabidopsis mutant hypersensitive to ultraviolet light and ionizing radiation. [Plant Cell.](javascript:AL_get(this, 'jour', 'Plant Cell.');) **6(2)**:227-235.

31.[**Fidantsef AL**](http://www.ncbi.nlm.nih.gov/sites/entrez?Db=pubmed&Cmd=Search&Term="Fidantsef AL"%5BAuthor%5D&itool=EntrezSystem2.PEntrez.Pubmed.Pubmed_ResultsPanel.Pubmed_RVAbstract)**,** [**Mitchell DL**](http://www.ncbi.nlm.nih.gov/sites/entrez?Db=pubmed&Cmd=Search&Term="Mitchell DL"%5BAuthor%5D&itool=EntrezSystem2.PEntrez.Pubmed.Pubmed_ResultsPanel.Pubmed_RVAbstract)**,** [**Britt AB**](http://www.ncbi.nlm.nih.gov/sites/entrez?Db=pubmed&Cmd=Search&Term="Britt AB"%5BAuthor%5D&itool=EntrezSystem2.PEntrez.Pubmed.Pubmed_ResultsPanel.Pubmed_RVAbstract) (2000) The Arabidopsis UVH1 gene is a homolog of the yeast repair endonuclease RAD1.[Plant Physiol.](javascript:AL_get(this, 'jour', 'Plant Physiol.');) **124(2):**579-586.

32.[**Liu Z**](http://www.ncbi.nlm.nih.gov/sites/entrez?Db=pubmed&Cmd=Search&Term="Liu Z"%5BAuthor%5D&itool=EntrezSystem2.PEntrez.Pubmed.Pubmed_ResultsPanel.Pubmed_RVAbstract)**,** [**Hossain GS**](http://www.ncbi.nlm.nih.gov/sites/entrez?Db=pubmed&Cmd=Search&Term="Hossain GS"%5BAuthor%5D&itool=EntrezSystem2.PEntrez.Pubmed.Pubmed_ResultsPanel.Pubmed_RVAbstract)**,** [**Islas-Osuna MA**](http://www.ncbi.nlm.nih.gov/sites/entrez?Db=pubmed&Cmd=Search&Term="Islas-Osuna MA"%5BAuthor%5D&itool=EntrezSystem2.PEntrez.Pubmed.Pubmed_ResultsPanel.Pubmed_RVAbstract)**,** [**Mitchell DL**](http://www.ncbi.nlm.nih.gov/sites/entrez?Db=pubmed&Cmd=Search&Term="Mitchell DL"%5BAuthor%5D&itool=EntrezSystem2.PEntrez.Pubmed.Pubmed_ResultsPanel.Pubmed_RVAbstract)**,** [**Mount DW**](http://www.ncbi.nlm.nih.gov/sites/entrez?Db=pubmed&Cmd=Search&Term="Mount DW"%5BAuthor%5D&itool=EntrezSystem2.PEntrez.Pubmed.Pubmed_ResultsPanel.Pubmed_RVAbstract)( 2000) Repair of UV damage in plants by nucleotide excision repair: Arabidopsis UVH1 DNA repair gene is a homolog of Saccharomyces cerevisiae Rad1.Plant J. **21(6):**519-528.

33.[**Vonarx EJ**](http://www.ncbi.nlm.nih.gov/sites/entrez?Db=pubmed&Cmd=Search&Term="Vonarx EJ"%5BAuthor%5D&itool=EntrezSystem2.PEntrez.Pubmed.Pubmed_ResultsPanel.Pubmed_RVAbstract)**,** [**Howlett NG**](http://www.ncbi.nlm.nih.gov/sites/entrez?Db=pubmed&Cmd=Search&Term="Howlett NG"%5BAuthor%5D&itool=EntrezSystem2.PEntrez.Pubmed.Pubmed_ResultsPanel.Pubmed_RVAbstract)**,** [**Schiestl RH**](http://www.ncbi.nlm.nih.gov/sites/entrez?Db=pubmed&Cmd=Search&Term="Schiestl RH"%5BAuthor%5D&itool=EntrezSystem2.PEntrez.Pubmed.Pubmed_ResultsPanel.Pubmed_RVAbstract)**,** [**Kunz BA**](http://www.ncbi.nlm.nih.gov/sites/entrez?Db=pubmed&Cmd=Search&Term="Kunz BA"%5BAuthor%5D&itool=EntrezSystem2.PEntrez.Pubmed.Pubmed_ResultsPanel.Pubmed_RVAbstract) (2002) Detection of Arabidopsis thaliana AtRAD1 cDNA variants and assessment of function by expression in a yeast rad1 mutant. [Gene](javascript:AL_get(this, 'jour', 'Gene.');) **296(1-2)**:1-9.

34.[**Lechner E**](http://www.uniprot.org/uniprot/?query=author:"Lechner+E.")**,** [**Xie D**](http://www.uniprot.org/uniprot/?query=author:"Xie+D.")**,** [**Grava S**](http://www.uniprot.org/uniprot/?query=author:"Grava+S.")**,** [**Pigaglio E**](http://www.uniprot.org/uniprot/?query=author:"Pigaglio+E.")**,** [**Planchais S**](http://www.uniprot.org/uniprot/?query=author:"Planchais+S.")**,** [**Murray JAH**](http://www.uniprot.org/uniprot/?query=author:"Murray+J.A.H.")**,** [**Parmentier Y**](http://www.uniprot.org/uniprot/?query=author:"Parmentier+Y.")**,** [**Mutterer J**](http://www.uniprot.org/uniprot/?query=author:"Mutterer+J.")**,** [**Dubreucq B**](http://www.uniprot.org/uniprot/?query=author:"Dubreucq+B.")**,** [**Shen W-H**](http://www.uniprot.org/uniprot/?query=author:"Shen+W.-H.")**, Genschik P** (2002) **The AtRbx1 protein is part of plant SCF complexes, and its down-regulation causes severe growth and developmental defects.** J. Biol. Chem. **277**:50069-50080.

35.**Xia R, Wang J, Liu C, Wang Y, Wang Y, Zhai J, Liu J, Hong X, Cao X, Zhu JK, Gong Z** (2006) ROR1/RPA2A, a putative replication protein A2, functions in epigenetic gene silencing and in regulation of meristem development in Arabidopsis. Plant Cell. **18(1):**85-103.

36. **Elmayan T, Proux F, Vaucheret H** (2005) Arabidopsis RPA2: a genetic link among transcriptional gene silencing, DNA repair, and DNA replication. Curr Biol. **15(21):**1919-1925.

37.**Osman K, Sanchez-Moran E, Mann SC, Jones GH, Franklin FC** (2009) Replication protein A (AtRPA1a) is required for class I crossover formation but is dispensable for meiotic DNA break repair. EMBO J. **28(4):**394-404.

38.[**Furukawa T**](http://www.ncbi.nlm.nih.gov/sites/entrez?Db=pubmed&Cmd=Search&Term="Furukawa T"%5BAuthor%5D&itool=EntrezSystem2.PEntrez.Pubmed.Pubmed_ResultsPanel.Pubmed_DiscoveryPanel.Pubmed_RVAbstractPlus)**,** [**Ishibashi T**](http://www.ncbi.nlm.nih.gov/sites/entrez?Db=pubmed&Cmd=Search&Term="Ishibashi T"%5BAuthor%5D&itool=EntrezSystem2.PEntrez.Pubmed.Pubmed_ResultsPanel.Pubmed_DiscoveryPanel.Pubmed_RVAbstractPlus)**,** [**Kimura S**](http://www.ncbi.nlm.nih.gov/sites/entrez?Db=pubmed&Cmd=Search&Term="Kimura S"%5BAuthor%5D&itool=EntrezSystem2.PEntrez.Pubmed.Pubmed_ResultsPanel.Pubmed_DiscoveryPanel.Pubmed_RVAbstractPlus)**,** [**Tanaka H**](http://www.ncbi.nlm.nih.gov/sites/entrez?Db=pubmed&Cmd=Search&Term="Tanaka H"%5BAuthor%5D&itool=EntrezSystem2.PEntrez.Pubmed.Pubmed_ResultsPanel.Pubmed_DiscoveryPanel.Pubmed_RVAbstractPlus)**,** [**Hashimoto J**](http://www.ncbi.nlm.nih.gov/sites/entrez?Db=pubmed&Cmd=Search&Term="Hashimoto J"%5BAuthor%5D&itool=EntrezSystem2.PEntrez.Pubmed.Pubmed_ResultsPanel.Pubmed_DiscoveryPanel.Pubmed_RVAbstractPlus)**,** [**Sakaguchi K**](http://www.ncbi.nlm.nih.gov/sites/entrez?Db=pubmed&Cmd=Search&Term="Sakaguchi K"%5BAuthor%5D&itool=EntrezSystem2.PEntrez.Pubmed.Pubmed_ResultsPanel.Pubmed_DiscoveryPanel.Pubmed_RVAbstractPlus) (2003) Characterization of all the subunits of replication factor C from a higher plant, rice (Oryza sativa L.), and their relation to development. Plant Mol Biol. **53(1-2)**:15-25

39. **Liu Z, Hong S-W, Escobar M, Vierling E, Mitchell DL, Mount DW, Hall JD** (2003) Arabidopsis UVH6, a homolog of human XPD and yeast RAD3 DNA repair genes, functions in DNA repair and is essential for plant growth. Plant Physiol. **132**:1405-1414.

40.**Vonarx EJ, Tabone EK, Osmond MJ, Anderson HJ, Kunz BA** (2006 ) Arabidopsis homologue of human transcription factor IIH/nucleotide excision repair factor p44 can function in transcription and DNA repair and interacts with AtXPD. Plant J. **46(3):**512-521.

41. **Dubest S, Gallego ME, White CI** (2004) Roles of the AtErcc1 protein in recombination. Plant J. **39**:334-342

42.**Hefner E, Preuss SB, Britt AB** (2003) Arabidopsis mutants sensitive to gamma radiation includes the homologue of the human repair gene ERCC1. J Exp Bot. **54(383)**:669-680.

43.[**Vannier JB**](http://www.ncbi.nlm.nih.gov/sites/entrez?Db=pubmed&Cmd=Search&Term="Vannier JB"%5BAuthor%5D&itool=EntrezSystem2.PEntrez.Pubmed.Pubmed_ResultsPanel.Pubmed_DiscoveryPanel.Pubmed_RVAbstractPlus)**,** [**Depeiges A**](http://www.ncbi.nlm.nih.gov/sites/entrez?Db=pubmed&Cmd=Search&Term="Depeiges A"%5BAuthor%5D&itool=EntrezSystem2.PEntrez.Pubmed.Pubmed_ResultsPanel.Pubmed_DiscoveryPanel.Pubmed_RVAbstractPlus)**,** [**White C**](http://www.ncbi.nlm.nih.gov/sites/entrez?Db=pubmed&Cmd=Search&Term="White C"%5BAuthor%5D&itool=EntrezSystem2.PEntrez.Pubmed.Pubmed_ResultsPanel.Pubmed_DiscoveryPanel.Pubmed_RVAbstractPlus)**,** [**Gallego ME**](http://www.ncbi.nlm.nih.gov/sites/entrez?Db=pubmed&Cmd=Search&Term="Gallego ME"%5BAuthor%5D&itool=EntrezSystem2.PEntrez.Pubmed.Pubmed_ResultsPanel.Pubmed_DiscoveryPanel.Pubmed_RVAbstractPlus) (2009) ERCC1/XPF protects short telomeres from homologous recombination in Arabidopsis thaliana. [PLoS Genet.](javascript:AL_get(this, 'jour', 'PLoS Genet.');) **5(2)**:e1000380.

44..**Ribeiro DT, Machado CR, Costa RMA, Praekelt UM, Van Sluys M-A, Menck CFM** (1998) Cloning of a cDNA from Arabidopsis thaliana homologous to the human XPB gene. Gene **208**:207-213.

45.**Morgante PG, Berra CM, Nakabashi M, Costa RMA, Menck CFM, Van Sluys M-A** (2005)Functional XPB/RAD25 redundancy in Arabidopsis genome: characterization of AtXPB2 and expression analysis. Gene **344**:93-103.

46.**Costa RMA, Morgante PG, Berra CM, Nakabashi M, Bruneau D, Bouchez D, Sweder KS, Van Sluys M-A, Menck CFM** (2001) The participation of AtXPB1, the XPB/RAD25 homologue gene from Arabidopsis thaliana, in DNA repair and plant development. Plant J. **28**:385-395.

47. **Liu Z, Hall JD, Mount DW** (2001) Arabidopsis UVH3 gene is a homolog of the Saccharomyces cerevisiae RAD2 and human XPG DNA repair genes.
Plant J. **26**:329-338

48. **Schultz TF, Quatrano RS** (1997) Characterization and expression of a rice RAD23 gene. Plant Mol Biol. **34(3):**557-562.

49.**Liang L, Flury S, Kalck V, Hohn B, Molinier J** (2006) CENTRIN2 interacts with the Arabidopsis homolog of the human XPC protein (AtRAD4) and contributes to efficient synthesis-dependent repair of bulky DNA lesions. Plant Mol Biol.**61 (1-2):**345-356.

50.[**Molinier J**](http://www.ncbi.nlm.nih.gov/sites/entrez?Db=pubmed&Cmd=Search&Term="Molinier J"%5BAuthor%5D&itool=EntrezSystem2.PEntrez.Pubmed.Pubmed_ResultsPanel.Pubmed_DiscoveryPanel.Pubmed_RVAbstractPlus)**,** [**Ramos C**](http://www.ncbi.nlm.nih.gov/sites/entrez?Db=pubmed&Cmd=Search&Term="Ramos C"%5BAuthor%5D&itool=EntrezSystem2.PEntrez.Pubmed.Pubmed_ResultsPanel.Pubmed_DiscoveryPanel.Pubmed_RVAbstractPlus)**,** [**Fritsch O**](http://www.ncbi.nlm.nih.gov/sites/entrez?Db=pubmed&Cmd=Search&Term="Fritsch O"%5BAuthor%5D&itool=EntrezSystem2.PEntrez.Pubmed.Pubmed_ResultsPanel.Pubmed_DiscoveryPanel.Pubmed_RVAbstractPlus)**,** [**Hohn B**](http://www.ncbi.nlm.nih.gov/sites/entrez?Db=pubmed&Cmd=Search&Term="Hohn B"%5BAuthor%5D&itool=EntrezSystem2.PEntrez.Pubmed.Pubmed_ResultsPanel.Pubmed_DiscoveryPanel.Pubmed_RVAbstractPlus) (2004)CENTRIN2 modulates homologous recombination and nucleotide excision repair in Arabidopsis. [Plant Cell.](javascript:AL_get(this, 'jour', 'Plant Cell.');) **16(6)**:1633-1643.

51. **Lafarge S and Montane MH** (2003). Characterization of Arabidopsis thaliana ortholog of the human breast cancer susceptibility gene 1: AtBRCA1, strongly induced by gamma rays. Nucleic Acids Research **31**: 1148-1155.

52. **Siaud N, Dray E, Gy I, Gérard E, Takvorian N, Doutriaux MP** (2004) Brca2 is involved in meiosis in Arabidopsis thaliana as suggested by its interaction with Dmc1. EMBO J. **23(6):**1392-1401.

53.[**Dray E**](http://www.ncbi.nlm.nih.gov/sites/entrez?Db=pubmed&Cmd=Search&Term="Dray E"%5BAuthor%5D&itool=EntrezSystem2.PEntrez.Pubmed.Pubmed_ResultsPanel.Pubmed_RVAbstract)**,** [**Siaud N**](http://www.ncbi.nlm.nih.gov/sites/entrez?Db=pubmed&Cmd=Search&Term="Siaud N"%5BAuthor%5D&itool=EntrezSystem2.PEntrez.Pubmed.Pubmed_ResultsPanel.Pubmed_RVAbstract)**,** [**Dubois E**](http://www.ncbi.nlm.nih.gov/sites/entrez?Db=pubmed&Cmd=Search&Term="Dubois E"%5BAuthor%5D&itool=EntrezSystem2.PEntrez.Pubmed.Pubmed_ResultsPanel.Pubmed_RVAbstract)**,** [**Doutriaux MP**](http://www.ncbi.nlm.nih.gov/sites/entrez?Db=pubmed&Cmd=Search&Term="Doutriaux MP"%5BAuthor%5D&itool=EntrezSystem2.PEntrez.Pubmed.Pubmed_ResultsPanel.Pubmed_RVAbstract) (2006) Interaction between Arabidopsis Brca2 and its partners Rad51, Dmc1, and Dss1.[Plant Physiol.](javascript:AL_get(this, 'jour', 'Plant Physiol.');) **140(3)**:1059-1069.

54. **Lohmiller LD, De Muyt A, Howard B, Offenberg HH, Heyting C, Grelon M, Anderson LK** (2008 ) Cytological analysis of MRE11 protein during early meiotic prophase I in Arabidopsis and tomato. Chromosoma **117(3):**277-288.

55.[**Puizina J**](http://www.ncbi.nlm.nih.gov/sites/entrez?Db=pubmed&Cmd=Search&Term="Puizina J"%5BAuthor%5D&itool=EntrezSystem2.PEntrez.Pubmed.Pubmed_ResultsPanel.Pubmed_RVAbstract)**,** [**Siroky J**](http://www.ncbi.nlm.nih.gov/sites/entrez?Db=pubmed&Cmd=Search&Term="Siroky J"%5BAuthor%5D&itool=EntrezSystem2.PEntrez.Pubmed.Pubmed_ResultsPanel.Pubmed_RVAbstract)**,** [**Mokros P**](http://www.ncbi.nlm.nih.gov/sites/entrez?Db=pubmed&Cmd=Search&Term="Mokros P"%5BAuthor%5D&itool=EntrezSystem2.PEntrez.Pubmed.Pubmed_ResultsPanel.Pubmed_RVAbstract)**,** [**Schweizer D**](http://www.ncbi.nlm.nih.gov/sites/entrez?Db=pubmed&Cmd=Search&Term="Schweizer D"%5BAuthor%5D&itool=EntrezSystem2.PEntrez.Pubmed.Pubmed_ResultsPanel.Pubmed_RVAbstract)**,** [**Riha K**](http://www.ncbi.nlm.nih.gov/sites/entrez?Db=pubmed&Cmd=Search&Term="Riha K"%5BAuthor%5D&itool=EntrezSystem2.PEntrez.Pubmed.Pubmed_ResultsPanel.Pubmed_RVAbstract) (2004) Mre11 deficiency in Arabidopsis is associated with chromosomal instability in somatic cells and Spo11-dependent genome fragmentation during meiosis. [Plant Cell](javascript:AL_get(this, 'jour', 'Plant Cell.');) **16(8):**1968-1978.

56. **Bundock P and Hooykaas P** (2002) Severe developmental defects, hypersensitivity to DNA-damaging agents, and lengthened telomeres in Arabidopsis MRE11 mutants. Plant Cell **14**: 2451-62.

57.[**Hartung F**](http://www.ncbi.nlm.nih.gov/sites/entrez?Db=pubmed&Cmd=Search&Term="Hartung F"%5BAuthor%5D&itool=EntrezSystem2.PEntrez.Pubmed.Pubmed_ResultsPanel.Pubmed_RVAbstract)**,** [**Suer S**](http://www.ncbi.nlm.nih.gov/sites/entrez?Db=pubmed&Cmd=Search&Term="Suer S"%5BAuthor%5D&itool=EntrezSystem2.PEntrez.Pubmed.Pubmed_ResultsPanel.Pubmed_RVAbstract)**,** [**Bergmann T**](http://www.ncbi.nlm.nih.gov/sites/entrez?Db=pubmed&Cmd=Search&Term="Bergmann T"%5BAuthor%5D&itool=EntrezSystem2.PEntrez.Pubmed.Pubmed_ResultsPanel.Pubmed_RVAbstract)**,** [**Puchta H**](http://www.ncbi.nlm.nih.gov/sites/entrez?Db=pubmed&Cmd=Search&Term="Puchta H"%5BAuthor%5D&itool=EntrezSystem2.PEntrez.Pubmed.Pubmed_ResultsPanel.Pubmed_RVAbstract) (2006)The role of AtMUS81 in DNA repair and its genetic interaction with the helicase AtRecQ4A. [Nucleic Acids Res.](javascript:AL_get(this, 'jour', 'Nucleic Acids Res.');) **34(16):**4438-4448.

58. **Higgins JD, Buckling EF, Franklin FC, Jones GH** (2008) Expression and functional analysis of AtMUS81 in Arabidopsis meiosis reveals a role in the second pathway of crossing-over. Plant J*.* **54**:152-162.

59.[**Geuting V**](http://www.ncbi.nlm.nih.gov/sites/entrez?Db=pubmed&Cmd=Search&Term="Geuting V"%5BAuthor%5D&itool=EntrezSystem2.PEntrez.Pubmed.Pubmed_ResultsPanel.Pubmed_DiscoveryPanel.Pubmed_RVAbstractPlus)**,** [**Kobbe D**](http://www.ncbi.nlm.nih.gov/sites/entrez?Db=pubmed&Cmd=Search&Term="Kobbe D"%5BAuthor%5D&itool=EntrezSystem2.PEntrez.Pubmed.Pubmed_ResultsPanel.Pubmed_DiscoveryPanel.Pubmed_RVAbstractPlus)**,** [**Hartung F**](http://www.ncbi.nlm.nih.gov/sites/entrez?Db=pubmed&Cmd=Search&Term="Hartung F"%5BAuthor%5D&itool=EntrezSystem2.PEntrez.Pubmed.Pubmed_ResultsPanel.Pubmed_DiscoveryPanel.Pubmed_RVAbstractPlus)**,** [**Durr J**](http://www.ncbi.nlm.nih.gov/sites/entrez?Db=pubmed&Cmd=Search&Term="Durr J"%5BAuthor%5D&itool=EntrezSystem2.PEntrez.Pubmed.Pubmed_ResultsPanel.Pubmed_DiscoveryPanel.Pubmed_RVAbstractPlus)**,** [**Focke M**](http://www.ncbi.nlm.nih.gov/sites/entrez?Db=pubmed&Cmd=Search&Term="Focke M"%5BAuthor%5D&itool=EntrezSystem2.PEntrez.Pubmed.Pubmed_ResultsPanel.Pubmed_DiscoveryPanel.Pubmed_RVAbstractPlus)**,** [**Puchta H**](http://www.ncbi.nlm.nih.gov/sites/entrez?Db=pubmed&Cmd=Search&Term="Puchta H"%5BAuthor%5D&itool=EntrezSystem2.PEntrez.Pubmed.Pubmed_ResultsPanel.Pubmed_DiscoveryPanel.Pubmed_RVAbstractPlus) (2009) Two distinct MUS81-EME1 complexes from Arabidopsis thaliana process Holliday junctions. [Plant Physiol.](javascript:AL_get(this, 'jour', 'Plant Physiol.');) (in press)

60. **Gallego ME, Jeanneau M, Granier F, Bouchez D, Bechtold N, White CI** (2001) Disruption of the Arabidopsis RAD50 gene leads to plant sterility and MMS sensitivity. Plant J. **25**:31-41.

61. **Gherbi H, Gallego ME, Jalut N, Lucht JM, Hohn B, White CI** (2001) Homologous recombination in planta is stimulated in the absence of Rad50. EMBO Rep*.* **2**:287-291.

62. **Gallego ME, White CI** (2001) RAD50 function is essential for telomere maintenance in Arabidopsis. Proc. Natl. Acad. Sci. U.S.A**. 98**:1711-1716.

63.**Bleuyard J-Y, Gallego ME, White CI** (2004) Meiotic defects in the Arabidopsis rad50 mutant point to conservation of the MRX complex function in early stages of meiotic recombination. Chromosoma **113**:197-203.

64.**Vannier JB, Depeiges A, White C, Gallego ME** (2006) Two roles for Rad50 in telomere maintenance. EMBO J. **25(19)**:4577-4585.

65.[**Bleuyard JY**](http://www.ncbi.nlm.nih.gov/sites/entrez?Db=pubmed&Cmd=Search&Term="Bleuyard JY"%5BAuthor%5D&itool=EntrezSystem2.PEntrez.Pubmed.Pubmed_ResultsPanel.Pubmed_RVAbstract)**,** [**Gallego ME**](http://www.ncbi.nlm.nih.gov/sites/entrez?Db=pubmed&Cmd=Search&Term="Gallego ME"%5BAuthor%5D&itool=EntrezSystem2.PEntrez.Pubmed.Pubmed_ResultsPanel.Pubmed_RVAbstract)**,** [**Savigny F**](http://www.ncbi.nlm.nih.gov/sites/entrez?Db=pubmed&Cmd=Search&Term="Savigny F"%5BAuthor%5D&itool=EntrezSystem2.PEntrez.Pubmed.Pubmed_ResultsPanel.Pubmed_RVAbstract)**,** [**White CI**](http://www.ncbi.nlm.nih.gov/sites/entrez?Db=pubmed&Cmd=Search&Term="White CI"%5BAuthor%5D&itool=EntrezSystem2.PEntrez.Pubmed.Pubmed_ResultsPanel.Pubmed_RVAbstract) (2005) Differing requirements for the Arabidopsis Rad51 paralogs in meiosis and DNA repair. [Plant J.](javascript:AL_get(this, 'jour', 'Plant J.');) **41(4):**533-445.

66.**Osakabe K, Abe K, Yamanouchi H, Takyuu T, Yoshioka T, Ito Y, Kato T, Tabata S, Kurei S, Yoshioka Y, Machida Y, Seki M, Kobayashi M, Shinozaki K, Ichikawa H, Toki S** (2005) Arabidopsis Rad51B is important for double-strand DNA breaks repair in somatic cells. Plant Mol Biol. **57(6):**819-833.

67.**Osakabe K, Yoshioka T, Ichikawa H, Toki S** (2002) Molecular cloning and characterization of RAD51-like genes from Arabidopsis thaliana. Plant Mol. Biol. **50**:71-81.

68.**Abe K, Osakabe K, Nakayama S, Endo M, Tagiri A, Todoriki S, Ichikawa H, Toki S** (2005 ) Arabidopsis RAD51C gene is important for homologous recombination in meiosis and mitosis. Plant Physiol. **139(2):**896-908.

69.**Li W, Yang X, Lin Z, Timofejeva L, Xiao R, Makaroff CA, Ma H** (2005) The AtRAD51C gene is required for normal meiotic chromosome synapsis and double-stranded break repair in Arabidopsis. Plant Physiol.**138 (2):**965-976.

70.[**Abe K**](http://www.ncbi.nlm.nih.gov/sites/entrez?Db=pubmed&Cmd=Search&Term="Abe K"%5BAuthor%5D&itool=EntrezSystem2.PEntrez.Pubmed.Pubmed_ResultsPanel.Pubmed_RVAbstract)**,** [**Osakabe K**](http://www.ncbi.nlm.nih.gov/sites/entrez?Db=pubmed&Cmd=Search&Term="Osakabe K"%5BAuthor%5D&itool=EntrezSystem2.PEntrez.Pubmed.Pubmed_ResultsPanel.Pubmed_RVAbstract)**,** [**Nakayama S**](http://www.ncbi.nlm.nih.gov/sites/entrez?Db=pubmed&Cmd=Search&Term="Nakayama S"%5BAuthor%5D&itool=EntrezSystem2.PEntrez.Pubmed.Pubmed_ResultsPanel.Pubmed_RVAbstract)**,** [**Endo M**](http://www.ncbi.nlm.nih.gov/sites/entrez?Db=pubmed&Cmd=Search&Term="Endo M"%5BAuthor%5D&itool=EntrezSystem2.PEntrez.Pubmed.Pubmed_ResultsPanel.Pubmed_RVAbstract)**,** [**Tagiri A**](http://www.ncbi.nlm.nih.gov/sites/entrez?Db=pubmed&Cmd=Search&Term="Tagiri A"%5BAuthor%5D&itool=EntrezSystem2.PEntrez.Pubmed.Pubmed_ResultsPanel.Pubmed_RVAbstract)**,** [**Todoriki S**](http://www.ncbi.nlm.nih.gov/sites/entrez?Db=pubmed&Cmd=Search&Term="Todoriki S"%5BAuthor%5D&itool=EntrezSystem2.PEntrez.Pubmed.Pubmed_ResultsPanel.Pubmed_RVAbstract)**,** [**Ichikawa H**](http://www.ncbi.nlm.nih.gov/sites/entrez?Db=pubmed&Cmd=Search&Term="Ichikawa H"%5BAuthor%5D&itool=EntrezSystem2.PEntrez.Pubmed.Pubmed_ResultsPanel.Pubmed_RVAbstract)**,** [**Toki S**](http://www.ncbi.nlm.nih.gov/sites/entrez?Db=pubmed&Cmd=Search&Term="Toki S"%5BAuthor%5D&itool=EntrezSystem2.PEntrez.Pubmed.Pubmed_ResultsPanel.Pubmed_RVAbstract) (2005) Arabidopsis RAD51C gene is important for homologous recombination in meiosis and mitosis. [Plant Physiol.](javascript:AL_get(this, 'jour', 'Plant Physiol.');) **139(2):**896-908.

71. **Durrant WE, Wang S, Dong X** (2007) Arabidopsis SNI1 and RAD51D regulate both gene transcription and DNA recombination during the defense response. Proc Natl Acad Sci U S A. **104(10):**4223-4227.

72.**Doutriaux MP, Couteau F, Bergounioux C, White C** (1998) Isolation and characterisation of the RAD51 and DMC1 homologs from Arabidopsis thaliana. *Mol.* Gen. Genet. **257**:283-291.

73. **Li W, Chen C, Markmann-Mulisch U, Timofejeva L, Schmelzer E, Ma H, Reiss B** (2004) The Arabidopsis AtRAD51 gene is dispensable for vegetative development but required for meiosis. Proc. Natl. Acad. Sci. U.S.A. **101**:10596-10601

74.**Markmann-Mulisch U, Wendeler E, Zobell O, Schween G, Steinbiss HH, Reiss B** (2007) Differential requirements for RAD51 in Physcomitrella patens and Arabidopsis thaliana development and DNA damage repair. Plant Cell **19(10)**:3080-3089.

75.**Klutstein M, Shaked H, Sherman A, Avivi-Ragolsky N, Shema E, Zenvirth D, Levy AA, Simchen G** (2008 )Functional conservation of the yeast and Arabidopsis RAD54-like genes. Genetics **178(4):**2389-2397

76.**Osakabe K, Abe K, Yoshioka T, Osakabe Y, Todoriki S, Ichikawa H, Hohn B, Toki S** (2006) Isolation and characterization of the RAD54 gene from Arabidopsis thaliana.Plant J. **48(6):**827-842.

77.[**Akutsu N**](http://www.ncbi.nlm.nih.gov/sites/entrez?Db=pubmed&Cmd=Search&Term="Akutsu N"%5BAuthor%5D&itool=EntrezSystem2.PEntrez.Pubmed.Pubmed_ResultsPanel.Pubmed_RVAbstract)**,** [**Iijima K**](http://www.ncbi.nlm.nih.gov/sites/entrez?Db=pubmed&Cmd=Search&Term="Iijima K"%5BAuthor%5D&itool=EntrezSystem2.PEntrez.Pubmed.Pubmed_ResultsPanel.Pubmed_RVAbstract)**,** [**Hinata T**](http://www.ncbi.nlm.nih.gov/sites/entrez?Db=pubmed&Cmd=Search&Term="Hinata T"%5BAuthor%5D&itool=EntrezSystem2.PEntrez.Pubmed.Pubmed_ResultsPanel.Pubmed_RVAbstract)**,** [**Tauchi H**](http://www.ncbi.nlm.nih.gov/sites/entrez?Db=pubmed&Cmd=Search&Term="Tauchi H"%5BAuthor%5D&itool=EntrezSystem2.PEntrez.Pubmed.Pubmed_ResultsPanel.Pubmed_RVAbstract) (2007)Characterization of the plant homolog of Nijmegen breakage syndrome 1: Involvement in DNA repair and recombination. [Biochem Biophys Res Commun.](javascript:AL_get(this, 'jour', 'Biochem Biophys Res Commun.');) **353(2):**394-398.

78.[**Waterworth WM**](http://www.ncbi.nlm.nih.gov/sites/entrez?Db=pubmed&Cmd=Search&Term="Waterworth WM"%5BAuthor%5D&itool=EntrezSystem2.PEntrez.Pubmed.Pubmed_ResultsPanel.Pubmed_RVAbstract)**,** [**Altun C**](http://www.ncbi.nlm.nih.gov/sites/entrez?Db=pubmed&Cmd=Search&Term="Altun C"%5BAuthor%5D&itool=EntrezSystem2.PEntrez.Pubmed.Pubmed_ResultsPanel.Pubmed_RVAbstract)**,** [**Armstrong SJ**](http://www.ncbi.nlm.nih.gov/sites/entrez?Db=pubmed&Cmd=Search&Term="Armstrong SJ"%5BAuthor%5D&itool=EntrezSystem2.PEntrez.Pubmed.Pubmed_ResultsPanel.Pubmed_RVAbstract)**,** [**Roberts N**](http://www.ncbi.nlm.nih.gov/sites/entrez?Db=pubmed&Cmd=Search&Term="Roberts N"%5BAuthor%5D&itool=EntrezSystem2.PEntrez.Pubmed.Pubmed_ResultsPanel.Pubmed_RVAbstract)**,** [**Dean PJ**](http://www.ncbi.nlm.nih.gov/sites/entrez?Db=pubmed&Cmd=Search&Term="Dean PJ"%5BAuthor%5D&itool=EntrezSystem2.PEntrez.Pubmed.Pubmed_ResultsPanel.Pubmed_RVAbstract)**,** [**Young K**](http://www.ncbi.nlm.nih.gov/sites/entrez?Db=pubmed&Cmd=Search&Term="Young K"%5BAuthor%5D&itool=EntrezSystem2.PEntrez.Pubmed.Pubmed_ResultsPanel.Pubmed_RVAbstract)**,** [**Weil CF**](http://www.ncbi.nlm.nih.gov/sites/entrez?Db=pubmed&Cmd=Search&Term="Weil CF"%5BAuthor%5D&itool=EntrezSystem2.PEntrez.Pubmed.Pubmed_ResultsPanel.Pubmed_RVAbstract)**,** [**Bray CM**](http://www.ncbi.nlm.nih.gov/sites/entrez?Db=pubmed&Cmd=Search&Term="Bray CM"%5BAuthor%5D&itool=EntrezSystem2.PEntrez.Pubmed.Pubmed_ResultsPanel.Pubmed_RVAbstract)**,** [**West CE**](http://www.ncbi.nlm.nih.gov/sites/entrez?Db=pubmed&Cmd=Search&Term="West CE"%5BAuthor%5D&itool=EntrezSystem2.PEntrez.Pubmed.Pubmed_ResultsPanel.Pubmed_RVAbstract) (2007) NBS1 is involved in DNA repair and plays a synergistic role with ATM in mediating meiotic homologous recombination in plants. [Plant J.](javascript:AL_get(this, 'jour', 'Plant J.');) **52(1):**41-52.

79.[**Bagherieh-Najjar MB**](http://www.ncbi.nlm.nih.gov/sites/entrez?Db=pubmed&Cmd=Search&Term="Bagherieh-Najjar MB"%5BAuthor%5D&itool=EntrezSystem2.PEntrez.Pubmed.Pubmed_ResultsPanel.Pubmed_RVAbstract)**,** [**de Vries OM**](http://www.ncbi.nlm.nih.gov/sites/entrez?Db=pubmed&Cmd=Search&Term="de Vries OM"%5BAuthor%5D&itool=EntrezSystem2.PEntrez.Pubmed.Pubmed_ResultsPanel.Pubmed_RVAbstract)**,** [**Hille J**](http://www.ncbi.nlm.nih.gov/sites/entrez?Db=pubmed&Cmd=Search&Term="Hille J"%5BAuthor%5D&itool=EntrezSystem2.PEntrez.Pubmed.Pubmed_ResultsPanel.Pubmed_RVAbstract)**,** [**Dijkwel PP**](http://www.ncbi.nlm.nih.gov/sites/entrez?Db=pubmed&Cmd=Search&Term="Dijkwel PP"%5BAuthor%5D&itool=EntrezSystem2.PEntrez.Pubmed.Pubmed_ResultsPanel.Pubmed_RVAbstract) (2005) Arabidopsis RecQI4A suppresses homologous recombination and modulates DNA damage responses. [Plant J.](javascript:AL_get(this, 'jour', 'Plant J.');) **43(6):**789-798.

80. **Klimyuk VI, Jones JDG** (1997) AtDMC1, the Arabidopsis homologue of the yeast DMC1 gene: characterization, transposon-induced allelic variation and meiosis- associated expression. Plant J. **11**:1-14.

81. [**Reidt W**](http://www.ncbi.nlm.nih.gov/sites/entrez?Db=pubmed&Cmd=Search&Term="Reidt W"%5BAuthor%5D&itool=EntrezSystem2.PEntrez.Pubmed.Pubmed_ResultsPanel.Pubmed_RVAbstract)**,** [**Wurz R**](http://www.ncbi.nlm.nih.gov/sites/entrez?Db=pubmed&Cmd=Search&Term="Wurz R"%5BAuthor%5D&itool=EntrezSystem2.PEntrez.Pubmed.Pubmed_ResultsPanel.Pubmed_RVAbstract)**,** [**Wanieck K**](http://www.ncbi.nlm.nih.gov/sites/entrez?Db=pubmed&Cmd=Search&Term="Wanieck K"%5BAuthor%5D&itool=EntrezSystem2.PEntrez.Pubmed.Pubmed_ResultsPanel.Pubmed_RVAbstract)**,** [**Chu HH**](http://www.ncbi.nlm.nih.gov/sites/entrez?Db=pubmed&Cmd=Search&Term="Chu HH"%5BAuthor%5D&itool=EntrezSystem2.PEntrez.Pubmed.Pubmed_ResultsPanel.Pubmed_RVAbstract)**,** [**Puchta H**](http://www.ncbi.nlm.nih.gov/sites/entrez?Db=pubmed&Cmd=Search&Term="Puchta H"%5BAuthor%5D&itool=EntrezSystem2.PEntrez.Pubmed.Pubmed_ResultsPanel.Pubmed_RVAbstract) (2006) A homologue of the breast cancer-associated gene BARD1 is involved in DNA repair in plants. [EMBO J.](javascript:AL_get(this, 'jour', 'EMBO J.');) **5(18):**4326-4337.

82.[**Edmondson AC**](http://www.ncbi.nlm.nih.gov/sites/entrez?Db=pubmed&Cmd=Search&Term="Edmondson AC"%5BAuthor%5D&itool=EntrezSystem2.PEntrez.Pubmed.Pubmed_ResultsPanel.Pubmed_RVAbstract)**,** [**Song D**](http://www.ncbi.nlm.nih.gov/sites/entrez?Db=pubmed&Cmd=Search&Term="Song D"%5BAuthor%5D&itool=EntrezSystem2.PEntrez.Pubmed.Pubmed_ResultsPanel.Pubmed_RVAbstract)**,** [**Alvarez LA**](http://www.ncbi.nlm.nih.gov/sites/entrez?Db=pubmed&Cmd=Search&Term="Alvarez LA"%5BAuthor%5D&itool=EntrezSystem2.PEntrez.Pubmed.Pubmed_ResultsPanel.Pubmed_RVAbstract)**,** [**Wall MK**](http://www.ncbi.nlm.nih.gov/sites/entrez?Db=pubmed&Cmd=Search&Term="Wall MK"%5BAuthor%5D&itool=EntrezSystem2.PEntrez.Pubmed.Pubmed_ResultsPanel.Pubmed_RVAbstract)**,** [**Almond D**](http://www.ncbi.nlm.nih.gov/sites/entrez?Db=pubmed&Cmd=Search&Term="Almond D"%5BAuthor%5D&itool=EntrezSystem2.PEntrez.Pubmed.Pubmed_ResultsPanel.Pubmed_RVAbstract)**,** [**McClellan DA**](http://www.ncbi.nlm.nih.gov/sites/entrez?Db=pubmed&Cmd=Search&Term="McClellan DA"%5BAuthor%5D&itool=EntrezSystem2.PEntrez.Pubmed.Pubmed_ResultsPanel.Pubmed_RVAbstract)**,** [**Maxwell A**](http://www.ncbi.nlm.nih.gov/sites/entrez?Db=pubmed&Cmd=Search&Term="Maxwell A"%5BAuthor%5D&itool=EntrezSystem2.PEntrez.Pubmed.Pubmed_ResultsPanel.Pubmed_RVAbstract)**,** [**Nielsen BL**](http://www.ncbi.nlm.nih.gov/sites/entrez?Db=pubmed&Cmd=Search&Term="Nielsen BL"%5BAuthor%5D&itool=EntrezSystem2.PEntrez.Pubmed.Pubmed_ResultsPanel.Pubmed_RVAbstract) (2005) Characterization of a mitochondrially targeted single-stranded DNA-binding protein in Arabidopsis thaliana. [Mol Genet Genomics.](javascript:AL_get(this, 'jour', 'Mol Genet Genomics.');) **273(2):**115-122.

83. **Binet MN, Osman M, Jagendorf AT** (1993) Genomic nucleotide sequence of a gene from Arabidopsis thaliana encoding a protein homolog of Escherichia coli RecA. Plant Physiol. **103**:673-674

84. **Cerutti HD, Osman M, Grandoni P, Jagendorf AT** (1992) A homolog of Escherichia coli RecA protein in plastids of higher plants. Proc. Natl. Acad. Sci. U.S.A. **89**:8068-8072.

85. **Khazi FR, Edmondson AC, Nielsen BL** (2003) An Arabidopsis homologue of bacterial RecA that complements an E. coli recA deletion is targeted to plant mitochondria. Mol. Genet. Genomics **269**:454-463.

86. **Bleuyard J-Y, White CI** (2004) The Arabidopsis homologue of Xrcc3 plays an essential role in meiosis. EMBO J. **23**:439-449.

87.[**Panoli AP**](http://www.ncbi.nlm.nih.gov/sites/entrez?Db=pubmed&Cmd=Search&Term="Panoli AP"%5BAuthor%5D&itool=EntrezSystem2.PEntrez.Pubmed.Pubmed_ResultsPanel.Pubmed_RVAbstract)**,** [**Ravi M**](http://www.ncbi.nlm.nih.gov/sites/entrez?Db=pubmed&Cmd=Search&Term="Ravi M"%5BAuthor%5D&itool=EntrezSystem2.PEntrez.Pubmed.Pubmed_ResultsPanel.Pubmed_RVAbstract)**,** [**Sebastian J**](http://www.ncbi.nlm.nih.gov/sites/entrez?Db=pubmed&Cmd=Search&Term="Sebastian J"%5BAuthor%5D&itool=EntrezSystem2.PEntrez.Pubmed.Pubmed_ResultsPanel.Pubmed_RVAbstract)**,** [**Nishal B**](http://www.ncbi.nlm.nih.gov/sites/entrez?Db=pubmed&Cmd=Search&Term="Nishal B"%5BAuthor%5D&itool=EntrezSystem2.PEntrez.Pubmed.Pubmed_ResultsPanel.Pubmed_RVAbstract)**,** [**Reddy TV**](http://www.ncbi.nlm.nih.gov/sites/entrez?Db=pubmed&Cmd=Search&Term="Reddy TV"%5BAuthor%5D&itool=EntrezSystem2.PEntrez.Pubmed.Pubmed_ResultsPanel.Pubmed_RVAbstract)**,** [**Marimuthu MP**](http://www.ncbi.nlm.nih.gov/sites/entrez?Db=pubmed&Cmd=Search&Term="Marimuthu MP"%5BAuthor%5D&itool=EntrezSystem2.PEntrez.Pubmed.Pubmed_ResultsPanel.Pubmed_RVAbstract)**,** [**Subbiah V**](http://www.ncbi.nlm.nih.gov/sites/entrez?Db=pubmed&Cmd=Search&Term="Subbiah V"%5BAuthor%5D&itool=EntrezSystem2.PEntrez.Pubmed.Pubmed_ResultsPanel.Pubmed_RVAbstract)**,** [**Vijaybhaskar V**](http://www.ncbi.nlm.nih.gov/sites/entrez?Db=pubmed&Cmd=Search&Term="Vijaybhaskar V"%5BAuthor%5D&itool=EntrezSystem2.PEntrez.Pubmed.Pubmed_ResultsPanel.Pubmed_RVAbstract)**,** [**Siddiqi I**](http://www.ncbi.nlm.nih.gov/sites/entrez?Db=pubmed&Cmd=Search&Term="Siddiqi I"%5BAuthor%5D&itool=EntrezSystem2.PEntrez.Pubmed.Pubmed_ResultsPanel.Pubmed_RVAbstract) (2006) AtMND1 is required for homologous pairing during meiosis in Arabidopsis.[BMC Mol Biol.](javascript:AL_get(this, 'jour', 'BMC Mol Biol.');) 27:7-24.

88. **Domenichini S, Raynaud C, Ni DA, Henry Y, Bergounioux C** (2006) Atmnd1-delta1 is sensitive to gamma-irradiation and defective in meiotic DNA repair. DNA Repair (Amst) **5(4)**:455-464.

89.**Kerzendorfer C, Vignard J, Pedrosa-Harand A, Siwiec T, Akimcheva S, Jolivet S, Sablowski R, Armstrong S, Schweizer D, Mercier R, Schlögelhofer P** (2006) The Arabidopsis thaliana MND1 homologue plays a key role in meiotic homologous pairing, synapsis and recombination. J Cell Sci. **119**(Pt 12):2486-2496.

90.[**Mimida N**](http://www.ncbi.nlm.nih.gov/sites/entrez?Db=pubmed&Cmd=Search&Term="Mimida N"%5BAuthor%5D&itool=EntrezSystem2.PEntrez.Pubmed.Pubmed_ResultsPanel.Pubmed_DiscoveryPanel.Pubmed_RVAbstractPlus)**,** [**Kitamoto H**](http://www.ncbi.nlm.nih.gov/sites/entrez?Db=pubmed&Cmd=Search&Term="Kitamoto H"%5BAuthor%5D&itool=EntrezSystem2.PEntrez.Pubmed.Pubmed_ResultsPanel.Pubmed_DiscoveryPanel.Pubmed_RVAbstractPlus)**,** [**Osakabe K**](http://www.ncbi.nlm.nih.gov/sites/entrez?Db=pubmed&Cmd=Search&Term="Osakabe K"%5BAuthor%5D&itool=EntrezSystem2.PEntrez.Pubmed.Pubmed_ResultsPanel.Pubmed_DiscoveryPanel.Pubmed_RVAbstractPlus)**,** [**Nakashima M**](http://www.ncbi.nlm.nih.gov/sites/entrez?Db=pubmed&Cmd=Search&Term="Nakashima M"%5BAuthor%5D&itool=EntrezSystem2.PEntrez.Pubmed.Pubmed_ResultsPanel.Pubmed_DiscoveryPanel.Pubmed_RVAbstractPlus)**,** [**Ito Y**](http://www.ncbi.nlm.nih.gov/sites/entrez?Db=pubmed&Cmd=Search&Term="Ito Y"%5BAuthor%5D&itool=EntrezSystem2.PEntrez.Pubmed.Pubmed_ResultsPanel.Pubmed_DiscoveryPanel.Pubmed_RVAbstractPlus)**,** [**Heyer WD**](http://www.ncbi.nlm.nih.gov/sites/entrez?Db=pubmed&Cmd=Search&Term="Heyer WD"%5BAuthor%5D&itool=EntrezSystem2.PEntrez.Pubmed.Pubmed_ResultsPanel.Pubmed_DiscoveryPanel.Pubmed_RVAbstractPlus)**,** [**Toki S**](http://www.ncbi.nlm.nih.gov/sites/entrez?Db=pubmed&Cmd=Search&Term="Toki S"%5BAuthor%5D&itool=EntrezSystem2.PEntrez.Pubmed.Pubmed_ResultsPanel.Pubmed_DiscoveryPanel.Pubmed_RVAbstractPlus)**,** [**Ichikawa H**](http://www.ncbi.nlm.nih.gov/sites/entrez?Db=pubmed&Cmd=Search&Term="Ichikawa H"%5BAuthor%5D&itool=EntrezSystem2.PEntrez.Pubmed.Pubmed_ResultsPanel.Pubmed_DiscoveryPanel.Pubmed_RVAbstractPlus) (2007) Two alternatively spliced transcripts generated from OsMUS81, a rice homolog of yeast MUS81, are up-regulated by DNA-damaging treatments. [Plant Cell Physiol.](javascript:AL_get(this, 'jour', 'Plant Cell Physiol.');) **48(4):**648-654.

91. **Mengiste T, Revenkova E, Bechtold N, Paszkowski J** (1999) An SMC-like protein is required for efficient homologous recombination in Arabidopsis. EMBO J.**18 (16):**4505-4512.

92. **Hanin M, Mengiste T, Bogucki A, Paszkowski J** (2000) Elevated levels of intrachromosomal homologous recombination in Arabidopsis overexpressing the MIM gene. Plant J. **24(2)**:183-189.

93. **Dion E, Li L, Jean M, Belzile F** (2007) An Arabidopsis MLH1 mutant exhibits reproductive defects and reveals a dual role for this gene in mitotic recombination. Plant J. **51(3):**431-440.

94.**Jean M, Pelletier J, Hilpert M, Belzile F, Kunze R** (1999) Isolation and characterization of AtMLH1, a MutL homologue from Arabidopsis thaliana. Mol Gen Genet. **262(4-5):**633-642.

95. [**Adé J**](http://www.ncbi.nlm.nih.gov/sites/entrez?Db=pubmed&Cmd=Search&Term="Adé J"%5BAuthor%5D&itool=EntrezSystem2.PEntrez.Pubmed.Pubmed_ResultsPanel.Pubmed_DiscoveryPanel.Pubmed_RVAbstractPlus)**,** [**Haffani Y**](http://www.ncbi.nlm.nih.gov/sites/entrez?Db=pubmed&Cmd=Search&Term="Haffani Y"%5BAuthor%5D&itool=EntrezSystem2.PEntrez.Pubmed.Pubmed_ResultsPanel.Pubmed_DiscoveryPanel.Pubmed_RVAbstractPlus)**,** [**Beizile FJ**](http://www.ncbi.nlm.nih.gov/sites/entrez?Db=pubmed&Cmd=Search&Term="Beizile FJ"%5BAuthor%5D&itool=EntrezSystem2.PEntrez.Pubmed.Pubmed_ResultsPanel.Pubmed_DiscoveryPanel.Pubmed_RVAbstractPlus) (2001) Functional analysis of the Arabidopsis thaliana mismatch repair gene MSH2. [Genome.](javascript:AL_get(this, 'jour', 'Genome.');) **44(4)**:651-7.

96.[**Jackson N**](http://www.ncbi.nlm.nih.gov/sites/entrez?Db=pubmed&Cmd=Search&Term="Jackson N"%5BAuthor%5D&itool=EntrezSystem2.PEntrez.Pubmed.Pubmed_ResultsPanel.Pubmed_RVAbstract)**,** [**Sanchez-Moran E**](http://www.ncbi.nlm.nih.gov/sites/entrez?Db=pubmed&Cmd=Search&Term="Sanchez-Moran E"%5BAuthor%5D&itool=EntrezSystem2.PEntrez.Pubmed.Pubmed_ResultsPanel.Pubmed_RVAbstract)**,** [**Buckling E**](http://www.ncbi.nlm.nih.gov/sites/entrez?Db=pubmed&Cmd=Search&Term="Buckling E"%5BAuthor%5D&itool=EntrezSystem2.PEntrez.Pubmed.Pubmed_ResultsPanel.Pubmed_RVAbstract)**,** [**Armstrong SJ**](http://www.ncbi.nlm.nih.gov/sites/entrez?Db=pubmed&Cmd=Search&Term="Armstrong SJ"%5BAuthor%5D&itool=EntrezSystem2.PEntrez.Pubmed.Pubmed_ResultsPanel.Pubmed_RVAbstract)**,** [**Jones GH**](http://www.ncbi.nlm.nih.gov/sites/entrez?Db=pubmed&Cmd=Search&Term="Jones GH"%5BAuthor%5D&itool=EntrezSystem2.PEntrez.Pubmed.Pubmed_ResultsPanel.Pubmed_RVAbstract)**,** [**Franklin FC**](http://www.ncbi.nlm.nih.gov/sites/entrez?Db=pubmed&Cmd=Search&Term="Franklin FC"%5BAuthor%5D&itool=EntrezSystem2.PEntrez.Pubmed.Pubmed_ResultsPanel.Pubmed_RVAbstract) (2006 ) Reduced meiotic crossovers and delayed prophase I progression in AtMLH3-deficient Arabidopsis. [EMBO J.](javascript:AL_get(this, 'jour', 'EMBO J.');) **25(6):**1315-1323

97.**Emmanuel E, Yehuda E, Melamed-Bessudo C, Avivi-Ragolsky N, Levy AA** (2006)The role of AtMSH2 in homologous recombination in Arabidopsis thaliana. EMBO Rep. **7(1):**100-105.

98. **Culligan KM, Hays JB** (1997) DNA mismatch repair in plants. An Arabidopsis thaliana gene that predicts a protein belonging to the MSH2 subfamily of eukaryotic MutS homologs. Plant Physiol **115**:833-839.

99. **Ade J, Belzile F, Philippe H, Doutriaux MP** (1999) Four mismatch repair paralogues coexist in Arabidopsis thaliana: AtMSH2, AtMSH3, AtMSH6-1 and AtMSH6-2. Mol. Gen. Genet. **262**:239-249.

100.[**Lafleuriel J**](http://www.ncbi.nlm.nih.gov/sites/entrez?Db=pubmed&Cmd=Search&Term="Lafleuriel J"%5BAuthor%5D&itool=EntrezSystem2.PEntrez.Pubmed.Pubmed_ResultsPanel.Pubmed_DiscoveryPanel.Pubmed_RVAbstractPlus)**,** [**Degroote F**](http://www.ncbi.nlm.nih.gov/sites/entrez?Db=pubmed&Cmd=Search&Term="Degroote F"%5BAuthor%5D&itool=EntrezSystem2.PEntrez.Pubmed.Pubmed_ResultsPanel.Pubmed_DiscoveryPanel.Pubmed_RVAbstractPlus)**,** [**Depeiges A**](http://www.ncbi.nlm.nih.gov/sites/entrez?Db=pubmed&Cmd=Search&Term="Depeiges A"%5BAuthor%5D&itool=EntrezSystem2.PEntrez.Pubmed.Pubmed_ResultsPanel.Pubmed_DiscoveryPanel.Pubmed_RVAbstractPlus)**,** [**Picard G**](http://www.ncbi.nlm.nih.gov/sites/entrez?Db=pubmed&Cmd=Search&Term="Picard G"%5BAuthor%5D&itool=EntrezSystem2.PEntrez.Pubmed.Pubmed_ResultsPanel.Pubmed_DiscoveryPanel.Pubmed_RVAbstractPlus) (2007) Impact of the loss of AtMSH2 on double-strand break-induced recombination between highly diverged homeologous sequences in Arabidopsis thaliana germinal tissues. [Plant Mol Biol.](javascript:AL_get(this, 'jour', 'Plant Mol Biol.');) **63(6):**833-846.

101. **Culligan KM, Hays JB** (2000) **Arabidopsis** **MutS** Homologs—**A**tMSH2, **AtMSH3**, **AtMSH6**, **and** **a** **Novel** **A**tMSH7—**For**m **Three** **Distinct** **Protein** **Heterodimers** **with** **Different** **Specificities** **for** **Mismatched** DN**A** . Pl**a**nt Cell**12**: 991-1002.

102. **Higgins JD, Vignard J, Mercier R, Pugh AG, Franklin FC, Jones GH** (2008) AtMSH5 partners AtMSH4 in the class I meiotic crossover pathway in Arabidopsis thaliana, but is not required for synapsis. Plant J. **55(1)**:28-39.

103.**Wu SY, Culligan K, Lamers M, Hays J** (2003) Dissimilar mispair-recognition spectra of Arabidopsis DNA-mismatch-repair proteins MSH2*MSH6 (MutSalpha) and MSH2*MSH7 (MutSgamma). Nucleic Acids Res.**31 (20):**6027-6034.

104. [**Lu X**](http://www.ncbi.nlm.nih.gov/sites/entrez?Db=pubmed&Cmd=Search&Term="Lu X"%5BAuthor%5D&itool=EntrezSystem2.PEntrez.Pubmed.Pubmed_ResultsPanel.Pubmed_DiscoveryPanel.Pubmed_RVAbstractPlus)**,** [**Liu X**](http://www.ncbi.nlm.nih.gov/sites/entrez?Db=pubmed&Cmd=Search&Term="Liu X"%5BAuthor%5D&itool=EntrezSystem2.PEntrez.Pubmed.Pubmed_ResultsPanel.Pubmed_DiscoveryPanel.Pubmed_RVAbstractPlus)**,** [**An L**](http://www.ncbi.nlm.nih.gov/sites/entrez?Db=pubmed&Cmd=Search&Term="An L"%5BAuthor%5D&itool=EntrezSystem2.PEntrez.Pubmed.Pubmed_ResultsPanel.Pubmed_DiscoveryPanel.Pubmed_RVAbstractPlus)**,** [**Zhang W**](http://www.ncbi.nlm.nih.gov/sites/entrez?Db=pubmed&Cmd=Search&Term="Zhang W"%5BAuthor%5D&itool=EntrezSystem2.PEntrez.Pubmed.Pubmed_ResultsPanel.Pubmed_DiscoveryPanel.Pubmed_RVAbstractPlus)**,** [**Sun J**](http://www.ncbi.nlm.nih.gov/sites/entrez?Db=pubmed&Cmd=Search&Term="Sun J"%5BAuthor%5D&itool=EntrezSystem2.PEntrez.Pubmed.Pubmed_ResultsPanel.Pubmed_DiscoveryPanel.Pubmed_RVAbstractPlus)**,** [**Pei H**](http://www.ncbi.nlm.nih.gov/sites/entrez?Db=pubmed&Cmd=Search&Term="Pei H"%5BAuthor%5D&itool=EntrezSystem2.PEntrez.Pubmed.Pubmed_ResultsPanel.Pubmed_DiscoveryPanel.Pubmed_RVAbstractPlus)**,** [**Meng H**](http://www.ncbi.nlm.nih.gov/sites/entrez?Db=pubmed&Cmd=Search&Term="Meng H"%5BAuthor%5D&itool=EntrezSystem2.PEntrez.Pubmed.Pubmed_ResultsPanel.Pubmed_DiscoveryPanel.Pubmed_RVAbstractPlus)**,** [**Fan Y**](http://www.ncbi.nlm.nih.gov/sites/entrez?Db=pubmed&Cmd=Search&Term="Fan Y"%5BAuthor%5D&itool=EntrezSystem2.PEntrez.Pubmed.Pubmed_ResultsPanel.Pubmed_DiscoveryPanel.Pubmed_RVAbstractPlus)**,** [**Zhang C**](http://www.ncbi.nlm.nih.gov/sites/entrez?Db=pubmed&Cmd=Search&Term="Zhang C"%5BAuthor%5D&itool=EntrezSystem2.PEntrez.Pubmed.Pubmed_ResultsPanel.Pubmed_DiscoveryPanel.Pubmed_RVAbstractPlus) (2008) The Arabidopsis MutS homolog AtMSH5 is required for normal meiosis. [Cell Res.](javascript:AL_get(this, 'jour', 'Cell Res.');) **18(5):**589-599.

105.[**Li L**](http://www.ncbi.nlm.nih.gov/sites/entrez?Db=pubmed&Cmd=Search&Term="Li L"%5BAuthor%5D&itool=EntrezSystem2.PEntrez.Pubmed.Pubmed_ResultsPanel.Pubmed_RVAbstract)**,** [**Dion E**](http://www.ncbi.nlm.nih.gov/sites/entrez?Db=pubmed&Cmd=Search&Term="Dion E"%5BAuthor%5D&itool=EntrezSystem2.PEntrez.Pubmed.Pubmed_ResultsPanel.Pubmed_RVAbstract)**,** [**Richard G**](http://www.ncbi.nlm.nih.gov/sites/entrez?Db=pubmed&Cmd=Search&Term="Richard G"%5BAuthor%5D&itool=EntrezSystem2.PEntrez.Pubmed.Pubmed_ResultsPanel.Pubmed_RVAbstract)**,** [**Domingue O**](http://www.ncbi.nlm.nih.gov/sites/entrez?Db=pubmed&Cmd=Search&Term="Domingue O"%5BAuthor%5D&itool=EntrezSystem2.PEntrez.Pubmed.Pubmed_ResultsPanel.Pubmed_RVAbstract)**,** [**Jean M**](http://www.ncbi.nlm.nih.gov/sites/entrez?Db=pubmed&Cmd=Search&Term="Jean M"%5BAuthor%5D&itool=EntrezSystem2.PEntrez.Pubmed.Pubmed_ResultsPanel.Pubmed_RVAbstract)**,** [**Belzile FJ**](http://www.ncbi.nlm.nih.gov/sites/entrez?Db=pubmed&Cmd=Search&Term="Belzile FJ"%5BAuthor%5D&itool=EntrezSystem2.PEntrez.Pubmed.Pubmed_ResultsPanel.Pubmed_RVAbstract) (2009)The Arabidopsis DNA mismatch repair gene PMS1 restricts somatic recombination between homeologous sequences. [Plant Mol Biol.](javascript:AL_get(this, 'jour', 'Plant Mol Biol.');) **69(6):**675-84.

106.[**Alou AH**](http://www.ncbi.nlm.nih.gov/sites/entrez?Db=pubmed&Cmd=Search&Term="Alou AH"%5BAuthor%5D&itool=EntrezSystem2.PEntrez.Pubmed.Pubmed_ResultsPanel.Pubmed_RVAbstract)**,** [**Azaiez A**](http://www.ncbi.nlm.nih.gov/sites/entrez?Db=pubmed&Cmd=Search&Term="Azaiez A"%5BAuthor%5D&itool=EntrezSystem2.PEntrez.Pubmed.Pubmed_ResultsPanel.Pubmed_RVAbstract)**,** [**Jean M**](http://www.ncbi.nlm.nih.gov/sites/entrez?Db=pubmed&Cmd=Search&Term="Jean M"%5BAuthor%5D&itool=EntrezSystem2.PEntrez.Pubmed.Pubmed_ResultsPanel.Pubmed_RVAbstract)**,** [**Belzile FJ**](http://www.ncbi.nlm.nih.gov/sites/entrez?Db=pubmed&Cmd=Search&Term="Belzile FJ"%5BAuthor%5D&itool=EntrezSystem2.PEntrez.Pubmed.Pubmed_ResultsPanel.Pubmed_RVAbstract) (2004) Involvement of the Arabidopsis thaliana AtPMS1 gene in somatic repeat instability. [Plant Mol Biol.](javascript:AL_get(this, 'jour', 'Plant Mol Biol.');) **56(3):**339-349.

107. **Tamura K, Adachi Y, Chiba K, Oguchi K, Takahashi H** (2002) Identification of Ku70 and Ku80 homologues in Arabidopsis thaliana: evidence for a role in the repair of DNA double-strand breaks Plant Journal **29**: 771-781.

108. **Liu PF, Wang YK, Chang WC, Chang HY, Pan RL** (2008).Regulation of Arabidopsis thaliana Ku genes at different developmental stages under heat stress. Biochim Biophys Acta.**1779 (6-7):**402-407.

109. **Bundock P, van Attikum H, Hooykaas P** (2002). Increased telomere length and hypersensitivity to DNA damaging agents in an Arabidopsis KU70 mutant. Nucleic Acids Res **30**: 3395-3400.

110. **Li J, Vaidya M, White C, Vainstein A, Citovsky V, Tzfira T** (2005) Involvement of KU80 in T-DNA integration in plant cells. Proc Natl Acad Sci U S A. **102(52)**:19231-19236.

111.[**West CE**](http://www.ncbi.nlm.nih.gov/sites/entrez?Db=pubmed&Cmd=Search&Term="West CE"%5BAuthor%5D&itool=EntrezSystem2.PEntrez.Pubmed.Pubmed_ResultsPanel.Pubmed_DiscoveryPanel.Pubmed_RVAbstractPlus)**,** [**Waterworth WM**](http://www.ncbi.nlm.nih.gov/sites/entrez?Db=pubmed&Cmd=Search&Term="Waterworth WM"%5BAuthor%5D&itool=EntrezSystem2.PEntrez.Pubmed.Pubmed_ResultsPanel.Pubmed_DiscoveryPanel.Pubmed_RVAbstractPlus)**,** [**Story GW**](http://www.ncbi.nlm.nih.gov/sites/entrez?Db=pubmed&Cmd=Search&Term="Story GW"%5BAuthor%5D&itool=EntrezSystem2.PEntrez.Pubmed.Pubmed_ResultsPanel.Pubmed_DiscoveryPanel.Pubmed_RVAbstractPlus)**,** [**Sunderland PA**](http://www.ncbi.nlm.nih.gov/sites/entrez?Db=pubmed&Cmd=Search&Term="Sunderland PA"%5BAuthor%5D&itool=EntrezSystem2.PEntrez.Pubmed.Pubmed_ResultsPanel.Pubmed_DiscoveryPanel.Pubmed_RVAbstractPlus)**,** [**Jiang Q**](http://www.ncbi.nlm.nih.gov/sites/entrez?Db=pubmed&Cmd=Search&Term="Jiang Q"%5BAuthor%5D&itool=EntrezSystem2.PEntrez.Pubmed.Pubmed_ResultsPanel.Pubmed_DiscoveryPanel.Pubmed_RVAbstractPlus)**,** [**Bray CM**](http://www.ncbi.nlm.nih.gov/sites/entrez?Db=pubmed&Cmd=Search&Term="Bray CM"%5BAuthor%5D&itool=EntrezSystem2.PEntrez.Pubmed.Pubmed_ResultsPanel.Pubmed_DiscoveryPanel.Pubmed_RVAbstractPlus) (2002) Disruption of the Arabidopsis AtKu80 gene demonstrates an essential role for AtKu80 protein in efficient repair of DNA double-strand breaks in vivo. [Plant J.](javascript:AL_get(this, 'jour', 'Plant J.');) 31(4):517-528.

112. [**Li B**](http://www.ncbi.nlm.nih.gov/sites/entrez?Db=pubmed&Cmd=Search&Term="Li B"%5BAuthor%5D&itool=EntrezSystem2.PEntrez.Pubmed.Pubmed_ResultsPanel.Pubmed_RVAbstract)**,** [**Conway N**](http://www.ncbi.nlm.nih.gov/sites/entrez?Db=pubmed&Cmd=Search&Term="Conway N"%5BAuthor%5D&itool=EntrezSystem2.PEntrez.Pubmed.Pubmed_ResultsPanel.Pubmed_RVAbstract)**,** [**Navarro S**](http://www.ncbi.nlm.nih.gov/sites/entrez?Db=pubmed&Cmd=Search&Term="Navarro S"%5BAuthor%5D&itool=EntrezSystem2.PEntrez.Pubmed.Pubmed_ResultsPanel.Pubmed_RVAbstract)**,** [**Comai L**](http://www.ncbi.nlm.nih.gov/sites/entrez?Db=pubmed&Cmd=Search&Term="Comai L"%5BAuthor%5D&itool=EntrezSystem2.PEntrez.Pubmed.Pubmed_ResultsPanel.Pubmed_RVAbstract)**,** [**Comai L**](http://www.ncbi.nlm.nih.gov/sites/entrez?Db=pubmed&Cmd=Search&Term="Comai L"%5BAuthor%5D&itool=EntrezSystem2.PEntrez.Pubmed.Pubmed_ResultsPanel.Pubmed_RVAbstract) (2005)A conserved and species-specific functional interaction between the Werner syndrome-like exonuclease at WEX and the Ku heterodimer in Arabidopsis. [Nucleic Acids Res.](javascript:AL_get(this, 'jour', 'Nucleic Acids Res.');) **33(21):**6861-6867.

113.[**Gallego ME**](http://www.ncbi.nlm.nih.gov/sites/entrez?Db=pubmed&Cmd=Search&Term="Gallego ME"%5BAuthor%5D&itool=EntrezSystem2.PEntrez.Pubmed.Pubmed_ResultsPanel.Pubmed_RVAbstract)**,** [**Bleuyard JY**](http://www.ncbi.nlm.nih.gov/sites/entrez?Db=pubmed&Cmd=Search&Term="Bleuyard JY"%5BAuthor%5D&itool=EntrezSystem2.PEntrez.Pubmed.Pubmed_ResultsPanel.Pubmed_RVAbstract)**,** [**Daoudal-Cotterell S**](http://www.ncbi.nlm.nih.gov/sites/entrez?Db=pubmed&Cmd=Search&Term="Daoudal-Cotterell S"%5BAuthor%5D&itool=EntrezSystem2.PEntrez.Pubmed.Pubmed_ResultsPanel.Pubmed_RVAbstract)**,** [**Jallut N**](http://www.ncbi.nlm.nih.gov/sites/entrez?Db=pubmed&Cmd=Search&Term="Jallut N"%5BAuthor%5D&itool=EntrezSystem2.PEntrez.Pubmed.Pubmed_ResultsPanel.Pubmed_RVAbstract)**,** [**White CI**](http://www.ncbi.nlm.nih.gov/sites/entrez?Db=pubmed&Cmd=Search&Term="White CI"%5BAuthor%5D&itool=EntrezSystem2.PEntrez.Pubmed.Pubmed_ResultsPanel.Pubmed_RVAbstract) (2003) Ku80 plays a role in non-homologous recombination but is not required for T-DNA integration in Arabidopsis. [Plant J.](javascript:AL_get(this, 'jour', 'Plant J.');) **35(5)**:557-565.

114.[**Friesner J**](http://www.ncbi.nlm.nih.gov/sites/entrez?Db=pubmed&Cmd=Search&Term="Friesner J"%5BAuthor%5D&itool=EntrezSystem2.PEntrez.Pubmed.Pubmed_ResultsPanel.Pubmed_RVAbstract)**,** [**Britt AB**](http://www.ncbi.nlm.nih.gov/sites/entrez?Db=pubmed&Cmd=Search&Term="Britt AB"%5BAuthor%5D&itool=EntrezSystem2.PEntrez.Pubmed.Pubmed_ResultsPanel.Pubmed_RVAbstract) (2003) Ku80- and DNA ligase IV-deficient plants are sensitive to ionizing radiation and defective in T-DNA integration. [Plant J.](javascript:AL_get(this, 'jour', 'Plant J.');) **34(4):**427-440.

115. **Zellinger B, Akimcheva S, Puizina J, Schirato M, Riha K** (2007) Ku suppresses formation of telomeric circles and alternative telomere lengthening in Arabidopsis. Mol Cell. **27(1):**163-169.

116.**West CE, Waterworth WM, Jiang Q, Bray CM** (2000) Arabidopsis DNA ligase IV is induced by gamma-irradiation and interacts with an Arabidopsis homologue of the double strand break repair protein XRCC4. Plant J. **24**:67-78.

117. **van Attikum H, Bundock P, Overmeer RM, Lee L-Y, Gelvin SB, Hooykaas PJJ** (2003) The Arabidopsis AtLIG4 gene is required for the repair of DNA damage, but not for the integration of Agrobacterium T-DNA. Nucleic Acids Res. **31**:4247-4255.

118. **Friesner J, Britt AB** (2003) Ku80- and DNA ligase IV-deficient plants are sensitive to ionizing radiation and defective in T-DNA integration. Plant J. **34**:427-440.

119.**da Costa-Nunes JA, Bhatt AM, O'Shea S, West CE, Bray CM, Grossniklaus U, Dickinson HG** (2006) Characterization of the three Arabidopsis thaliana RAD21 cohesins reveals differential responses to ionizing radiation. J Exp Bot. 57(4):971-983.

120.**Kozak J,** [**West CE**](http://www.ncbi.nlm.nih.gov/sites/entrez?Db=pubmed&Cmd=Search&Term="West CE"%5BAuthor%5D&itool=EntrezSystem2.PEntrez.Pubmed.Pubmed_ResultsPanel.Pubmed_DiscoveryPanel.Pubmed_RVAbstractPlus)**,** [**White C**](http://www.ncbi.nlm.nih.gov/sites/entrez?Db=pubmed&Cmd=Search&Term="White C"%5BAuthor%5D&itool=EntrezSystem2.PEntrez.Pubmed.Pubmed_ResultsPanel.Pubmed_DiscoveryPanel.Pubmed_RVAbstractPlus)**,** [**da Costa-Nunes JA**](http://www.ncbi.nlm.nih.gov/sites/entrez?Db=pubmed&Cmd=Search&Term="da Costa-Nunes JA"%5BAuthor%5D&itool=EntrezSystem2.PEntrez.Pubmed.Pubmed_ResultsPanel.Pubmed_DiscoveryPanel.Pubmed_RVAbstractPlus)**,** [**Angelis KJ**](http://www.ncbi.nlm.nih.gov/sites/entrez?Db=pubmed&Cmd=Search&Term="Angelis KJ"%5BAuthor%5D&itool=EntrezSystem2.PEntrez.Pubmed.Pubmed_ResultsPanel.Pubmed_DiscoveryPanel.Pubmed_RVAbstractPlus) (2009) Rapid repair of DNA double strand breaks in Arabidopsis thaliana is dependent on proteins involved in chromosome structure maintenance. [DNA Repair (Amst).](javascript:AL_get(this, 'jour', 'DNA Repair (Amst).');) **8(3):**413-419.

121.[**Kimura S**](http://www.ncbi.nlm.nih.gov/sites/entrez?Db=pubmed&Cmd=Search&Term="Kimura S"%5BAuthor%5D&itool=EntrezSystem2.PEntrez.Pubmed.Pubmed_ResultsPanel.Pubmed_DiscoveryPanel.Pubmed_RVAbstractPlus)**,** [**Ueda T**](http://www.ncbi.nlm.nih.gov/sites/entrez?Db=pubmed&Cmd=Search&Term="Ueda T"%5BAuthor%5D&itool=EntrezSystem2.PEntrez.Pubmed.Pubmed_ResultsPanel.Pubmed_DiscoveryPanel.Pubmed_RVAbstractPlus)**,** [**Hatanaka M**](http://www.ncbi.nlm.nih.gov/sites/entrez?Db=pubmed&Cmd=Search&Term="Hatanaka M"%5BAuthor%5D&itool=EntrezSystem2.PEntrez.Pubmed.Pubmed_ResultsPanel.Pubmed_DiscoveryPanel.Pubmed_RVAbstractPlus)**,** [**Takenouchi M**](http://www.ncbi.nlm.nih.gov/sites/entrez?Db=pubmed&Cmd=Search&Term="Takenouchi M"%5BAuthor%5D&itool=EntrezSystem2.PEntrez.Pubmed.Pubmed_ResultsPanel.Pubmed_DiscoveryPanel.Pubmed_RVAbstractPlus)**,** [**Hashimoto J**](http://www.ncbi.nlm.nih.gov/sites/entrez?Db=pubmed&Cmd=Search&Term="Hashimoto J"%5BAuthor%5D&itool=EntrezSystem2.PEntrez.Pubmed.Pubmed_ResultsPanel.Pubmed_DiscoveryPanel.Pubmed_RVAbstractPlus)**,** [**Sakaguchi K**](http://www.ncbi.nlm.nih.gov/sites/entrez?Db=pubmed&Cmd=Search&Term="Sakaguchi K"%5BAuthor%5D&itool=EntrezSystem2.PEntrez.Pubmed.Pubmed_ResultsPanel.Pubmed_DiscoveryPanel.Pubmed_RVAbstractPlus) (2000)Plant homologue of flap endonuclease-1: molecular cloning, characterization, and evidence of expression in meristematic tissues. [Plant Mol Biol.](javascript:AL_get(this, 'jour', 'Plant Mol Biol.');) **42(3):**415-427.

122.[**Furukawa T**](http://www.ncbi.nlm.nih.gov/sites/entrez?Db=pubmed&Cmd=Search&Term="Furukawa T"%5BAuthor%5D&itool=EntrezSystem2.PEntrez.Pubmed.Pubmed_ResultsPanel.Pubmed_DiscoveryPanel.Pubmed_RVAbstractPlus)**,** [**Kimura S**](http://www.ncbi.nlm.nih.gov/sites/entrez?Db=pubmed&Cmd=Search&Term="Kimura S"%5BAuthor%5D&itool=EntrezSystem2.PEntrez.Pubmed.Pubmed_ResultsPanel.Pubmed_DiscoveryPanel.Pubmed_RVAbstractPlus)**,** [**Ishibashi T**](http://www.ncbi.nlm.nih.gov/sites/entrez?Db=pubmed&Cmd=Search&Term="Ishibashi T"%5BAuthor%5D&itool=EntrezSystem2.PEntrez.Pubmed.Pubmed_ResultsPanel.Pubmed_DiscoveryPanel.Pubmed_RVAbstractPlus)**,** [**Mori Y**](http://www.ncbi.nlm.nih.gov/sites/entrez?Db=pubmed&Cmd=Search&Term="Mori Y"%5BAuthor%5D&itool=EntrezSystem2.PEntrez.Pubmed.Pubmed_ResultsPanel.Pubmed_DiscoveryPanel.Pubmed_RVAbstractPlus)**,** [**Hashimoto J**](http://www.ncbi.nlm.nih.gov/sites/entrez?Db=pubmed&Cmd=Search&Term="Hashimoto J"%5BAuthor%5D&itool=EntrezSystem2.PEntrez.Pubmed.Pubmed_ResultsPanel.Pubmed_DiscoveryPanel.Pubmed_RVAbstractPlus)**,** [**Sakaguchi K**](http://www.ncbi.nlm.nih.gov/sites/entrez?Db=pubmed&Cmd=Search&Term="Sakaguchi K"%5BAuthor%5D&itool=EntrezSystem2.PEntrez.Pubmed.Pubmed_ResultsPanel.Pubmed_DiscoveryPanel.Pubmed_RVAbstractPlus)(2003) OsSEND-1: a new RAD2 nuclease family member in higher plants. [Plant Mol Biol.](javascript:AL_get(this, 'jour', 'Plant Mol Biol.');) **51(1):**59-70.

123.[**Hartung F**](http://www.ncbi.nlm.nih.gov/sites/entrez?Db=pubmed&Cmd=Search&Term="Hartung F"%5BAuthor%5D&itool=EntrezSystem2.PEntrez.Pubmed.Pubmed_ResultsPanel.Pubmed_RVAbstract)**,** [**Wurz-Wildersinn R**](http://www.ncbi.nlm.nih.gov/sites/entrez?Db=pubmed&Cmd=Search&Term="Wurz-Wildersinn R"%5BAuthor%5D&itool=EntrezSystem2.PEntrez.Pubmed.Pubmed_ResultsPanel.Pubmed_RVAbstract)**,** [**Fuchs J**](http://www.ncbi.nlm.nih.gov/sites/entrez?Db=pubmed&Cmd=Search&Term="Fuchs J"%5BAuthor%5D&itool=EntrezSystem2.PEntrez.Pubmed.Pubmed_ResultsPanel.Pubmed_RVAbstract)**,** [**Schubert I**](http://www.ncbi.nlm.nih.gov/sites/entrez?Db=pubmed&Cmd=Search&Term="Schubert I"%5BAuthor%5D&itool=EntrezSystem2.PEntrez.Pubmed.Pubmed_ResultsPanel.Pubmed_RVAbstract)**,** [**Suer S**](http://www.ncbi.nlm.nih.gov/sites/entrez?Db=pubmed&Cmd=Search&Term="Suer S"%5BAuthor%5D&itool=EntrezSystem2.PEntrez.Pubmed.Pubmed_ResultsPanel.Pubmed_RVAbstract)**,** [**Puchta H**](http://www.ncbi.nlm.nih.gov/sites/entrez?Db=pubmed&Cmd=Search&Term="Puchta H"%5BAuthor%5D&itool=EntrezSystem2.PEntrez.Pubmed.Pubmed_ResultsPanel.Pubmed_RVAbstract) (2007) The catalytically active tyrosine residues of both SPO11-1 and SPO11-2 are required for meiotic double-strand break induction in Arabidopsis. [Plant Cell](javascript:AL_get(this, 'jour', 'Plant Cell.');) **19(10):**3090-3099.

124.[**Stacey NJ**](http://www.ncbi.nlm.nih.gov/sites/entrez?Db=pubmed&Cmd=Search&Term="Stacey NJ"%5BAuthor%5D&itool=EntrezSystem2.PEntrez.Pubmed.Pubmed_ResultsPanel.Pubmed_RVAbstract)**,** [**Kuromori T**](http://www.ncbi.nlm.nih.gov/sites/entrez?Db=pubmed&Cmd=Search&Term="Kuromori T"%5BAuthor%5D&itool=EntrezSystem2.PEntrez.Pubmed.Pubmed_ResultsPanel.Pubmed_RVAbstract)**,** [**Azumi Y**](http://www.ncbi.nlm.nih.gov/sites/entrez?Db=pubmed&Cmd=Search&Term="Azumi Y"%5BAuthor%5D&itool=EntrezSystem2.PEntrez.Pubmed.Pubmed_ResultsPanel.Pubmed_RVAbstract)**,** [**Roberts G**](http://www.ncbi.nlm.nih.gov/sites/entrez?Db=pubmed&Cmd=Search&Term="Roberts G"%5BAuthor%5D&itool=EntrezSystem2.PEntrez.Pubmed.Pubmed_ResultsPanel.Pubmed_RVAbstract)**,** [**Breuer C**](http://www.ncbi.nlm.nih.gov/sites/entrez?Db=pubmed&Cmd=Search&Term="Breuer C"%5BAuthor%5D&itool=EntrezSystem2.PEntrez.Pubmed.Pubmed_ResultsPanel.Pubmed_RVAbstract)**,** [**Wada T**](http://www.ncbi.nlm.nih.gov/sites/entrez?Db=pubmed&Cmd=Search&Term="Wada T"%5BAuthor%5D&itool=EntrezSystem2.PEntrez.Pubmed.Pubmed_ResultsPanel.Pubmed_RVAbstract)**,** [**Maxwell A**](http://www.ncbi.nlm.nih.gov/sites/entrez?Db=pubmed&Cmd=Search&Term="Maxwell A"%5BAuthor%5D&itool=EntrezSystem2.PEntrez.Pubmed.Pubmed_ResultsPanel.Pubmed_RVAbstract)**,** [**Roberts K**](http://www.ncbi.nlm.nih.gov/sites/entrez?Db=pubmed&Cmd=Search&Term="Roberts K"%5BAuthor%5D&itool=EntrezSystem2.PEntrez.Pubmed.Pubmed_ResultsPanel.Pubmed_RVAbstract)**,** [**Sugimoto-Shirasu K**](http://www.ncbi.nlm.nih.gov/sites/entrez?Db=pubmed&Cmd=Search&Term="Sugimoto-Shirasu K"%5BAuthor%5D&itool=EntrezSystem2.PEntrez.Pubmed.Pubmed_ResultsPanel.Pubmed_RVAbstract) (2006) Arabidopsis SPO11-2 functions with SPO11-1 in meiotic recombination. [Plant J.](javascript:AL_get(this, 'jour', 'Plant J.');) **48(2)**:206-216.

125.[**Grelon M**](http://www.ncbi.nlm.nih.gov/sites/entrez?Db=pubmed&Cmd=Search&Term="Grelon M"%5BAuthor%5D&itool=EntrezSystem2.PEntrez.Pubmed.Pubmed_ResultsPanel.Pubmed_RVAbstract)**,** [**Vezon D**](http://www.ncbi.nlm.nih.gov/sites/entrez?Db=pubmed&Cmd=Search&Term="Vezon D"%5BAuthor%5D&itool=EntrezSystem2.PEntrez.Pubmed.Pubmed_ResultsPanel.Pubmed_RVAbstract)**,** [**Gendrot G**](http://www.ncbi.nlm.nih.gov/sites/entrez?Db=pubmed&Cmd=Search&Term="Gendrot G"%5BAuthor%5D&itool=EntrezSystem2.PEntrez.Pubmed.Pubmed_ResultsPanel.Pubmed_RVAbstract)**,** [**Pelletier G**](http://www.ncbi.nlm.nih.gov/sites/entrez?Db=pubmed&Cmd=Search&Term="Pelletier G"%5BAuthor%5D&itool=EntrezSystem2.PEntrez.Pubmed.Pubmed_ResultsPanel.Pubmed_RVAbstract) (2001) AtSPO11-1 is necessary for efficient meiotic recombination in plants. [EMBO J.](javascript:AL_get(this, 'jour', 'EMBO J.');) **20(3)**:589-600.

126.[**Furukawa T**](http://www.ncbi.nlm.nih.gov/sites/entrez?Db=pubmed&Cmd=Search&Term="Furukawa T"%5BAuthor%5D&itool=EntrezSystem2.PEntrez.Pubmed.Pubmed_ResultsPanel.Pubmed_DiscoveryPanel.Pubmed_RVAbstractPlus)**,** [**Imamura T**](http://www.ncbi.nlm.nih.gov/sites/entrez?Db=pubmed&Cmd=Search&Term="Imamura T"%5BAuthor%5D&itool=EntrezSystem2.PEntrez.Pubmed.Pubmed_ResultsPanel.Pubmed_DiscoveryPanel.Pubmed_RVAbstractPlus)**,** [**Kitamoto HK**](http://www.ncbi.nlm.nih.gov/sites/entrez?Db=pubmed&Cmd=Search&Term="Kitamoto HK"%5BAuthor%5D&itool=EntrezSystem2.PEntrez.Pubmed.Pubmed_ResultsPanel.Pubmed_DiscoveryPanel.Pubmed_RVAbstractPlus)**,** [**Shimada H**](http://www.ncbi.nlm.nih.gov/sites/entrez?Db=pubmed&Cmd=Search&Term="Shimada H"%5BAuthor%5D&itool=EntrezSystem2.PEntrez.Pubmed.Pubmed_ResultsPanel.Pubmed_DiscoveryPanel.Pubmed_RVAbstractPlus) (2008)Rice exonuclease-1 homologue, OsEXO1, that interacts with DNA polymerase lambda and RPA subunit proteins, is involved in cell proliferation. [Plant Mol Biol.](javascript:AL_get(this, 'jour', 'Plant Mol Biol.');) **66(5):**519-531.

127.[**Wang C**](http://www.ncbi.nlm.nih.gov/sites/entrez?Db=pubmed&Cmd=Search&Term="Wang C"%5BAuthor%5D&itool=EntrezSystem2.PEntrez.Pubmed.Pubmed_ResultsPanel.Pubmed_RVAbstract)**,** [**Liu Z**](http://www.ncbi.nlm.nih.gov/sites/entrez?Db=pubmed&Cmd=Search&Term="Liu Z"%5BAuthor%5D&itool=EntrezSystem2.PEntrez.Pubmed.Pubmed_ResultsPanel.Pubmed_RVAbstract) (2006) Arabidopsis ribonucleotide reductases are critical for cell cycle progression, DNA damage repair, and plant development. [Plant Cell](javascript:AL_get(this, 'jour', 'Plant Cell.');) **18(2)**:350-365

128.[**Yoshimura K**](http://www.ncbi.nlm.nih.gov/sites/entrez?Db=pubmed&Cmd=Search&Term="Yoshimura K"%5BAuthor%5D&itool=EntrezSystem2.PEntrez.Pubmed.Pubmed_ResultsPanel.Pubmed_DiscoveryPanel.Pubmed_RVAbstractPlus)**,** [**Ogawa T**](http://www.ncbi.nlm.nih.gov/sites/entrez?Db=pubmed&Cmd=Search&Term="Ogawa T"%5BAuthor%5D&itool=EntrezSystem2.PEntrez.Pubmed.Pubmed_ResultsPanel.Pubmed_DiscoveryPanel.Pubmed_RVAbstractPlus)**,** [**Ueda Y**](http://www.ncbi.nlm.nih.gov/sites/entrez?Db=pubmed&Cmd=Search&Term="Ueda Y"%5BAuthor%5D&itool=EntrezSystem2.PEntrez.Pubmed.Pubmed_ResultsPanel.Pubmed_DiscoveryPanel.Pubmed_RVAbstractPlus)**,** [**Shigeoka S**](http://www.ncbi.nlm.nih.gov/sites/entrez?Db=pubmed&Cmd=Search&Term="Shigeoka S"%5BAuthor%5D&itool=EntrezSystem2.PEntrez.Pubmed.Pubmed_ResultsPanel.Pubmed_DiscoveryPanel.Pubmed_RVAbstractPlus) (2007)AtNUDX1, an 8-oxo-7,8-dihydro-2'-deoxyguanosine 5'-triphosphate pyrophosphohydrolase, is responsible for eliminating oxidized nucleotides in Arabidopsis. [Plant Cell Physiol.](javascript:AL_get(this, 'jour', 'Plant Cell Physiol.');) **48(10)**:1438-1449.

129.[**Anderson HJ**](http://www.ncbi.nlm.nih.gov/sites/entrez?Db=pubmed&Cmd=Search&Term="Anderson HJ"%5BAuthor%5D&itool=EntrezSystem2.PEntrez.Pubmed.Pubmed_ResultsPanel.Pubmed_RVAbstract)**,** [**Vonarx EJ**](http://www.ncbi.nlm.nih.gov/sites/entrez?Db=pubmed&Cmd=Search&Term="Vonarx EJ"%5BAuthor%5D&itool=EntrezSystem2.PEntrez.Pubmed.Pubmed_ResultsPanel.Pubmed_RVAbstract)**,** [**Pastushok L**](http://www.ncbi.nlm.nih.gov/sites/entrez?Db=pubmed&Cmd=Search&Term="Pastushok L"%5BAuthor%5D&itool=EntrezSystem2.PEntrez.Pubmed.Pubmed_ResultsPanel.Pubmed_RVAbstract)**,** [**Nakagawa M**](http://www.ncbi.nlm.nih.gov/sites/entrez?Db=pubmed&Cmd=Search&Term="Nakagawa M"%5BAuthor%5D&itool=EntrezSystem2.PEntrez.Pubmed.Pubmed_ResultsPanel.Pubmed_RVAbstract)**,** [**Katafuchi A**](http://www.ncbi.nlm.nih.gov/sites/entrez?Db=pubmed&Cmd=Search&Term="Katafuchi A"%5BAuthor%5D&itool=EntrezSystem2.PEntrez.Pubmed.Pubmed_ResultsPanel.Pubmed_RVAbstract)**,** [**Gruz P**](http://www.ncbi.nlm.nih.gov/sites/entrez?Db=pubmed&Cmd=Search&Term="Gruz P"%5BAuthor%5D&itool=EntrezSystem2.PEntrez.Pubmed.Pubmed_ResultsPanel.Pubmed_RVAbstract)**,** [**Di Rubbo A**](http://www.ncbi.nlm.nih.gov/sites/entrez?Db=pubmed&Cmd=Search&Term="Di Rubbo A"%5BAuthor%5D&itool=EntrezSystem2.PEntrez.Pubmed.Pubmed_ResultsPanel.Pubmed_RVAbstract)**,** [**Grice DM**](http://www.ncbi.nlm.nih.gov/sites/entrez?Db=pubmed&Cmd=Search&Term="Grice DM"%5BAuthor%5D&itool=EntrezSystem2.PEntrez.Pubmed.Pubmed_ResultsPanel.Pubmed_RVAbstract)**,** [**Osmond MJ**](http://www.ncbi.nlm.nih.gov/sites/entrez?Db=pubmed&Cmd=Search&Term="Osmond MJ"%5BAuthor%5D&itool=EntrezSystem2.PEntrez.Pubmed.Pubmed_ResultsPanel.Pubmed_RVAbstract)**,** [**Sakamoto AN**](http://www.ncbi.nlm.nih.gov/sites/entrez?Db=pubmed&Cmd=Search&Term="Sakamoto AN"%5BAuthor%5D&itool=EntrezSystem2.PEntrez.Pubmed.Pubmed_ResultsPanel.Pubmed_RVAbstract)**,** [**Nohmi T**](http://www.ncbi.nlm.nih.gov/sites/entrez?Db=pubmed&Cmd=Search&Term="Nohmi T"%5BAuthor%5D&itool=EntrezSystem2.PEntrez.Pubmed.Pubmed_ResultsPanel.Pubmed_RVAbstract)**,** [**Xiao W**](http://www.ncbi.nlm.nih.gov/sites/entrez?Db=pubmed&Cmd=Search&Term="Xiao W"%5BAuthor%5D&itool=EntrezSystem2.PEntrez.Pubmed.Pubmed_ResultsPanel.Pubmed_RVAbstract)**,** [**Kunz BA**](http://www.ncbi.nlm.nih.gov/sites/entrez?Db=pubmed&Cmd=Search&Term="Kunz BA"%5BAuthor%5D&itool=EntrezSystem2.PEntrez.Pubmed.Pubmed_ResultsPanel.Pubmed_RVAbstract) (2008) Arabidopsis thaliana Y-family DNA polymerase eta catalyses translesion synthesis and interacts functionally with PCNA2.[Plant J.](javascript:AL_get(this, 'jour', 'Plant J.');) **55(6):**895-908.

130.[**Takahashi S**](http://www.ncbi.nlm.nih.gov/sites/entrez?Db=pubmed&Cmd=Search&Term="Takahashi S"%5BAuthor%5D&itool=EntrezSystem2.PEntrez.Pubmed.Pubmed_ResultsPanel.Pubmed_RVAbstract)**,** [**Sakamoto A**](http://www.ncbi.nlm.nih.gov/sites/entrez?Db=pubmed&Cmd=Search&Term="Sakamoto A"%5BAuthor%5D&itool=EntrezSystem2.PEntrez.Pubmed.Pubmed_ResultsPanel.Pubmed_RVAbstract)**,** [**Sato S**](http://www.ncbi.nlm.nih.gov/sites/entrez?Db=pubmed&Cmd=Search&Term="Sato S"%5BAuthor%5D&itool=EntrezSystem2.PEntrez.Pubmed.Pubmed_ResultsPanel.Pubmed_RVAbstract)**,** [**Kato T**](http://www.ncbi.nlm.nih.gov/sites/entrez?Db=pubmed&Cmd=Search&Term="Kato T"%5BAuthor%5D&itool=EntrezSystem2.PEntrez.Pubmed.Pubmed_ResultsPanel.Pubmed_RVAbstract)**,** [**Tabata S**](http://www.ncbi.nlm.nih.gov/sites/entrez?Db=pubmed&Cmd=Search&Term="Tabata S"%5BAuthor%5D&itool=EntrezSystem2.PEntrez.Pubmed.Pubmed_ResultsPanel.Pubmed_RVAbstract)**,** [**Tanaka A**](http://www.ncbi.nlm.nih.gov/sites/entrez?Db=pubmed&Cmd=Search&Term="Tanaka A"%5BAuthor%5D&itool=EntrezSystem2.PEntrez.Pubmed.Pubmed_ResultsPanel.Pubmed_RVAbstract) (2005) Roles of Arabidopsis AtREV1 and AtREV7 in translesion synthesis. [Plant Physiol.](javascript:AL_get(this, 'jour', 'Plant Physiol.');) **138(2):**870-881.

131.[**Takahashi S**](http://www.ncbi.nlm.nih.gov/sites/entrez?Db=pubmed&Cmd=Search&Term="Takahashi S"%5BAuthor%5D&itool=EntrezSystem2.PEntrez.Pubmed.Pubmed_ResultsPanel.Pubmed_RVAbstract)**,** [**Sakamoto AN**](http://www.ncbi.nlm.nih.gov/sites/entrez?Db=pubmed&Cmd=Search&Term="Sakamoto AN"%5BAuthor%5D&itool=EntrezSystem2.PEntrez.Pubmed.Pubmed_ResultsPanel.Pubmed_RVAbstract)**,** [**Tanaka A**](http://www.ncbi.nlm.nih.gov/sites/entrez?Db=pubmed&Cmd=Search&Term="Tanaka A"%5BAuthor%5D&itool=EntrezSystem2.PEntrez.Pubmed.Pubmed_ResultsPanel.Pubmed_RVAbstract)**,** [**Shimizu K**](http://www.ncbi.nlm.nih.gov/sites/entrez?Db=pubmed&Cmd=Search&Term="Shimizu K"%5BAuthor%5D&itool=EntrezSystem2.PEntrez.Pubmed.Pubmed_ResultsPanel.Pubmed_RVAbstract) (2007) AtREV1, a Y-family DNA polymerase in Arabidopsis, has deoxynucleotidyl transferase activity in vitro. [Plant Physiol.](javascript:AL_get(this, 'jour', 'Plant Physiol.');) **145(3):**1052-1060.

132.[**Sakamoto A**](http://www.ncbi.nlm.nih.gov/sites/entrez?Db=pubmed&Cmd=Search&Term="Sakamoto A"%5BAuthor%5D&itool=EntrezSystem2.PEntrez.Pubmed.Pubmed_ResultsPanel.Pubmed_RVAbstract)**,** [**Lan VT**](http://www.ncbi.nlm.nih.gov/sites/entrez?Db=pubmed&Cmd=Search&Term="Lan VT"%5BAuthor%5D&itool=EntrezSystem2.PEntrez.Pubmed.Pubmed_ResultsPanel.Pubmed_RVAbstract)**,** [**Hase Y**](http://www.ncbi.nlm.nih.gov/sites/entrez?Db=pubmed&Cmd=Search&Term="Hase Y"%5BAuthor%5D&itool=EntrezSystem2.PEntrez.Pubmed.Pubmed_ResultsPanel.Pubmed_RVAbstract)**,** [**Shikazono N**](http://www.ncbi.nlm.nih.gov/sites/entrez?Db=pubmed&Cmd=Search&Term="Shikazono N"%5BAuthor%5D&itool=EntrezSystem2.PEntrez.Pubmed.Pubmed_ResultsPanel.Pubmed_RVAbstract)**,** [**Matsunaga T**](http://www.ncbi.nlm.nih.gov/sites/entrez?Db=pubmed&Cmd=Search&Term="Matsunaga T"%5BAuthor%5D&itool=EntrezSystem2.PEntrez.Pubmed.Pubmed_ResultsPanel.Pubmed_RVAbstract)**,** [**Tanaka A**](http://www.ncbi.nlm.nih.gov/sites/entrez?Db=pubmed&Cmd=Search&Term="Tanaka A"%5BAuthor%5D&itool=EntrezSystem2.PEntrez.Pubmed.Pubmed_ResultsPanel.Pubmed_RVAbstract) ( 2003 ) Disruption of the AtREV3 gene causes hypersensitivity to ultraviolet B light and gamma-rays in Arabidopsis: implication of the presence of a translesion synthesis mechanism in plants. [Plant Cell](javascript:AL_get(this, 'jour', 'Plant Cell.');) **15(9):**2042-2057.

133.[**Yin H**](http://www.ncbi.nlm.nih.gov/sites/entrez?Db=pubmed&Cmd=Search&Term="Yin H"%5BAuthor%5D&itool=EntrezSystem2.PEntrez.Pubmed.Pubmed_ResultsPanel.Pubmed_RVAbstract)**,** [**Zhang X**](http://www.ncbi.nlm.nih.gov/sites/entrez?Db=pubmed&Cmd=Search&Term="Zhang X"%5BAuthor%5D&itool=EntrezSystem2.PEntrez.Pubmed.Pubmed_ResultsPanel.Pubmed_RVAbstract)**,** [**Liu J**](http://www.ncbi.nlm.nih.gov/sites/entrez?Db=pubmed&Cmd=Search&Term="Liu J"%5BAuthor%5D&itool=EntrezSystem2.PEntrez.Pubmed.Pubmed_ResultsPanel.Pubmed_RVAbstract)**,** [**Wang Y**](http://www.ncbi.nlm.nih.gov/sites/entrez?Db=pubmed&Cmd=Search&Term="Wang Y"%5BAuthor%5D&itool=EntrezSystem2.PEntrez.Pubmed.Pubmed_ResultsPanel.Pubmed_RVAbstract)**,** [**He J**](http://www.ncbi.nlm.nih.gov/sites/entrez?Db=pubmed&Cmd=Search&Term="He J"%5BAuthor%5D&itool=EntrezSystem2.PEntrez.Pubmed.Pubmed_ResultsPanel.Pubmed_RVAbstract)**,** [**Yang T**](http://www.ncbi.nlm.nih.gov/sites/entrez?Db=pubmed&Cmd=Search&Term="Yang T"%5BAuthor%5D&itool=EntrezSystem2.PEntrez.Pubmed.Pubmed_ResultsPanel.Pubmed_RVAbstract)**,** [**Hong X**](http://www.ncbi.nlm.nih.gov/sites/entrez?Db=pubmed&Cmd=Search&Term="Hong X"%5BAuthor%5D&itool=EntrezSystem2.PEntrez.Pubmed.Pubmed_ResultsPanel.Pubmed_RVAbstract)**,** [**Yang Q**](http://www.ncbi.nlm.nih.gov/sites/entrez?Db=pubmed&Cmd=Search&Term="Yang Q"%5BAuthor%5D&itool=EntrezSystem2.PEntrez.Pubmed.Pubmed_ResultsPanel.Pubmed_RVAbstract)**,** [**Gong Z**](http://www.ncbi.nlm.nih.gov/sites/entrez?Db=pubmed&Cmd=Search&Term="Gong Z"%5BAuthor%5D&itool=EntrezSystem2.PEntrez.Pubmed.Pubmed_ResultsPanel.Pubmed_RVAbstract) (2009) Epigenetic Regulation, Somatic Homologous Recombination, and Abscisic Acid Signaling Are Influenced by DNA Polymerase {epsilon} Mutation in Arabidopsis. [Plant Cell](javascript:AL_get(this, 'jour', 'Plant Cell.');) **21(2):**386-402.

134.[**Kimura S**](http://www.ncbi.nlm.nih.gov/sites/entrez?Db=pubmed&Cmd=Search&Term="Kimura S"%5BAuthor%5D&itool=EntrezSystem2.PEntrez.Pubmed.Pubmed_ResultsPanel.Pubmed_DiscoveryPanel.Pubmed_RVAbstractPlus)**,** [**Suzuki T**](http://www.ncbi.nlm.nih.gov/sites/entrez?Db=pubmed&Cmd=Search&Term="Suzuki T"%5BAuthor%5D&itool=EntrezSystem2.PEntrez.Pubmed.Pubmed_ResultsPanel.Pubmed_DiscoveryPanel.Pubmed_RVAbstractPlus)**,** [**Yanagawa Y**](http://www.ncbi.nlm.nih.gov/sites/entrez?Db=pubmed&Cmd=Search&Term="Yanagawa Y"%5BAuthor%5D&itool=EntrezSystem2.PEntrez.Pubmed.Pubmed_ResultsPanel.Pubmed_DiscoveryPanel.Pubmed_RVAbstractPlus)**,** [**Yamamoto T**](http://www.ncbi.nlm.nih.gov/sites/entrez?Db=pubmed&Cmd=Search&Term="Yamamoto T"%5BAuthor%5D&itool=EntrezSystem2.PEntrez.Pubmed.Pubmed_ResultsPanel.Pubmed_DiscoveryPanel.Pubmed_RVAbstractPlus)**,** [**Nakagawa H**](http://www.ncbi.nlm.nih.gov/sites/entrez?Db=pubmed&Cmd=Search&Term="Nakagawa H"%5BAuthor%5D&itool=EntrezSystem2.PEntrez.Pubmed.Pubmed_ResultsPanel.Pubmed_DiscoveryPanel.Pubmed_RVAbstractPlus)**,** [**Tanaka I**](http://www.ncbi.nlm.nih.gov/sites/entrez?Db=pubmed&Cmd=Search&Term="Tanaka I"%5BAuthor%5D&itool=EntrezSystem2.PEntrez.Pubmed.Pubmed_ResultsPanel.Pubmed_DiscoveryPanel.Pubmed_RVAbstractPlus)**,** [**Hashimoto J**](http://www.ncbi.nlm.nih.gov/sites/entrez?Db=pubmed&Cmd=Search&Term="Hashimoto J"%5BAuthor%5D&itool=EntrezSystem2.PEntrez.Pubmed.Pubmed_ResultsPanel.Pubmed_DiscoveryPanel.Pubmed_RVAbstractPlus)**,** [**Sakaguchi K**](http://www.ncbi.nlm.nih.gov/sites/entrez?Db=pubmed&Cmd=Search&Term="Sakaguchi K"%5BAuthor%5D&itool=EntrezSystem2.PEntrez.Pubmed.Pubmed_ResultsPanel.Pubmed_DiscoveryPanel.Pubmed_RVAbstractPlus)(2001) Characterization of plant proliferating cell nuclear antigen (PCNA) and flap endonuclease-1 (FEN-1), and their distribution in mitotic and meiotic cell cycles. [Plant J.](javascript:AL_get(this, 'jour', 'Plant J.');) **28(6):**643-53.

135.[**Uchiyama Y**](http://www.ncbi.nlm.nih.gov/sites/entrez?Db=pubmed&Cmd=Search&Term="Uchiyama Y"%5BAuthor%5D&itool=EntrezSystem2.PEntrez.Pubmed.Pubmed_ResultsPanel.Pubmed_DiscoveryPanel.Pubmed_RVAbstractPlus)**,** [**Kimura S**](http://www.ncbi.nlm.nih.gov/sites/entrez?Db=pubmed&Cmd=Search&Term="Kimura S"%5BAuthor%5D&itool=EntrezSystem2.PEntrez.Pubmed.Pubmed_ResultsPanel.Pubmed_DiscoveryPanel.Pubmed_RVAbstractPlus)**,** [**Yamamoto T**](http://www.ncbi.nlm.nih.gov/sites/entrez?Db=pubmed&Cmd=Search&Term="Yamamoto T"%5BAuthor%5D&itool=EntrezSystem2.PEntrez.Pubmed.Pubmed_ResultsPanel.Pubmed_DiscoveryPanel.Pubmed_RVAbstractPlus)**,** [**Ishibashi T**](http://www.ncbi.nlm.nih.gov/sites/entrez?Db=pubmed&Cmd=Search&Term="Ishibashi T"%5BAuthor%5D&itool=EntrezSystem2.PEntrez.Pubmed.Pubmed_ResultsPanel.Pubmed_DiscoveryPanel.Pubmed_RVAbstractPlus)**,** [**Sakaguchi K**](http://www.ncbi.nlm.nih.gov/sites/entrez?Db=pubmed&Cmd=Search&Term="Sakaguchi K"%5BAuthor%5D&itool=EntrezSystem2.PEntrez.Pubmed.Pubmed_ResultsPanel.Pubmed_DiscoveryPanel.Pubmed_RVAbstractPlus) (2004 )Plant DNA polymerase lambda, a DNA repair enzyme that functions in plant meristematic and meiotic tissues. [Eur J Biochem.](javascript:AL_get(this, 'jour', 'Eur J Biochem.');) **271(13):**2799-2807.

136.[**Uchiyama Y**](http://www.ncbi.nlm.nih.gov/sites/entrez?Db=pubmed&Cmd=Search&Term="Uchiyama Y"%5BAuthor%5D&itool=EntrezSystem2.PEntrez.Pubmed.Pubmed_ResultsPanel.Pubmed_DiscoveryPanel.Pubmed_RVAbstractPlus)**,** [**Hatanaka M**](http://www.ncbi.nlm.nih.gov/sites/entrez?Db=pubmed&Cmd=Search&Term="Hatanaka M"%5BAuthor%5D&itool=EntrezSystem2.PEntrez.Pubmed.Pubmed_ResultsPanel.Pubmed_DiscoveryPanel.Pubmed_RVAbstractPlus)**,** [**Kimura S**](http://www.ncbi.nlm.nih.gov/sites/entrez?Db=pubmed&Cmd=Search&Term="Kimura S"%5BAuthor%5D&itool=EntrezSystem2.PEntrez.Pubmed.Pubmed_ResultsPanel.Pubmed_DiscoveryPanel.Pubmed_RVAbstractPlus)**,** [**Ishibashi T**](http://www.ncbi.nlm.nih.gov/sites/entrez?Db=pubmed&Cmd=Search&Term="Ishibashi T"%5BAuthor%5D&itool=EntrezSystem2.PEntrez.Pubmed.Pubmed_ResultsPanel.Pubmed_DiscoveryPanel.Pubmed_RVAbstractPlus)**,** [**Ueda T**](http://www.ncbi.nlm.nih.gov/sites/entrez?Db=pubmed&Cmd=Search&Term="Ueda T"%5BAuthor%5D&itool=EntrezSystem2.PEntrez.Pubmed.Pubmed_ResultsPanel.Pubmed_DiscoveryPanel.Pubmed_RVAbstractPlus)**,** [**Sakakibara Y**](http://www.ncbi.nlm.nih.gov/sites/entrez?Db=pubmed&Cmd=Search&Term="Sakakibara Y"%5BAuthor%5D&itool=EntrezSystem2.PEntrez.Pubmed.Pubmed_ResultsPanel.Pubmed_DiscoveryPanel.Pubmed_RVAbstractPlus)**,** [**Matsumoto T**](http://www.ncbi.nlm.nih.gov/sites/entrez?Db=pubmed&Cmd=Search&Term="Matsumoto T"%5BAuthor%5D&itool=EntrezSystem2.PEntrez.Pubmed.Pubmed_ResultsPanel.Pubmed_DiscoveryPanel.Pubmed_RVAbstractPlus)**,** [**Furukawa T**](http://www.ncbi.nlm.nih.gov/sites/entrez?Db=pubmed&Cmd=Search&Term="Furukawa T"%5BAuthor%5D&itool=EntrezSystem2.PEntrez.Pubmed.Pubmed_ResultsPanel.Pubmed_DiscoveryPanel.Pubmed_RVAbstractPlus)**,** [**Hashimoto J**](http://www.ncbi.nlm.nih.gov/sites/entrez?Db=pubmed&Cmd=Search&Term="Hashimoto J"%5BAuthor%5D&itool=EntrezSystem2.PEntrez.Pubmed.Pubmed_ResultsPanel.Pubmed_DiscoveryPanel.Pubmed_RVAbstractPlus)**,** [**Sakaguchi K**](http://www.ncbi.nlm.nih.gov/sites/entrez?Db=pubmed&Cmd=Search&Term="Sakaguchi K"%5BAuthor%5D&itool=EntrezSystem2.PEntrez.Pubmed.Pubmed_ResultsPanel.Pubmed_DiscoveryPanel.Pubmed_RVAbstractPlus)(2002) Characterization of DNA polymerase delta from a higher plant, rice (Oryza sativa L.). [Gene.](javascript:AL_get(this, 'jour', 'Gene.');) **295(1):**19-26.

137. **Sullivan ML and Vierstra RD** (1991). Cloning of a 16-kda ubiquitin carrier protein from wheat and Arabidopsis thaliana - identification of functional domains by in vitro mutagenesis. J Biol Chem. **266**: 23878-23885.

138. **Sullivan ML, Carpenter TB, Vierstra RD** (1994) Homologs of wheat ubiquitin-conjugating enzymes - TAUBC1 and TAUBC4 are encoded by small multigene families in Arabidopsis thaliana. Plant Mol. Biol. **24**: 651-661.

139.[**Wen R**](http://www.ncbi.nlm.nih.gov/sites/entrez?Db=pubmed&Cmd=Search&Term="Wen R"%5BAuthor%5D&itool=EntrezSystem2.PEntrez.Pubmed.Pubmed_ResultsPanel.Pubmed_RVAbstract)**,** [**Torres-Acosta JA**](http://www.ncbi.nlm.nih.gov/sites/entrez?Db=pubmed&Cmd=Search&Term="Torres-Acosta JA"%5BAuthor%5D&itool=EntrezSystem2.PEntrez.Pubmed.Pubmed_ResultsPanel.Pubmed_RVAbstract)**,** [**Pastushok L**](http://www.ncbi.nlm.nih.gov/sites/entrez?Db=pubmed&Cmd=Search&Term="Pastushok L"%5BAuthor%5D&itool=EntrezSystem2.PEntrez.Pubmed.Pubmed_ResultsPanel.Pubmed_RVAbstract)**,** [**Lai X**](http://www.ncbi.nlm.nih.gov/sites/entrez?Db=pubmed&Cmd=Search&Term="Lai X"%5BAuthor%5D&itool=EntrezSystem2.PEntrez.Pubmed.Pubmed_ResultsPanel.Pubmed_RVAbstract)**,** [**Pelzer L**](http://www.ncbi.nlm.nih.gov/sites/entrez?Db=pubmed&Cmd=Search&Term="Pelzer L"%5BAuthor%5D&itool=EntrezSystem2.PEntrez.Pubmed.Pubmed_ResultsPanel.Pubmed_RVAbstract)**,** [**Wang H**](http://www.ncbi.nlm.nih.gov/sites/entrez?Db=pubmed&Cmd=Search&Term="Wang H"%5BAuthor%5D&itool=EntrezSystem2.PEntrez.Pubmed.Pubmed_ResultsPanel.Pubmed_RVAbstract)**,** [**Xiao W**](http://www.ncbi.nlm.nih.gov/sites/entrez?Db=pubmed&Cmd=Search&Term="Xiao W"%5BAuthor%5D&itool=EntrezSystem2.PEntrez.Pubmed.Pubmed_ResultsPanel.Pubmed_RVAbstract) (2008) Arabidopsis UEV1D promotes Lysine-63-linked polyubiquitination and is involved in DNA damage response. Plant J **20(1):**213-227.

140.[**Wen R**](http://www.ncbi.nlm.nih.gov/sites/entrez?Db=pubmed&Cmd=Search&Term="Wen R"%5BAuthor%5D&itool=EntrezSystem2.PEntrez.Pubmed.Pubmed_ResultsPanel.Pubmed_RVAbstract)**,** [**Newton L**](http://www.ncbi.nlm.nih.gov/sites/entrez?Db=pubmed&Cmd=Search&Term="Newton L"%5BAuthor%5D&itool=EntrezSystem2.PEntrez.Pubmed.Pubmed_ResultsPanel.Pubmed_RVAbstract)**,** [**Li G**](http://www.ncbi.nlm.nih.gov/sites/entrez?Db=pubmed&Cmd=Search&Term="Li G"%5BAuthor%5D&itool=EntrezSystem2.PEntrez.Pubmed.Pubmed_ResultsPanel.Pubmed_RVAbstract)**,** [**Wang H**](http://www.ncbi.nlm.nih.gov/sites/entrez?Db=pubmed&Cmd=Search&Term="Wang H"%5BAuthor%5D&itool=EntrezSystem2.PEntrez.Pubmed.Pubmed_ResultsPanel.Pubmed_RVAbstract)**,** [**Xiao W**](http://www.ncbi.nlm.nih.gov/sites/entrez?Db=pubmed&Cmd=Search&Term="Xiao W"%5BAuthor%5D&itool=EntrezSystem2.PEntrez.Pubmed.Pubmed_ResultsPanel.Pubmed_RVAbstract) (2006) Arabidopsis thaliana UBC13: implication of error-free DNA damage tolerance and Lys63-linked polyubiquitylation in plants.[Plant Mol Biol.](javascript:AL_get(this, 'jour', 'Plant Mol Biol.');) **61(1-2)**:241-253.

141.[**Bagherieh-Najjar MB**](http://www.ncbi.nlm.nih.gov/sites/entrez?Db=pubmed&Cmd=Search&Term="Bagherieh-Najjar MB"%5BAuthor%5D&itool=EntrezSystem2.PEntrez.Pubmed.Pubmed_ResultsPanel.Pubmed_RVAbstract)**,** [**de Vries OM**](http://www.ncbi.nlm.nih.gov/sites/entrez?Db=pubmed&Cmd=Search&Term="de Vries OM"%5BAuthor%5D&itool=EntrezSystem2.PEntrez.Pubmed.Pubmed_ResultsPanel.Pubmed_RVAbstract)**,** [**Kroon JT**](http://www.ncbi.nlm.nih.gov/sites/entrez?Db=pubmed&Cmd=Search&Term="Kroon JT"%5BAuthor%5D&itool=EntrezSystem2.PEntrez.Pubmed.Pubmed_ResultsPanel.Pubmed_RVAbstract)**,** [**Wright EL**](http://www.ncbi.nlm.nih.gov/sites/entrez?Db=pubmed&Cmd=Search&Term="Wright EL"%5BAuthor%5D&itool=EntrezSystem2.PEntrez.Pubmed.Pubmed_ResultsPanel.Pubmed_RVAbstract)**,** [**Elborough KM**](http://www.ncbi.nlm.nih.gov/sites/entrez?Db=pubmed&Cmd=Search&Term="Elborough KM"%5BAuthor%5D&itool=EntrezSystem2.PEntrez.Pubmed.Pubmed_ResultsPanel.Pubmed_RVAbstract)**,** [**Hille J**](http://www.ncbi.nlm.nih.gov/sites/entrez?Db=pubmed&Cmd=Search&Term="Hille J"%5BAuthor%5D&itool=EntrezSystem2.PEntrez.Pubmed.Pubmed_ResultsPanel.Pubmed_RVAbstract)**,** [**Dijkwel PP**](http://www.ncbi.nlm.nih.gov/sites/entrez?Db=pubmed&Cmd=Search&Term="Dijkwel PP"%5BAuthor%5D&itool=EntrezSystem2.PEntrez.Pubmed.Pubmed_ResultsPanel.Pubmed_RVAbstract) (2003) Arabidopsis RecQsim, a plant-specific member of the RecQ helicase family, can suppress the MMS hypersensitivity of the yeast sgs1 mutant.[Plant Mol Biol.](javascript:AL_get(this, 'jour', 'Plant Mol Biol.');) **52(2):**273-284.

142.[**Hartung F**](http://www.ncbi.nlm.nih.gov/sites/entrez?Db=pubmed&Cmd=Search&Term="Hartung F"%5BAuthor%5D&itool=EntrezSystem2.PEntrez.Pubmed.Pubmed_ResultsPanel.Pubmed_RVAbstract)**,** [**Plchová H**](http://www.ncbi.nlm.nih.gov/sites/entrez?Db=pubmed&Cmd=Search&Term="Plchová H"%5BAuthor%5D&itool=EntrezSystem2.PEntrez.Pubmed.Pubmed_ResultsPanel.Pubmed_RVAbstract)**,** [**Puchta H**](http://www.ncbi.nlm.nih.gov/sites/entrez?Db=pubmed&Cmd=Search&Term="Puchta H"%5BAuthor%5D&itool=EntrezSystem2.PEntrez.Pubmed.Pubmed_ResultsPanel.Pubmed_RVAbstract) (2000) Molecular characterization of RecQ homologues in Arabidopsis thaliana. [Nucleic Acids Res.](javascript:AL_get(this, 'jour', 'Nucleic Acids Res.');) **28(21):**4275-4282.

143.[**Kobbe D**](http://www.ncbi.nlm.nih.gov/sites/entrez?Db=pubmed&Cmd=Search&Term="Kobbe D"%5BAuthor%5D&itool=EntrezSystem2.PEntrez.Pubmed.Pubmed_ResultsPanel.Pubmed_DiscoveryPanel.Pubmed_RVAbstractPlus)**,** [**Blanck S**](http://www.ncbi.nlm.nih.gov/sites/entrez?Db=pubmed&Cmd=Search&Term="Blanck S"%5BAuthor%5D&itool=EntrezSystem2.PEntrez.Pubmed.Pubmed_ResultsPanel.Pubmed_DiscoveryPanel.Pubmed_RVAbstractPlus)**,** [**Demand K**](http://www.ncbi.nlm.nih.gov/sites/entrez?Db=pubmed&Cmd=Search&Term="Demand K"%5BAuthor%5D&itool=EntrezSystem2.PEntrez.Pubmed.Pubmed_ResultsPanel.Pubmed_DiscoveryPanel.Pubmed_RVAbstractPlus)**,** [**Focke M**](http://www.ncbi.nlm.nih.gov/sites/entrez?Db=pubmed&Cmd=Search&Term="Focke M"%5BAuthor%5D&itool=EntrezSystem2.PEntrez.Pubmed.Pubmed_ResultsPanel.Pubmed_DiscoveryPanel.Pubmed_RVAbstractPlus)**,** [**Puchta H**](http://www.ncbi.nlm.nih.gov/sites/entrez?Db=pubmed&Cmd=Search&Term="Puchta H"%5BAuthor%5D&itool=EntrezSystem2.PEntrez.Pubmed.Pubmed_ResultsPanel.Pubmed_DiscoveryPanel.Pubmed_RVAbstractPlus) (2008)AtRECQ2, a RecQ helicase homologue from Arabidopsis thaliana, is able to disrupt various recombinogenic DNA structures in vitro. [Plant J.](javascript:AL_get(this, 'jour', 'Plant J.');) **55(3):**397-405.

144.[**Kimura S**](http://www.ncbi.nlm.nih.gov/sites/entrez?Db=pubmed&Cmd=Search&Term="Kimura S"%5BAuthor%5D&itool=EntrezSystem2.PEntrez.Pubmed.Pubmed_ResultsPanel.Pubmed_DiscoveryPanel.Pubmed_RVAbstractPlus)**,** [**Saotome A**](http://www.ncbi.nlm.nih.gov/sites/entrez?Db=pubmed&Cmd=Search&Term="Saotome A"%5BAuthor%5D&itool=EntrezSystem2.PEntrez.Pubmed.Pubmed_ResultsPanel.Pubmed_DiscoveryPanel.Pubmed_RVAbstractPlus)**,** [**Uchiyama Y**](http://www.ncbi.nlm.nih.gov/sites/entrez?Db=pubmed&Cmd=Search&Term="Uchiyama Y"%5BAuthor%5D&itool=EntrezSystem2.PEntrez.Pubmed.Pubmed_ResultsPanel.Pubmed_DiscoveryPanel.Pubmed_RVAbstractPlus)**,** [**Mori Y**](http://www.ncbi.nlm.nih.gov/sites/entrez?Db=pubmed&Cmd=Search&Term="Mori Y"%5BAuthor%5D&itool=EntrezSystem2.PEntrez.Pubmed.Pubmed_ResultsPanel.Pubmed_DiscoveryPanel.Pubmed_RVAbstractPlus)**,** [**Tahira Y**](http://www.ncbi.nlm.nih.gov/sites/entrez?Db=pubmed&Cmd=Search&Term="Tahira Y"%5BAuthor%5D&itool=EntrezSystem2.PEntrez.Pubmed.Pubmed_ResultsPanel.Pubmed_DiscoveryPanel.Pubmed_RVAbstractPlus)**,** [**Sakaguchi K**](http://www.ncbi.nlm.nih.gov/sites/entrez?Db=pubmed&Cmd=Search&Term="Sakaguchi K"%5BAuthor%5D&itool=EntrezSystem2.PEntrez.Pubmed.Pubmed_ResultsPanel.Pubmed_DiscoveryPanel.Pubmed_RVAbstractPlus) (2005) The expression of the rice (Oryza sativa L.) homologue of Snm1 is induced by DNA damages. Biochem Biophys Res Commun. **329(2):**668-672

145.[**Molinier J**](http://www.ncbi.nlm.nih.gov/sites/entrez?Db=pubmed&Cmd=Search&Term="Molinier J"%5BAuthor%5D&itool=EntrezSystem2.PEntrez.Pubmed.Pubmed_ResultsPanel.Pubmed_DiscoveryPanel.Pubmed_RVAbstractPlus)**,** [**Stamm ME**](http://www.ncbi.nlm.nih.gov/sites/entrez?Db=pubmed&Cmd=Search&Term="Stamm ME"%5BAuthor%5D&itool=EntrezSystem2.PEntrez.Pubmed.Pubmed_ResultsPanel.Pubmed_DiscoveryPanel.Pubmed_RVAbstractPlus)**,** [**Hohn B**](http://www.ncbi.nlm.nih.gov/sites/entrez?Db=pubmed&Cmd=Search&Term="Hohn B"%5BAuthor%5D&itool=EntrezSystem2.PEntrez.Pubmed.Pubmed_ResultsPanel.Pubmed_DiscoveryPanel.Pubmed_RVAbstractPlus) (2004) SNM-dependent recombinational repair of oxidatively induced DNA damage in Arabidopsis thaliana. EMBO Rep. **5(10)**:994-999.

146.[**Saotome A**](http://www.ncbi.nlm.nih.gov/sites/entrez?Db=pubmed&Cmd=Search&Term="Saotome A"%5BAuthor%5D&itool=EntrezSystem2.PEntrez.Pubmed.Pubmed_ResultsPanel.Pubmed_DiscoveryPanel.Pubmed_RVAbstractPlus)**,** [**Kimura S**](http://www.ncbi.nlm.nih.gov/sites/entrez?Db=pubmed&Cmd=Search&Term="Kimura S"%5BAuthor%5D&itool=EntrezSystem2.PEntrez.Pubmed.Pubmed_ResultsPanel.Pubmed_DiscoveryPanel.Pubmed_RVAbstractPlus)**,** [**Mori Y**](http://www.ncbi.nlm.nih.gov/sites/entrez?Db=pubmed&Cmd=Search&Term="Mori Y"%5BAuthor%5D&itool=EntrezSystem2.PEntrez.Pubmed.Pubmed_ResultsPanel.Pubmed_DiscoveryPanel.Pubmed_RVAbstractPlus)**,** [**Uchiyama Y**](http://www.ncbi.nlm.nih.gov/sites/entrez?Db=pubmed&Cmd=Search&Term="Uchiyama Y"%5BAuthor%5D&itool=EntrezSystem2.PEntrez.Pubmed.Pubmed_ResultsPanel.Pubmed_DiscoveryPanel.Pubmed_RVAbstractPlus)**,** [**Morohashi K**](http://www.ncbi.nlm.nih.gov/sites/entrez?Db=pubmed&Cmd=Search&Term="Morohashi K"%5BAuthor%5D&itool=EntrezSystem2.PEntrez.Pubmed.Pubmed_ResultsPanel.Pubmed_DiscoveryPanel.Pubmed_RVAbstractPlus)**,** [**Sakaguchi K**](http://www.ncbi.nlm.nih.gov/sites/entrez?Db=pubmed&Cmd=Search&Term="Sakaguchi K"%5BAuthor%5D&itool=EntrezSystem2.PEntrez.Pubmed.Pubmed_ResultsPanel.Pubmed_DiscoveryPanel.Pubmed_RVAbstractPlus) (2006) Characterization of four RecQ homologues from rice (Oryza sativa L. cv. Nipponbare). [Biochem Biophys Res Commun.](javascript:AL_get(this, 'jour', 'Biochem Biophys Res Commun.');) **345(4):**1283-1291.

147.[**Plchova H**](http://www.ncbi.nlm.nih.gov/sites/entrez?Db=pubmed&Cmd=Search&Term="Plchova H"%5BAuthor%5D&itool=EntrezSystem2.PEntrez.Pubmed.Pubmed_ResultsPanel.Pubmed_RVAbstract)**,** [**Hartung F**](http://www.ncbi.nlm.nih.gov/sites/entrez?Db=pubmed&Cmd=Search&Term="Hartung F"%5BAuthor%5D&itool=EntrezSystem2.PEntrez.Pubmed.Pubmed_ResultsPanel.Pubmed_RVAbstract)**,** [**Puchta H**](http://www.ncbi.nlm.nih.gov/sites/entrez?Db=pubmed&Cmd=Search&Term="Puchta H"%5BAuthor%5D&itool=EntrezSystem2.PEntrez.Pubmed.Pubmed_ResultsPanel.Pubmed_RVAbstract) (2003) Biochemical characterization of an exonuclease from Arabidopsis thaliana reveals similarities to the DNA exonuclease of the human Werner syndrome protein. [J Biol Chem.](javascript:AL_get(this, 'jour', 'J Biol Chem.');) **278(45):**44128-44138.

148.[**Ricaud L**](http://www.ncbi.nlm.nih.gov/sites/entrez?Db=pubmed&Cmd=Search&Term="Ricaud L"%5BAuthor%5D&itool=EntrezSystem2.PEntrez.Pubmed.Pubmed_ResultsPanel.Pubmed_RVAbstract)**,** [**Proux C**](http://www.ncbi.nlm.nih.gov/sites/entrez?Db=pubmed&Cmd=Search&Term="Proux C"%5BAuthor%5D&itool=EntrezSystem2.PEntrez.Pubmed.Pubmed_ResultsPanel.Pubmed_RVAbstract)**,** [**Renou JP**](http://www.ncbi.nlm.nih.gov/sites/entrez?Db=pubmed&Cmd=Search&Term="Renou JP"%5BAuthor%5D&itool=EntrezSystem2.PEntrez.Pubmed.Pubmed_ResultsPanel.Pubmed_RVAbstract)**,** [**Pichon O**](http://www.ncbi.nlm.nih.gov/sites/entrez?Db=pubmed&Cmd=Search&Term="Pichon O"%5BAuthor%5D&itool=EntrezSystem2.PEntrez.Pubmed.Pubmed_ResultsPanel.Pubmed_RVAbstract)**,** [**Fochesato S**](http://www.ncbi.nlm.nih.gov/sites/entrez?Db=pubmed&Cmd=Search&Term="Fochesato S"%5BAuthor%5D&itool=EntrezSystem2.PEntrez.Pubmed.Pubmed_ResultsPanel.Pubmed_RVAbstract)**,** [**Ortet P**](http://www.ncbi.nlm.nih.gov/sites/entrez?Db=pubmed&Cmd=Search&Term="Ortet P"%5BAuthor%5D&itool=EntrezSystem2.PEntrez.Pubmed.Pubmed_ResultsPanel.Pubmed_RVAbstract)**,** [**Montané MH**](http://www.ncbi.nlm.nih.gov/sites/entrez?Db=pubmed&Cmd=Search&Term="Montané MH"%5BAuthor%5D&itool=EntrezSystem2.PEntrez.Pubmed.Pubmed_ResultsPanel.Pubmed_RVAbstract) (2007) ATM-mediated transcriptional and developmental responses to gamma-rays in Arabidopsis.[PLoS ONE](javascript:AL_get(this, 'jour', 'PLoS ONE.');) 2(5):e430.

149.[**Culligan KM**](http://www.ncbi.nlm.nih.gov/sites/entrez?Db=pubmed&Cmd=Search&Term="Culligan KM"%5BAuthor%5D&itool=EntrezSystem2.PEntrez.Pubmed.Pubmed_ResultsPanel.Pubmed_RVAbstract)**,** [**Britt AB**](http://www.ncbi.nlm.nih.gov/sites/entrez?Db=pubmed&Cmd=Search&Term="Britt AB"%5BAuthor%5D&itool=EntrezSystem2.PEntrez.Pubmed.Pubmed_ResultsPanel.Pubmed_RVAbstract) (2008) Both ATM and ATR promote the efficient and accurate processing of programmed meiotic double-strand breaks.[Plant J.](javascript:AL_get(this, 'jour', 'Plant J.');) **55(4):**629-638.

150.[**Vespa L**](http://www.ncbi.nlm.nih.gov/sites/entrez?Db=pubmed&Cmd=Search&Term="Vespa L"%5BAuthor%5D&itool=EntrezSystem2.PEntrez.Pubmed.Pubmed_ResultsPanel.Pubmed_RVAbstract)**,** [**Warrington RT**](http://www.ncbi.nlm.nih.gov/sites/entrez?Db=pubmed&Cmd=Search&Term="Warrington RT"%5BAuthor%5D&itool=EntrezSystem2.PEntrez.Pubmed.Pubmed_ResultsPanel.Pubmed_RVAbstract)**,** [**Mokros P**](http://www.ncbi.nlm.nih.gov/sites/entrez?Db=pubmed&Cmd=Search&Term="Mokros P"%5BAuthor%5D&itool=EntrezSystem2.PEntrez.Pubmed.Pubmed_ResultsPanel.Pubmed_RVAbstract)**,** [**Siroky J**](http://www.ncbi.nlm.nih.gov/sites/entrez?Db=pubmed&Cmd=Search&Term="Siroky J"%5BAuthor%5D&itool=EntrezSystem2.PEntrez.Pubmed.Pubmed_ResultsPanel.Pubmed_RVAbstract)**,** [**Shippen DE**](http://www.ncbi.nlm.nih.gov/sites/entrez?Db=pubmed&Cmd=Search&Term="Shippen DE"%5BAuthor%5D&itool=EntrezSystem2.PEntrez.Pubmed.Pubmed_ResultsPanel.Pubmed_RVAbstract) (2007)ATM regulates the length of individual telomere tracts in Arabidopsis.[Proc Natl Acad Sci U S A.](javascript:AL_get(this, 'jour', 'Proc Natl Acad Sci U S A.');) **104(46):**18145-18150.

151.[**Garcia V**](http://www.ncbi.nlm.nih.gov/sites/entrez?Db=pubmed&Cmd=Search&Term="Garcia V"%5BAuthor%5D&itool=EntrezSystem2.PEntrez.Pubmed.Pubmed_ResultsPanel.Pubmed_DiscoveryPanel.Pubmed_RVAbstractPlus)**,** [**Bruchet H**](http://www.ncbi.nlm.nih.gov/sites/entrez?Db=pubmed&Cmd=Search&Term="Bruchet H"%5BAuthor%5D&itool=EntrezSystem2.PEntrez.Pubmed.Pubmed_ResultsPanel.Pubmed_DiscoveryPanel.Pubmed_RVAbstractPlus)**,** [**Camescasse D**](http://www.ncbi.nlm.nih.gov/sites/entrez?Db=pubmed&Cmd=Search&Term="Camescasse D"%5BAuthor%5D&itool=EntrezSystem2.PEntrez.Pubmed.Pubmed_ResultsPanel.Pubmed_DiscoveryPanel.Pubmed_RVAbstractPlus)**,** [**Granier F**](http://www.ncbi.nlm.nih.gov/sites/entrez?Db=pubmed&Cmd=Search&Term="Granier F"%5BAuthor%5D&itool=EntrezSystem2.PEntrez.Pubmed.Pubmed_ResultsPanel.Pubmed_DiscoveryPanel.Pubmed_RVAbstractPlus)**,** [**Bouchez D**](http://www.ncbi.nlm.nih.gov/sites/entrez?Db=pubmed&Cmd=Search&Term="Bouchez D"%5BAuthor%5D&itool=EntrezSystem2.PEntrez.Pubmed.Pubmed_ResultsPanel.Pubmed_DiscoveryPanel.Pubmed_RVAbstractPlus)**,** [**Tissier A**](http://www.ncbi.nlm.nih.gov/sites/entrez?Db=pubmed&Cmd=Search&Term="Tissier A"%5BAuthor%5D&itool=EntrezSystem2.PEntrez.Pubmed.Pubmed_ResultsPanel.Pubmed_DiscoveryPanel.Pubmed_RVAbstractPlus) (2003) AtATM is essential for meiosis and the somatic response to DNA damage in plants. [Plant Cell](javascript:AL_get(this, 'jour', 'Plant Cell.');) **15(1):**119-132

152. **Culligan K, Tissier A, Britt A** (2004). ATR Regulates a G2-Phase Cell-Cycle Checkpoint in Arabidopsis thaliana. Plant Cell **16**: 1091-1104

153.[**Heitzeberg F**](http://www.ncbi.nlm.nih.gov/sites/entrez?Db=pubmed&Cmd=Search&Term="Heitzeberg F"%5BAuthor%5D&itool=EntrezSystem2.PEntrez.Pubmed.Pubmed_ResultsPanel.Pubmed_RVAbstract)**,** [**Chen IP**](http://www.ncbi.nlm.nih.gov/sites/entrez?Db=pubmed&Cmd=Search&Term="Chen IP"%5BAuthor%5D&itool=EntrezSystem2.PEntrez.Pubmed.Pubmed_ResultsPanel.Pubmed_RVAbstract)**,** [**Hartung F**](http://www.ncbi.nlm.nih.gov/sites/entrez?Db=pubmed&Cmd=Search&Term="Hartung F"%5BAuthor%5D&itool=EntrezSystem2.PEntrez.Pubmed.Pubmed_ResultsPanel.Pubmed_RVAbstract)**,** [**Orel N**](http://www.ncbi.nlm.nih.gov/sites/entrez?Db=pubmed&Cmd=Search&Term="Orel N"%5BAuthor%5D&itool=EntrezSystem2.PEntrez.Pubmed.Pubmed_ResultsPanel.Pubmed_RVAbstract)**,** [**Angelis KJ**](http://www.ncbi.nlm.nih.gov/sites/entrez?Db=pubmed&Cmd=Search&Term="Angelis KJ"%5BAuthor%5D&itool=EntrezSystem2.PEntrez.Pubmed.Pubmed_ResultsPanel.Pubmed_RVAbstract)**,** [**Puchta H**](http://www.ncbi.nlm.nih.gov/sites/entrez?Db=pubmed&Cmd=Search&Term="Puchta H"%5BAuthor%5D&itool=EntrezSystem2.PEntrez.Pubmed.Pubmed_ResultsPanel.Pubmed_RVAbstract) (2004) The Rad17 homologue of Arabidopsis is involved in the regulation of DNA damage repair and homologous recombination. [Plant J.](javascript:AL_get(this, 'jour', 'Plant J.');) **38(6):**954-68.

154.[**Pang Q**](http://www.ncbi.nlm.nih.gov/sites/entrez?Db=pubmed&Cmd=Search&Term="Pang Q"%5BAuthor%5D&itool=EntrezSystem2.PEntrez.Pubmed.Pubmed_ResultsPanel.Pubmed_RVAbstract)**,** [**Hays JB**](http://www.ncbi.nlm.nih.gov/sites/entrez?Db=pubmed&Cmd=Search&Term="Hays JB"%5BAuthor%5D&itool=EntrezSystem2.PEntrez.Pubmed.Pubmed_ResultsPanel.Pubmed_RVAbstract)**,** [**Rajagopal I**](http://www.ncbi.nlm.nih.gov/sites/entrez?Db=pubmed&Cmd=Search&Term="Rajagopal I"%5BAuthor%5D&itool=EntrezSystem2.PEntrez.Pubmed.Pubmed_ResultsPanel.Pubmed_RVAbstract)**,** [**Schaefer TS**](http://www.ncbi.nlm.nih.gov/sites/entrez?Db=pubmed&Cmd=Search&Term="Schaefer TS"%5BAuthor%5D&itool=EntrezSystem2.PEntrez.Pubmed.Pubmed_ResultsPanel.Pubmed_RVAbstract) (1993) Selection of Arabidopsis cDNAs that partially correct phenotypes of Escherichia coli DNA-damage-sensitive mutants and analysis of two plant cDNAs that appear to express UV-specific dark repair activities. [Plant Mol Biol.](javascript:AL_get(this, 'jour', 'Plant Mol Biol.');) **22(3):**411-426.

155.[**Favory JJ**](http://www.ncbi.nlm.nih.gov/sites/entrez?Db=pubmed&Cmd=Search&Term="Favory JJ"%5BAuthor%5D&itool=EntrezSystem2.PEntrez.Pubmed.Pubmed_ResultsPanel.Pubmed_RVAbstract)**,** [**Stec A**](http://www.ncbi.nlm.nih.gov/sites/entrez?Db=pubmed&Cmd=Search&Term="Stec A"%5BAuthor%5D&itool=EntrezSystem2.PEntrez.Pubmed.Pubmed_ResultsPanel.Pubmed_RVAbstract)**,** [**Gruber H**](http://www.ncbi.nlm.nih.gov/sites/entrez?Db=pubmed&Cmd=Search&Term="Gruber H"%5BAuthor%5D&itool=EntrezSystem2.PEntrez.Pubmed.Pubmed_ResultsPanel.Pubmed_RVAbstract)**,** [**Rizzini L**](http://www.ncbi.nlm.nih.gov/sites/entrez?Db=pubmed&Cmd=Search&Term="Rizzini L"%5BAuthor%5D&itool=EntrezSystem2.PEntrez.Pubmed.Pubmed_ResultsPanel.Pubmed_RVAbstract)**,** [**Oravecz A**](http://www.ncbi.nlm.nih.gov/sites/entrez?Db=pubmed&Cmd=Search&Term="Oravecz A"%5BAuthor%5D&itool=EntrezSystem2.PEntrez.Pubmed.Pubmed_ResultsPanel.Pubmed_RVAbstract)**,** [**Funk M**](http://www.ncbi.nlm.nih.gov/sites/entrez?Db=pubmed&Cmd=Search&Term="Funk M"%5BAuthor%5D&itool=EntrezSystem2.PEntrez.Pubmed.Pubmed_ResultsPanel.Pubmed_RVAbstract)**,** [**Albert A**](http://www.ncbi.nlm.nih.gov/sites/entrez?Db=pubmed&Cmd=Search&Term="Albert A"%5BAuthor%5D&itool=EntrezSystem2.PEntrez.Pubmed.Pubmed_ResultsPanel.Pubmed_RVAbstract)**,** [**Cloix C**](http://www.ncbi.nlm.nih.gov/sites/entrez?Db=pubmed&Cmd=Search&Term="Cloix C"%5BAuthor%5D&itool=EntrezSystem2.PEntrez.Pubmed.Pubmed_ResultsPanel.Pubmed_RVAbstract)**,** [**Jenkins GI**](http://www.ncbi.nlm.nih.gov/sites/entrez?Db=pubmed&Cmd=Search&Term="Jenkins GI"%5BAuthor%5D&itool=EntrezSystem2.PEntrez.Pubmed.Pubmed_ResultsPanel.Pubmed_RVAbstract)**,** [**Oakeley EJ**](http://www.ncbi.nlm.nih.gov/sites/entrez?Db=pubmed&Cmd=Search&Term="Oakeley EJ"%5BAuthor%5D&itool=EntrezSystem2.PEntrez.Pubmed.Pubmed_ResultsPanel.Pubmed_RVAbstract)**,** [**Seidlitz HK**](http://www.ncbi.nlm.nih.gov/sites/entrez?Db=pubmed&Cmd=Search&Term="Seidlitz HK"%5BAuthor%5D&itool=EntrezSystem2.PEntrez.Pubmed.Pubmed_ResultsPanel.Pubmed_RVAbstract)**,** [**Nagy F**](http://www.ncbi.nlm.nih.gov/sites/entrez?Db=pubmed&Cmd=Search&Term="Nagy F"%5BAuthor%5D&itool=EntrezSystem2.PEntrez.Pubmed.Pubmed_ResultsPanel.Pubmed_RVAbstract)**,** [**Ulm R**](http://www.ncbi.nlm.nih.gov/sites/entrez?Db=pubmed&Cmd=Search&Term="Ulm R"%5BAuthor%5D&itool=EntrezSystem2.PEntrez.Pubmed.Pubmed_ResultsPanel.Pubmed_RVAbstract) (2009) Interaction of COP1 and UVR8 regulates UV-B-induced photomorphogenesis and stress acclimation in Arabidopsis. [EMBO J.](javascript:AL_get(this, 'jour', 'EMBO J.');) **28(5):**591-601

156.[**Nakajima S**](http://www.ncbi.nlm.nih.gov/sites/entrez?Db=pubmed&Cmd=Search&Term="Nakajima S"%5BAuthor%5D&itool=EntrezSystem2.PEntrez.Pubmed.Pubmed_ResultsPanel.Pubmed_RVAbstract)**,** [**Sugiyama M**](http://www.ncbi.nlm.nih.gov/sites/entrez?Db=pubmed&Cmd=Search&Term="Sugiyama M"%5BAuthor%5D&itool=EntrezSystem2.PEntrez.Pubmed.Pubmed_ResultsPanel.Pubmed_RVAbstract)**,** [**Iwai S**](http://www.ncbi.nlm.nih.gov/sites/entrez?Db=pubmed&Cmd=Search&Term="Iwai S"%5BAuthor%5D&itool=EntrezSystem2.PEntrez.Pubmed.Pubmed_ResultsPanel.Pubmed_RVAbstract)**,** [**Hitomi K**](http://www.ncbi.nlm.nih.gov/sites/entrez?Db=pubmed&Cmd=Search&Term="Hitomi K"%5BAuthor%5D&itool=EntrezSystem2.PEntrez.Pubmed.Pubmed_ResultsPanel.Pubmed_RVAbstract)**,** [**Otoshi E**](http://www.ncbi.nlm.nih.gov/sites/entrez?Db=pubmed&Cmd=Search&Term="Otoshi E"%5BAuthor%5D&itool=EntrezSystem2.PEntrez.Pubmed.Pubmed_ResultsPanel.Pubmed_RVAbstract)**,** [**Kim ST**](http://www.ncbi.nlm.nih.gov/sites/entrez?Db=pubmed&Cmd=Search&Term="Kim ST"%5BAuthor%5D&itool=EntrezSystem2.PEntrez.Pubmed.Pubmed_ResultsPanel.Pubmed_RVAbstract)**,** [**Jiang CZ**](http://www.ncbi.nlm.nih.gov/sites/entrez?Db=pubmed&Cmd=Search&Term="Jiang CZ"%5BAuthor%5D&itool=EntrezSystem2.PEntrez.Pubmed.Pubmed_ResultsPanel.Pubmed_RVAbstract)**,** [**Todo T**](http://www.ncbi.nlm.nih.gov/sites/entrez?Db=pubmed&Cmd=Search&Term="Todo T"%5BAuthor%5D&itool=EntrezSystem2.PEntrez.Pubmed.Pubmed_ResultsPanel.Pubmed_RVAbstract)**,** [**Britt AB**](http://www.ncbi.nlm.nih.gov/sites/entrez?Db=pubmed&Cmd=Search&Term="Britt AB"%5BAuthor%5D&itool=EntrezSystem2.PEntrez.Pubmed.Pubmed_ResultsPanel.Pubmed_RVAbstract)**,** [**Yamamoto K**](http://www.ncbi.nlm.nih.gov/sites/entrez?Db=pubmed&Cmd=Search&Term="Yamamoto K"%5BAuthor%5D&itool=EntrezSystem2.PEntrez.Pubmed.Pubmed_ResultsPanel.Pubmed_RVAbstract) (1998) Cloning and characterization of a gene (UVR3) required for photorepair of 6-4 photoproducts in Arabidopsis thaliana. Nucleic Acids Res **26(2):**638-644.

157.[**Chen IP**](http://www.ncbi.nlm.nih.gov/sites/entrez?Db=pubmed&Cmd=Search&Term="Chen IP"%5BAuthor%5D&itool=EntrezSystem2.PEntrez.Pubmed.Pubmed_ResultsPanel.Pubmed_DiscoveryPanel.Pubmed_RVAbstractPlus)**,** [**Mannuss A**](http://www.ncbi.nlm.nih.gov/sites/entrez?Db=pubmed&Cmd=Search&Term="Mannuss A"%5BAuthor%5D&itool=EntrezSystem2.PEntrez.Pubmed.Pubmed_ResultsPanel.Pubmed_DiscoveryPanel.Pubmed_RVAbstractPlus)**,** [**Orel N**](http://www.ncbi.nlm.nih.gov/sites/entrez?Db=pubmed&Cmd=Search&Term="Orel N"%5BAuthor%5D&itool=EntrezSystem2.PEntrez.Pubmed.Pubmed_ResultsPanel.Pubmed_DiscoveryPanel.Pubmed_RVAbstractPlus)**,** [**Heitzeberg F**](http://www.ncbi.nlm.nih.gov/sites/entrez?Db=pubmed&Cmd=Search&Term="Heitzeberg F"%5BAuthor%5D&itool=EntrezSystem2.PEntrez.Pubmed.Pubmed_ResultsPanel.Pubmed_DiscoveryPanel.Pubmed_RVAbstractPlus)**,** [**Puchta H**](http://www.ncbi.nlm.nih.gov/sites/entrez?Db=pubmed&Cmd=Search&Term="Puchta H"%5BAuthor%5D&itool=EntrezSystem2.PEntrez.Pubmed.Pubmed_ResultsPanel.Pubmed_DiscoveryPanel.Pubmed_RVAbstractPlus) (2008) A homolog of ScRAD5 is involved in DNA repair and homologous recombination in Arabidopsis. [Plant Physiol.](javascript:AL_get(this, 'jour', 'Plant Physiol.');) **146(4):**1786-1796.

158.**De Muyt A, Vezon D, Gendrot G, Gallois JL, Stevens R, Grelon M** (2007) AtPRD1 is required for meiotic double strand break formation in Arabidopsis thaliana. [EMBO J.](javascript:AL_get(this, 'jour', 'EMBO J.');) **26(18):**4126-4137.

159.[**Takeda S**](http://www.ncbi.nlm.nih.gov/sites/entrez?Db=pubmed&Cmd=Search&Term="Takeda S"%5BAuthor%5D&itool=EntrezSystem2.PEntrez.Pubmed.Pubmed_ResultsPanel.Pubmed_DiscoveryPanel.Pubmed_RVAbstractPlus)**,** [**Tadele Z**](http://www.ncbi.nlm.nih.gov/sites/entrez?Db=pubmed&Cmd=Search&Term="Tadele Z"%5BAuthor%5D&itool=EntrezSystem2.PEntrez.Pubmed.Pubmed_ResultsPanel.Pubmed_DiscoveryPanel.Pubmed_RVAbstractPlus)**,** [**Hofmann I**](http://www.ncbi.nlm.nih.gov/sites/entrez?Db=pubmed&Cmd=Search&Term="Hofmann I"%5BAuthor%5D&itool=EntrezSystem2.PEntrez.Pubmed.Pubmed_ResultsPanel.Pubmed_DiscoveryPanel.Pubmed_RVAbstractPlus)**,** [**Probst AV**](http://www.ncbi.nlm.nih.gov/sites/entrez?Db=pubmed&Cmd=Search&Term="Probst AV"%5BAuthor%5D&itool=EntrezSystem2.PEntrez.Pubmed.Pubmed_ResultsPanel.Pubmed_DiscoveryPanel.Pubmed_RVAbstractPlus)**,** [**Angelis KJ**](http://www.ncbi.nlm.nih.gov/sites/entrez?Db=pubmed&Cmd=Search&Term="Angelis KJ"%5BAuthor%5D&itool=EntrezSystem2.PEntrez.Pubmed.Pubmed_ResultsPanel.Pubmed_DiscoveryPanel.Pubmed_RVAbstractPlus)**,** [**Kaya H**](http://www.ncbi.nlm.nih.gov/sites/entrez?Db=pubmed&Cmd=Search&Term="Kaya H"%5BAuthor%5D&itool=EntrezSystem2.PEntrez.Pubmed.Pubmed_ResultsPanel.Pubmed_DiscoveryPanel.Pubmed_RVAbstractPlus)**,** [**Araki T**](http://www.ncbi.nlm.nih.gov/sites/entrez?Db=pubmed&Cmd=Search&Term="Araki T"%5BAuthor%5D&itool=EntrezSystem2.PEntrez.Pubmed.Pubmed_ResultsPanel.Pubmed_DiscoveryPanel.Pubmed_RVAbstractPlus)**,** [**Mengiste T**](http://www.ncbi.nlm.nih.gov/sites/entrez?Db=pubmed&Cmd=Search&Term="Mengiste T"%5BAuthor%5D&itool=EntrezSystem2.PEntrez.Pubmed.Pubmed_ResultsPanel.Pubmed_DiscoveryPanel.Pubmed_RVAbstractPlus)**,** [**Mittelsten Scheid O**](http://www.ncbi.nlm.nih.gov/sites/entrez?Db=pubmed&Cmd=Search&Term="Mittelsten Scheid O"%5BAuthor%5D&itool=EntrezSystem2.PEntrez.Pubmed.Pubmed_ResultsPanel.Pubmed_DiscoveryPanel.Pubmed_RVAbstractPlus)**,** [**Shibahara K**](http://www.ncbi.nlm.nih.gov/sites/entrez?Db=pubmed&Cmd=Search&Term="Shibahara K"%5BAuthor%5D&itool=EntrezSystem2.PEntrez.Pubmed.Pubmed_ResultsPanel.Pubmed_DiscoveryPanel.Pubmed_RVAbstractPlus)**,** [**Scheel D**](http://www.ncbi.nlm.nih.gov/sites/entrez?Db=pubmed&Cmd=Search&Term="Scheel D"%5BAuthor%5D&itool=EntrezSystem2.PEntrez.Pubmed.Pubmed_ResultsPanel.Pubmed_DiscoveryPanel.Pubmed_RVAbstractPlus)**,** [**Paszkowski J**](http://www.ncbi.nlm.nih.gov/sites/entrez?Db=pubmed&Cmd=Search&Term="Paszkowski J"%5BAuthor%5D&itool=EntrezSystem2.PEntrez.Pubmed.Pubmed_ResultsPanel.Pubmed_DiscoveryPanel.Pubmed_RVAbstractPlus) (2004) BRU1, a novel link between responses to DNA damage and epigenetic gene silencing in Arabidopsis. [Genes Dev.](javascript:AL_get(this, 'jour', 'Genes Dev.');) **18(7):**782-793.

160.**Suzuki T, Nakajima S, Inagaki S, Hirano-Nakakita M, Matsuoka K, Demura T, Fukuda H, Morikami A, Nakamura K** (2005) TONSOKU is expressed in S phase of the cell cycle and its defect delays cell cycle progression in Arabidopsis. Plant Cell Physiol. **46(5):**736-742.

161.[**Cenkci B**](http://www.ncbi.nlm.nih.gov/sites/entrez?Db=pubmed&Cmd=Search&Term="Cenkci B"%5BAuthor%5D&itool=EntrezSystem2.PEntrez.Pubmed.Pubmed_ResultsPanel.Pubmed_DiscoveryPanel.Pubmed_RVAbstractPlus)**,** [**Petersen JL**](http://www.ncbi.nlm.nih.gov/sites/entrez?Db=pubmed&Cmd=Search&Term="Petersen JL"%5BAuthor%5D&itool=EntrezSystem2.PEntrez.Pubmed.Pubmed_ResultsPanel.Pubmed_DiscoveryPanel.Pubmed_RVAbstractPlus)**,** [**Small GD**](http://www.ncbi.nlm.nih.gov/sites/entrez?Db=pubmed&Cmd=Search&Term="Small GD"%5BAuthor%5D&itool=EntrezSystem2.PEntrez.Pubmed.Pubmed_ResultsPanel.Pubmed_DiscoveryPanel.Pubmed_RVAbstractPlus) (2003)REX1, a novel gene required for DNA repair. [J Biol Chem.](javascript:AL_get(this, 'jour', 'J Biol Chem.');) **278(25):**22574-22577.

162.[**Cai X**](http://www.ncbi.nlm.nih.gov/sites/entrez?Db=pubmed&Cmd=Search&Term="Cai X"%5BAuthor%5D&itool=EntrezSystem2.PEntrez.Pubmed.Pubmed_ResultsPanel.Pubmed_RVAbstract)**,** [**Dong F**](http://www.ncbi.nlm.nih.gov/sites/entrez?Db=pubmed&Cmd=Search&Term="Dong F"%5BAuthor%5D&itool=EntrezSystem2.PEntrez.Pubmed.Pubmed_ResultsPanel.Pubmed_RVAbstract)**,** [**Edelmann RE**](http://www.ncbi.nlm.nih.gov/sites/entrez?Db=pubmed&Cmd=Search&Term="Edelmann RE"%5BAuthor%5D&itool=EntrezSystem2.PEntrez.Pubmed.Pubmed_ResultsPanel.Pubmed_RVAbstract)**,** [**Makaroff CA**](http://www.ncbi.nlm.nih.gov/sites/entrez?Db=pubmed&Cmd=Search&Term="Makaroff CA"%5BAuthor%5D&itool=EntrezSystem2.PEntrez.Pubmed.Pubmed_ResultsPanel.Pubmed_RVAbstract) (2003) The Arabidopsis SYN1 cohesin protein is required for sister chromatid arm cohesion and homologous chromosome pairing. [J Cell Sci.](javascript:AL_get(this, 'jour', 'J Cell Sci.');) **116**(Pt 14):2999-3007.

163.[**Lam WS**](http://www.ncbi.nlm.nih.gov/sites/entrez?Db=pubmed&Cmd=Search&Term="Lam WS"%5BAuthor%5D&itool=EntrezSystem2.PEntrez.Pubmed.Pubmed_ResultsPanel.Pubmed_RVAbstract)**,** [**Yang X**](http://www.ncbi.nlm.nih.gov/sites/entrez?Db=pubmed&Cmd=Search&Term="Yang X"%5BAuthor%5D&itool=EntrezSystem2.PEntrez.Pubmed.Pubmed_ResultsPanel.Pubmed_RVAbstract)**,** [**Makaroff CA**](http://www.ncbi.nlm.nih.gov/sites/entrez?Db=pubmed&Cmd=Search&Term="Makaroff CA"%5BAuthor%5D&itool=EntrezSystem2.PEntrez.Pubmed.Pubmed_ResultsPanel.Pubmed_RVAbstract) (2005)Characterization of Arabidopsis thaliana SMC1 and SMC3: evidence that AtSMC3 may function beyond chromosome cohesion. [J Cell Sci.](javascript:AL_get(this, 'jour', 'J Cell Sci.');) **118**(Pt 14):3037-48.

164.**Siddiqui NU, Rusyniak S, Hasenkampf CA, Riggs CD** (2006) Disruption of the Arabidopsis SMC4 gene, AtCAP-C, compromises gametogenesis and embryogenesis. Planta **223(5):**990-997.

165.[**Hong SH**](http://www.ncbi.nlm.nih.gov/sites/entrez?Db=pubmed&Cmd=Search&Term="Hong SH"%5BAuthor%5D&itool=EntrezSystem2.PEntrez.Pubmed.Pubmed_ResultsPanel.Pubmed_RVAbstract)**,** [**Kim HJ**](http://www.ncbi.nlm.nih.gov/sites/entrez?Db=pubmed&Cmd=Search&Term="Kim HJ"%5BAuthor%5D&itool=EntrezSystem2.PEntrez.Pubmed.Pubmed_ResultsPanel.Pubmed_RVAbstract)**,** [**Ryu JS**](http://www.ncbi.nlm.nih.gov/sites/entrez?Db=pubmed&Cmd=Search&Term="Ryu JS"%5BAuthor%5D&itool=EntrezSystem2.PEntrez.Pubmed.Pubmed_ResultsPanel.Pubmed_RVAbstract)**,** [**Choi H**](http://www.ncbi.nlm.nih.gov/sites/entrez?Db=pubmed&Cmd=Search&Term="Choi H"%5BAuthor%5D&itool=EntrezSystem2.PEntrez.Pubmed.Pubmed_ResultsPanel.Pubmed_RVAbstract)**,** [**Jeong S**](http://www.ncbi.nlm.nih.gov/sites/entrez?Db=pubmed&Cmd=Search&Term="Jeong S"%5BAuthor%5D&itool=EntrezSystem2.PEntrez.Pubmed.Pubmed_ResultsPanel.Pubmed_RVAbstract)**,** [**Shin J**](http://www.ncbi.nlm.nih.gov/sites/entrez?Db=pubmed&Cmd=Search&Term="Shin J"%5BAuthor%5D&itool=EntrezSystem2.PEntrez.Pubmed.Pubmed_ResultsPanel.Pubmed_RVAbstract)**,** [**Choi G**](http://www.ncbi.nlm.nih.gov/sites/entrez?Db=pubmed&Cmd=Search&Term="Choi G"%5BAuthor%5D&itool=EntrezSystem2.PEntrez.Pubmed.Pubmed_ResultsPanel.Pubmed_RVAbstract)**,** [**Nam HG**](http://www.ncbi.nlm.nih.gov/sites/entrez?Db=pubmed&Cmd=Search&Term="Nam HG"%5BAuthor%5D&itool=EntrezSystem2.PEntrez.Pubmed.Pubmed_ResultsPanel.Pubmed_RVAbstract) (2008) CRY1 inhibits COP1-mediated degradation of BIT1, a MYB transcription factor, to activate blue light-dependent gene expression in Arabidopsis. [Plant J.](javascript:AL_get(this, 'jour', 'Plant J.');) **55(3):**361-371.

166.[**Yu X**](http://www.ncbi.nlm.nih.gov/sites/entrez?Db=pubmed&Cmd=Search&Term="Yu X"%5BAuthor%5D&itool=EntrezSystem2.PEntrez.Pubmed.Pubmed_ResultsPanel.Pubmed_RVAbstract)**,** [**Sayegh R**](http://www.ncbi.nlm.nih.gov/sites/entrez?Db=pubmed&Cmd=Search&Term="Sayegh R"%5BAuthor%5D&itool=EntrezSystem2.PEntrez.Pubmed.Pubmed_ResultsPanel.Pubmed_RVAbstract)**,** [**Maymon M**](http://www.ncbi.nlm.nih.gov/sites/entrez?Db=pubmed&Cmd=Search&Term="Maymon M"%5BAuthor%5D&itool=EntrezSystem2.PEntrez.Pubmed.Pubmed_ResultsPanel.Pubmed_RVAbstract)**,** [**Warpeha K**](http://www.ncbi.nlm.nih.gov/sites/entrez?Db=pubmed&Cmd=Search&Term="Warpeha K"%5BAuthor%5D&itool=EntrezSystem2.PEntrez.Pubmed.Pubmed_ResultsPanel.Pubmed_RVAbstract)**,** [**Klejnot J**](http://www.ncbi.nlm.nih.gov/sites/entrez?Db=pubmed&Cmd=Search&Term="Klejnot J"%5BAuthor%5D&itool=EntrezSystem2.PEntrez.Pubmed.Pubmed_ResultsPanel.Pubmed_RVAbstract)**,** [**Yang H**](http://www.ncbi.nlm.nih.gov/sites/entrez?Db=pubmed&Cmd=Search&Term="Yang H"%5BAuthor%5D&itool=EntrezSystem2.PEntrez.Pubmed.Pubmed_ResultsPanel.Pubmed_RVAbstract)**,** [**Huang J**](http://www.ncbi.nlm.nih.gov/sites/entrez?Db=pubmed&Cmd=Search&Term="Huang J"%5BAuthor%5D&itool=EntrezSystem2.PEntrez.Pubmed.Pubmed_ResultsPanel.Pubmed_RVAbstract)**,** [**Lee J**](http://www.ncbi.nlm.nih.gov/sites/entrez?Db=pubmed&Cmd=Search&Term="Lee J"%5BAuthor%5D&itool=EntrezSystem2.PEntrez.Pubmed.Pubmed_ResultsPanel.Pubmed_RVAbstract)**,** [**Kaufman L**](http://www.ncbi.nlm.nih.gov/sites/entrez?Db=pubmed&Cmd=Search&Term="Kaufman L"%5BAuthor%5D&itool=EntrezSystem2.PEntrez.Pubmed.Pubmed_ResultsPanel.Pubmed_RVAbstract)**,** [**Lin C**](http://www.ncbi.nlm.nih.gov/sites/entrez?Db=pubmed&Cmd=Search&Term="Lin C"%5BAuthor%5D&itool=EntrezSystem2.PEntrez.Pubmed.Pubmed_ResultsPanel.Pubmed_RVAbstract) (2009) Formation of nuclear bodies of Arabidopsis CRY2 in response to blue light is associated with its blue light-dependent degradation. [Plant Cell](javascript:AL_get(this, 'jour', 'Plant Cell.');) **21(1):**118-130.

167.[**Onda Y**](http://www.ncbi.nlm.nih.gov/sites/entrez?Db=pubmed&Cmd=Search&Term="Onda Y"%5BAuthor%5D&itool=EntrezSystem2.PEntrez.Pubmed.Pubmed_ResultsPanel.Pubmed_RVAbstract)**,** [**Yagi Y**](http://www.ncbi.nlm.nih.gov/sites/entrez?Db=pubmed&Cmd=Search&Term="Yagi Y"%5BAuthor%5D&itool=EntrezSystem2.PEntrez.Pubmed.Pubmed_ResultsPanel.Pubmed_RVAbstract)**,** [**Saito Y**](http://www.ncbi.nlm.nih.gov/sites/entrez?Db=pubmed&Cmd=Search&Term="Saito Y"%5BAuthor%5D&itool=EntrezSystem2.PEntrez.Pubmed.Pubmed_ResultsPanel.Pubmed_RVAbstract)**,** [**Takenaka N**](http://www.ncbi.nlm.nih.gov/sites/entrez?Db=pubmed&Cmd=Search&Term="Takenaka N"%5BAuthor%5D&itool=EntrezSystem2.PEntrez.Pubmed.Pubmed_ResultsPanel.Pubmed_RVAbstract)**,** [**Toyoshima Y**](http://www.ncbi.nlm.nih.gov/sites/entrez?Db=pubmed&Cmd=Search&Term="Toyoshima Y"%5BAuthor%5D&itool=EntrezSystem2.PEntrez.Pubmed.Pubmed_ResultsPanel.Pubmed_RVAbstract) (2008) Light induction of Arabidopsis SIG1 and SIG5 transcripts in mature leaves: differential roles of cryptochrome 1 and cryptochrome 2 and dual function of SIG5 in the recognition of plastid promoters. [Plant J.](javascript:AL_get(this, 'jour', 'Plant J.');) **55(6):**968-978.

168.[Liu H](http://www.ncbi.nlm.nih.gov/sites/entrez?Db=pubmed&Cmd=Search&Term="Liu H"%5BAuthor%5D&itool=EntrezSystem2.PEntrez.Pubmed.Pubmed_ResultsPanel.Pubmed_RVAbstract), [Yu X](http://www.ncbi.nlm.nih.gov/sites/entrez?Db=pubmed&Cmd=Search&Term="Yu X"%5BAuthor%5D&itool=EntrezSystem2.PEntrez.Pubmed.Pubmed_ResultsPanel.Pubmed_RVAbstract), [Li K](http://www.ncbi.nlm.nih.gov/sites/entrez?Db=pubmed&Cmd=Search&Term="Li K"%5BAuthor%5D&itool=EntrezSystem2.PEntrez.Pubmed.Pubmed_ResultsPanel.Pubmed_RVAbstract), [Klejnot J](http://www.ncbi.nlm.nih.gov/sites/entrez?Db=pubmed&Cmd=Search&Term="Klejnot J"%5BAuthor%5D&itool=EntrezSystem2.PEntrez.Pubmed.Pubmed_ResultsPanel.Pubmed_RVAbstract), [Yang H](http://www.ncbi.nlm.nih.gov/sites/entrez?Db=pubmed&Cmd=Search&Term="Yang H"%5BAuthor%5D&itool=EntrezSystem2.PEntrez.Pubmed.Pubmed_ResultsPanel.Pubmed_RVAbstract), [Lisiero D](http://www.ncbi.nlm.nih.gov/sites/entrez?Db=pubmed&Cmd=Search&Term="Lisiero D"%5BAuthor%5D&itool=EntrezSystem2.PEntrez.Pubmed.Pubmed_ResultsPanel.Pubmed_RVAbstract), [Lin C](http://www.ncbi.nlm.nih.gov/sites/entrez?Db=pubmed&Cmd=Search&Term="Lin C"%5BAuthor%5D&itool=EntrezSystem2.PEntrez.Pubmed.Pubmed_ResultsPanel.Pubmed_RVAbstract). (2008) Photoexcited CRY2 interacts with CIB1 to regulate transcription and floral initiation in Arabidopsis. [Science.](javascript:AL_get(this, 'jour', 'Science.');) 322(5907):1535-1539

169.[**Klar T**](http://www.ncbi.nlm.nih.gov/sites/entrez?Db=pubmed&Cmd=Search&Term="Klar T"%5BAuthor%5D&itool=EntrezSystem2.PEntrez.Pubmed.Pubmed_ResultsPanel.Pubmed_RVAbstract)**,** [**Pokorny R**](http://www.ncbi.nlm.nih.gov/sites/entrez?Db=pubmed&Cmd=Search&Term="Pokorny R"%5BAuthor%5D&itool=EntrezSystem2.PEntrez.Pubmed.Pubmed_ResultsPanel.Pubmed_RVAbstract)**,** [**Moldt J**](http://www.ncbi.nlm.nih.gov/sites/entrez?Db=pubmed&Cmd=Search&Term="Moldt J"%5BAuthor%5D&itool=EntrezSystem2.PEntrez.Pubmed.Pubmed_ResultsPanel.Pubmed_RVAbstract)**,** [**Batschauer A**](http://www.ncbi.nlm.nih.gov/sites/entrez?Db=pubmed&Cmd=Search&Term="Batschauer A"%5BAuthor%5D&itool=EntrezSystem2.PEntrez.Pubmed.Pubmed_ResultsPanel.Pubmed_RVAbstract)**,** [**Essen LO**](http://www.ncbi.nlm.nih.gov/sites/entrez?Db=pubmed&Cmd=Search&Term="Essen LO"%5BAuthor%5D&itool=EntrezSystem2.PEntrez.Pubmed.Pubmed_ResultsPanel.Pubmed_RVAbstract) (2007) Cryptochrome 3 from Arabidopsis thaliana: structural and functional analysis of its complex with a folate light antenna. [J Mol Biol.](javascript:AL_get(this, 'jour', 'J Mol Biol.');) **366(3):**954-64.

170.[**Huang Y**](http://www.ncbi.nlm.nih.gov/sites/entrez?Db=pubmed&Cmd=Search&Term="Huang Y"%5BAuthor%5D&itool=EntrezSystem2.PEntrez.Pubmed.Pubmed_ResultsPanel.Pubmed_RVAbstract)**,** [**Baxter R**](http://www.ncbi.nlm.nih.gov/sites/entrez?Db=pubmed&Cmd=Search&Term="Baxter R"%5BAuthor%5D&itool=EntrezSystem2.PEntrez.Pubmed.Pubmed_ResultsPanel.Pubmed_RVAbstract)**,** [**Smith BS**](http://www.ncbi.nlm.nih.gov/sites/entrez?Db=pubmed&Cmd=Search&Term="Smith BS"%5BAuthor%5D&itool=EntrezSystem2.PEntrez.Pubmed.Pubmed_ResultsPanel.Pubmed_RVAbstract)**,** [**Partch CL**](http://www.ncbi.nlm.nih.gov/sites/entrez?Db=pubmed&Cmd=Search&Term="Partch CL"%5BAuthor%5D&itool=EntrezSystem2.PEntrez.Pubmed.Pubmed_ResultsPanel.Pubmed_RVAbstract)**,** [**Colbert CL**](http://www.ncbi.nlm.nih.gov/sites/entrez?Db=pubmed&Cmd=Search&Term="Colbert CL"%5BAuthor%5D&itool=EntrezSystem2.PEntrez.Pubmed.Pubmed_ResultsPanel.Pubmed_RVAbstract)**,** [**Deisenhofer J**](http://www.ncbi.nlm.nih.gov/sites/entrez?Db=pubmed&Cmd=Search&Term="Deisenhofer J"%5BAuthor%5D&itool=EntrezSystem2.PEntrez.Pubmed.Pubmed_ResultsPanel.Pubmed_RVAbstract) (2006) Crystal structure of cryptochrome 3 from Arabidopsis thaliana and its implications for photolyase activity. [Proc Natl Acad Sci U S A.](javascript:AL_get(this, 'jour', 'Proc Natl Acad Sci U S A.');) **103(47):**17701-17706.

171.[**Hoffman PD**](http://www.ncbi.nlm.nih.gov/sites/entrez?Db=pubmed&Cmd=Search&Term="Hoffman PD"%5BAuthor%5D&itool=EntrezSystem2.PEntrez.Pubmed.Pubmed_ResultsPanel.Pubmed_RVAbstract)**,** [**Batschauer A**](http://www.ncbi.nlm.nih.gov/sites/entrez?Db=pubmed&Cmd=Search&Term="Batschauer A"%5BAuthor%5D&itool=EntrezSystem2.PEntrez.Pubmed.Pubmed_ResultsPanel.Pubmed_RVAbstract)**,** [**Hays JB**](http://www.ncbi.nlm.nih.gov/sites/entrez?Db=pubmed&Cmd=Search&Term="Hays JB"%5BAuthor%5D&itool=EntrezSystem2.PEntrez.Pubmed.Pubmed_ResultsPanel.Pubmed_RVAbstract) (1996) PHH1, a novel gene from Arabidopsis thaliana that encodes a protein similar to plant blue-light photoreceptors and microbial photolyases. [Mol Gen Genet.](javascript:AL_get(this, 'jour', 'Mol Gen Genet.');) **253(1-2):**259-265.

172.[**Landry LG**](http://www.ncbi.nlm.nih.gov/sites/entrez?Db=pubmed&Cmd=Search&Term="Landry LG"%5BAuthor%5D&itool=EntrezSystem2.PEntrez.Pubmed.Pubmed_ResultsPanel.Pubmed_RVAbstract)**,** [**Stapleton AE**](http://www.ncbi.nlm.nih.gov/sites/entrez?Db=pubmed&Cmd=Search&Term="Stapleton AE"%5BAuthor%5D&itool=EntrezSystem2.PEntrez.Pubmed.Pubmed_ResultsPanel.Pubmed_RVAbstract)**,** [**Lim J**](http://www.ncbi.nlm.nih.gov/sites/entrez?Db=pubmed&Cmd=Search&Term="Lim J"%5BAuthor%5D&itool=EntrezSystem2.PEntrez.Pubmed.Pubmed_ResultsPanel.Pubmed_RVAbstract)**,** [**Hoffman P**](http://www.ncbi.nlm.nih.gov/sites/entrez?Db=pubmed&Cmd=Search&Term="Hoffman P"%5BAuthor%5D&itool=EntrezSystem2.PEntrez.Pubmed.Pubmed_ResultsPanel.Pubmed_RVAbstract)**,** [**Hays JB**](http://www.ncbi.nlm.nih.gov/sites/entrez?Db=pubmed&Cmd=Search&Term="Hays JB"%5BAuthor%5D&itool=EntrezSystem2.PEntrez.Pubmed.Pubmed_ResultsPanel.Pubmed_RVAbstract)**,** [**Walbot V**](http://www.ncbi.nlm.nih.gov/sites/entrez?Db=pubmed&Cmd=Search&Term="Walbot V"%5BAuthor%5D&itool=EntrezSystem2.PEntrez.Pubmed.Pubmed_ResultsPanel.Pubmed_RVAbstract)**,** [**Last RL**](http://www.ncbi.nlm.nih.gov/sites/entrez?Db=pubmed&Cmd=Search&Term="Last RL"%5BAuthor%5D&itool=EntrezSystem2.PEntrez.Pubmed.Pubmed_ResultsPanel.Pubmed_RVAbstract) (1997)An Arabidopsis photolyase mutant is hypersensitive to ultraviolet-B radiation. [Proc Natl Acad Sci U S A.](javascript:AL_get(this, 'jour', 'Proc Natl Acad Sci U S A.');) **94(1):**328-332. PHR1

173. **Jarillo JA,Ahmad M, Cashmore AR** (1998). *PHR2*: A Novel Arabidopsis gene related to the blue-light photoreceptor/photolyase family. Plant Physiol. **117**, 719.

174. **Li J, Wen TJ, Schnable PS** (2008) Role of RAD51 in the repair of MuDR-induced double-strand breaks in maize (Zea mays L.).Genetics **178(1):**57-66.

175. **Li J, Harper LC, Golubovskaya I, Wang CR, Weber D, Meeley RB, McElver J, Bowen B, Cande WZ, Schnable PS** (2007) Functional analysis of maize RAD51 in meiosis and double-strand break repair.Genetics. **176(3):**1469-1482.

176. **Zuo Z, Mahajan PB** (2005) Recombinant expression of maize nucleotide excision repair protein Rad23 in Escherichia coli.Protein Expr Purif. **41(2)**:287-297.

177. **Horwath M, Kramer W, Kunze R** (2002)Structure and expression of the Zea mays mutS-homologs Mus1 and Mus2.Theor Appl Genet. **105(2-3)**:423-430.

178**. Lloyd AH, Milligan AS, Langridge P, Able JA** (2007)TaMSH7: a cereal mismatch repair gene that affects fertility in transgenic barley (Hordeum vulgare L.).BMC Plant Biol. **7**:67.

179.**de Bustos A, Pérez R, Jouve N** (2007)Characterization of the gene Mre11 and evidence of silencing after polyploidization in Triticum.Theor Appl Genet. **114(6):**985-999.

180. **Scortecci KC, Lima AF, Carvalho FM, Silva UB, Agnez-Lima LF, Batistuzzo de Medeiros SR** (2007) A characterization of a MutM/Fpg ortholog in sugarcane--A monocot plant.Biochem Biophys Res Commun.**361(4)**:1054-60.

181. **Sullivan ML, Carpenter TB, Vierstra RD** (1994)Homologues of wheat ubiquitin-conjugating enzymes--TaUBC1 and TaUBC4 are encoded by small multigene families in Arabidopsis thaliana.Plant Mol Biol. **24(4)**:651-61.

182. **Sullivan ML, Vierstra RD**(1989)A ubiquitin carrier protein from wheat germ is structurally and functionally similar to the yeast DNA repair enzyme encoded by RAD6.Proc Natl Acad Sci U S A.**86(24):**9861-9865.

183. **van der Knaap E, Jagoueix S, Kende H** (1997)Expression of an ortholog of replication protein A1 (RPA1) is induced by gibberellin in deepwater rice.Proc Natl Acad Sci U S A. **94(18):**9979-9983.

184. **Ishibashi T, Kimura S, Furukawa T, Hatanaka M, Hashimoto J, Sakaguchi K** (2001)Two types of replication protein A 70 kDa subunit in rice, Oryza sativa: molecular cloning, characterization, and cellular & tissue distribution.Gene.**272(1-2):**335-343.

185. **Kimura S, Furukawa T, Kasai N, Mori Y, Kitamoto HK, Sugawara F, Hashimoto J, Sakaguchi K**(2003)Functional characterization of two flap endonuclease-1 homologues in rice.Gene.**314**:63-71.

186. **Yamamoto T, Mori Y, Ishibashi T, Uchiyama Y, Sakaguchi N, Furukawa T, Hashimoto J, Kimura S, Sakaguchi K** (2004) Characterization of Rad6 from a higher plant, rice (Oryza sativa L.) and its interaction with Sgt1, a subunit of the SCF ubiquitin ligase complex.Biochem Biophys Res Commun. **314(2):**434-439.

187. **Rajanikant C, Kumbhakar M, Pal H, Rao BJ, Sainis JK** (2006) DNA strand exchange activity of rice recombinase OsDmc1 monitored by fluorescence resonance energy transfer and the role of ATP hydrolysis.FEBS J.273(7):1497-1506.

188. **Yoo, S-C., Cho, S-H., Sugimoto, H., Li, Jinjie., Kusumi, K., Koh, H-J., Iba, K. and Paek, N-C** (2009) Rice *Virescent-3* and *Stripe-1* encoding the large and small subunits of ribonucleotide reductase are required for chloroplast biogenesis during early leaf development. Plant Physiol*.* **150**:388-401.

189. **Philipps G, Clément B, Gigot C** (1995) Molecular characterization and cell cycle-regulated expression of a cDNA clone from *Arabidopsis thaliana* homologous to the small subunit of ribonucleotide reductase. FEBS Lett **358**: 67–70.

190.**Chabouté ME, Combettes B, Clément B, Gigot C, Philipps G** (1998) Molecular characterization of tobacco ribonucleotide reductase RNR1 and RNR2 cDNAs and cell cycle-regulated expression in synchronized plant cells. Plant Mol Biol **38**: 797–806.

191.**Elleingand E, Gerez C, Un S, Knüpling M, Lu G, Salem J, Rubin H, Sauge-Merle S, Laulhére JP, Fontecave M** (1998) Reactivity studies of the tyrosyl radical in ribonucleotide reductase from *Mycobacterium tuberculosis* and *Arabidopsis thaliana*: comparison with *Escherichia coli* and mouse. Eur J Biochem **258**: 485–490

192. **Sauge-Merle S, Falconet D, Fontecave M** (1999) An active ribonucleotide reductase from *Arabidopsis thaliana* cloning, expression and characterization of the large subunit. Eur J Biochem **266**: 62–69.

193.**Garton S, Knight H, Warren GJ, Knight MR, Thorlby GJ** (2007) *crinkled leaves 8*—a mutation in the large subunit of ribonucleotide reductase—leads to defects in leaf development and chloroplast division in *Arabidopsis thaliana*. Plant J **50**: 118–127.

194. **Liu CM, Meinke DW** (1998). The titan mutants of *Arabidopsis* are disrupted in mitosis and cell cycle control during seed development. Plant J. **16**: 21-31.

195. **Liu CM, McElver J, Tzafrir I, Joosen R, Wittich P, Patton D, van Lammeren AA Meinke D** (2002). Condensin and cohesin knockouts in *Arabidopsis* exhibit a *titan* seed phenotype. Plant J. **29**: 405-415.

196. **Siddiqui NU, Stronghill PE, Dengler RE, Hasenkampf CA, Riggs CD** (2003) Mutations in Arabidopsis condensin genes disrupt embryogenesis, meristem organization and segregation of homologous chromosomes during meiosis..Development. **130(14):**3283-3295.

197. **Pepper A, Chory J** (1997) Extragenic suppressors of the Arabidopsis detl mutant identify elements of flowering-time and lightresponse regulatory pathways. Genetics **145**: 1125–1137.

198.**Chory J, Peto C, Feinbaum R, Pratt L , Ausubel F** (1989) Arabidopsis thaliana mutant that develops as a light-grown plant in the absence of light. Cell **58**: 991–999.

199. **Chory J, Peto C** (1990) Mutations in the DET1 gene affect celltype-specific expression of light regulated genes and chloroplast development in Arabidopsis. Proc. Natl. Acad. Sci. USA **87**: 8776–8780.

200. **Couteau F, Belzile F, Horlow C, Grandjean O, Vezon D, Doutriaux MP**(1999) Random chromosome segregation without meiotic arrest in both male and female meiocytes of a dmc1 mutant of Arabidopsis. Plant Cell. **11(9)**:1623-1634.

201. **Wang S, Liu J, Feng Y, Niu X, Giovannoni J, Liu Y** (2008)Altered plastid levels and potential for improved fruit nutrient content by downregulation of the tomato DDB1-interacting protein CUL4. Plant J. **55(1):**89-103.

202. **Toueille M, Saint-Jean B, Rome C, Couillaud F, Castroviejo M, Benedetto JP** (2002) Two distinct proliferating cell nuclear antigens are present in the wheat cell Plant Physiol. Biochem. **40**: 743-748.

203**. Z-Y Deng, T Wang** (2007) OsDMC1 is required for homologous pairing in Oryza sativa. Plant mol. Biol**. 65(1-2):** 31-42.

204. **Jenik PD, Jurkuta RE, Barton MK** (2005) Interactions between the cell cycle and embryonic patterning in Arabidopsis uncovered by a mutation in DNA polymerase epsilon. Plant Cell **17**: 3362–3377.

205. **Ronceret A, Guilleminot J, Lincker F, Gadea-Vacas J, Delorme V, Bechtold N, Pelletier G, Delseny M, Chaboute ME, Devic M** (2005) Genetic analysis of two Arabidopsis DNA polymerase epsilon subunits during early embryogenesis. Plant J **44**: 223–236.

206. **Pierre Boesch, Noha Ibrahim, François Paulus, Anne Cosset, Vladislav Tarasenko and André Dietrich** (2009) Plant mitochondria possess a short-patch base excision DNA repair pathway. Nucleic Acids Res, doi:10.1093/nar/gkp606
