# Supplementary figures and images for: DNA repair and recombination in higher plants: insights from comparative genomics of arabidopsis and rice
Source: BMC Genomics. 2010 Jul 21;11:443. doi: 10.1186/1471-2164-11-443 (PMC3091640; doi:10.1186/1471-2164-11-443)

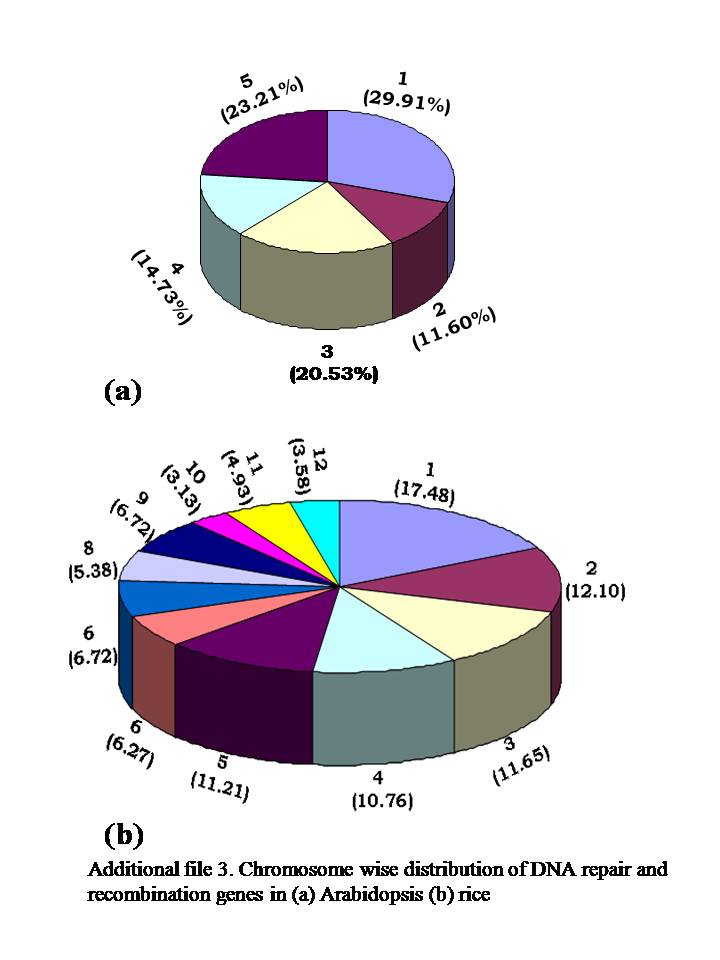

Supplement: Additional file 3 — Chromosome wise distribution of DRR genes in (a) Arabidopsis (b) rice. Values in brackets are in percentage. [file 1471-2164-11-443-S3.JPEG]
