# Supplementary material for: DNA repair and recombination in higher plants: insights from comparative genomics of arabidopsis and rice
Source: BMC Genomics. 2010 Jul 21;11:443. doi: 10.1186/1471-2164-11-443 (PMC3091640; doi:10.1186/1471-2164-11-443)
Supplement: Additional file 4 — Location of DRR genes on different chromosome of Arabidopsis by (a) their function in different pathway and (b) by their TAIR accession number. [file 1471-2164-11-443-S4.PDF]

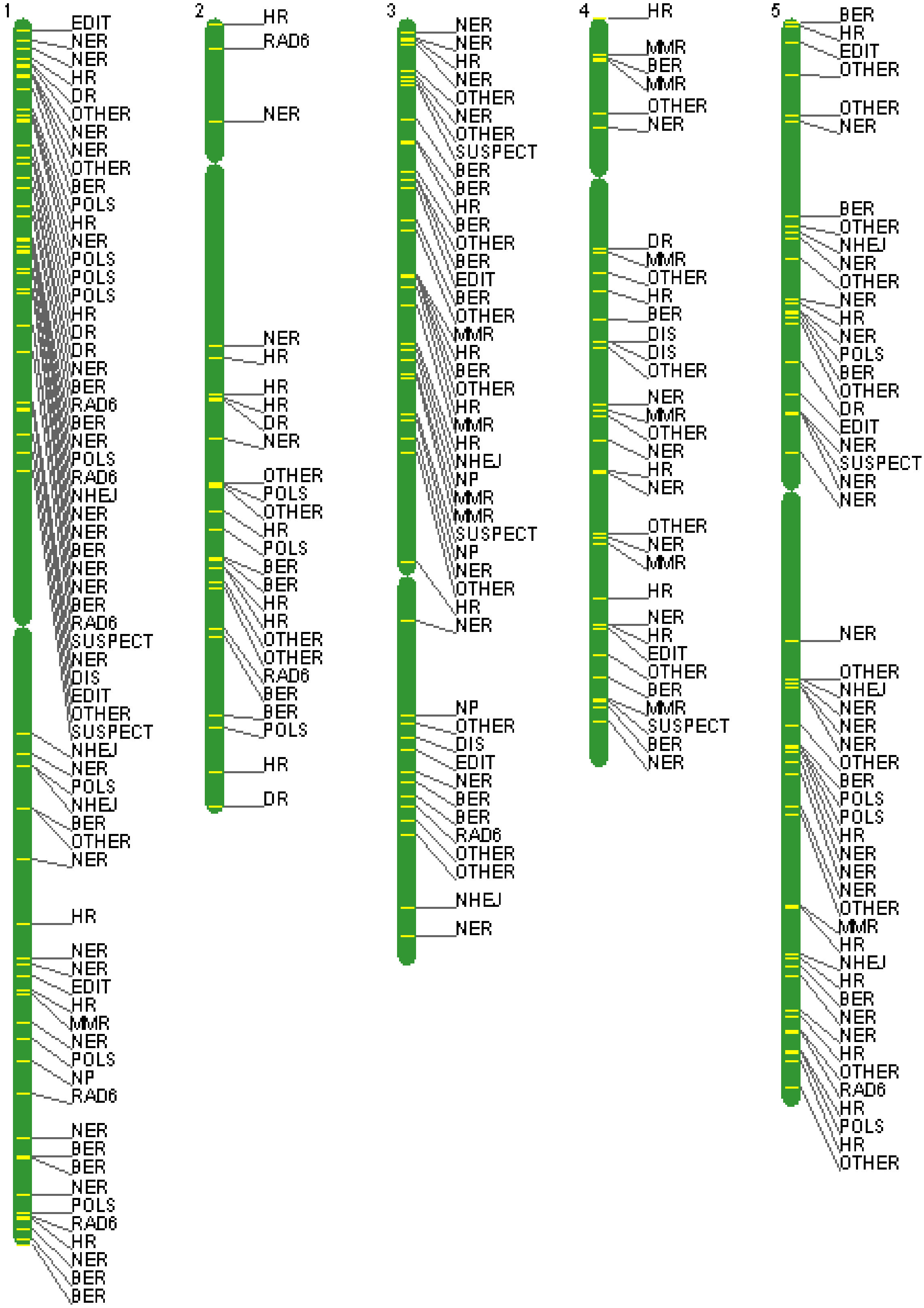
1
EDIT
NER
NER
HR
DR
OTHER
NER
NER
OTHER
BER
POLS
HR
NER
POLS
POLS
POLS
HR
DR
DR
NER
BER
RAD6
BER
NER
POLS
RAD6
NHEJ
NER
NER
BER
NER
NER
BER
RAD6
SUSPECT
NER
DIS
EDIT
OTHER
SUSPECT
NHEJ
NER
POLS
NHEJ
BER
OTHER
NER
HR
NER
NER
EDIT
HR
MMR
NER
POLS
NP
RAD6
NER
BER
BER
NER
POLS
RAD6
HR
NER
BER
BER
2
HR
RAD6
NER
NER
HR
HR
HR
DR
NER
OTHER
POLS
OTHER
HR
POLS
BER
BER
HR
HR
OTHER
OTHER
RAD6
BER
BER
POLS
HR
DR
3
NER
NER
HR
NER
OTHER
NER
OTHER
SUSPECT
BER
BER
HR
BER
OTHER
BER
EDIT
BER
OTHER
MMR
HR
BER
OTHER
HR
MMR
HR
NHEJ
NP
MMR
MMR
SUSPECT
NP
NER
OTHER
HR
NER
NP
OTHER
DIS
EDIT
NER
BER
BER
RAD6
OTHER
OTHER
NHEJ
NER
4
HR
MMR
BER
MMR
OTHER
NER
DR
MMR
OTHER
HR
BER
DIS
DIS
OTHER
NER
MMR
OTHER
NER
HR
NER
OTHER
NER
MMR
HR
NER
HR
EDIT
OTHER
BER
MMR
SUSPECT
BER
NER
5
BER
HR
EDIT
OTHER
OTHER
NER
BER
OTHER
NHEJ
NER
OTHER
NER
HR
NER
POLS
BER
OTHER
DR
EDIT
NER
SUSPECT
NER
NER
NER
OTHER
NHEJ
NER
NER
NER
OTHER
BER
POLS
POLS
HR
NER
NER
NER
OTHER
MMR
HR
NHEJ
HR
BER
NER
NER
HR
OTHER
RAD6
HR
POLS
HR
OTHER

(a)

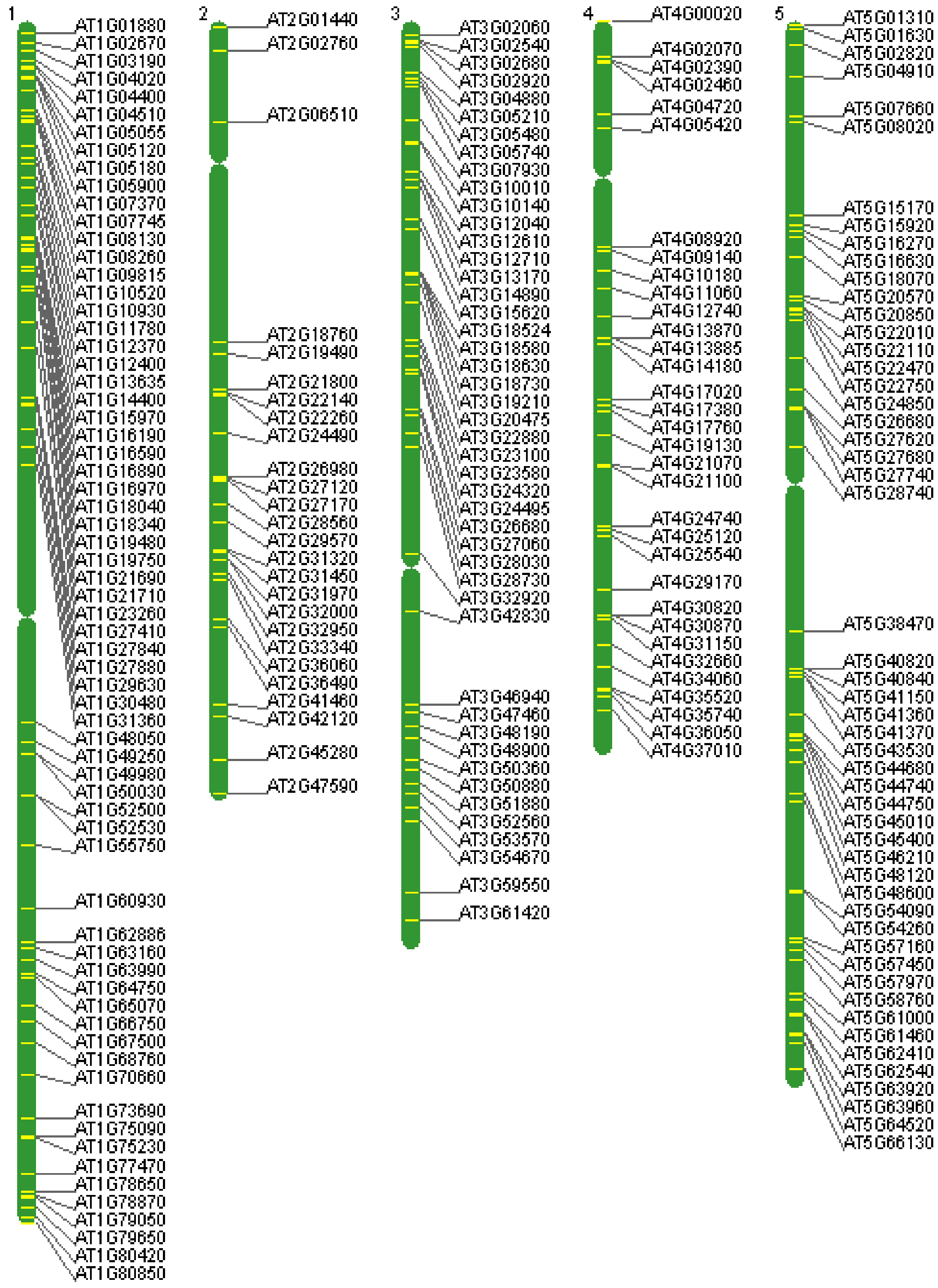

(b)

Additional file 4 Location of DNA repair and recombination genes on different chromosome of Arabidopsis by (a) their function in different pathway and (b) by their TAIR accession number
