# Supplementary material for: DNA repair and recombination in higher plants: insights from comparative genomics of arabidopsis and rice
Source: BMC Genomics. 2010 Jul 21;11:443. doi: 10.1186/1471-2164-11-443 (PMC3091640; doi:10.1186/1471-2164-11-443)
Supplement: Additional file 5 — Gene structure of the intragenomic duplicated DRR gene in Arabidopsis. [file 1471-2164-11-443-S5.PPT]

## Slide 1
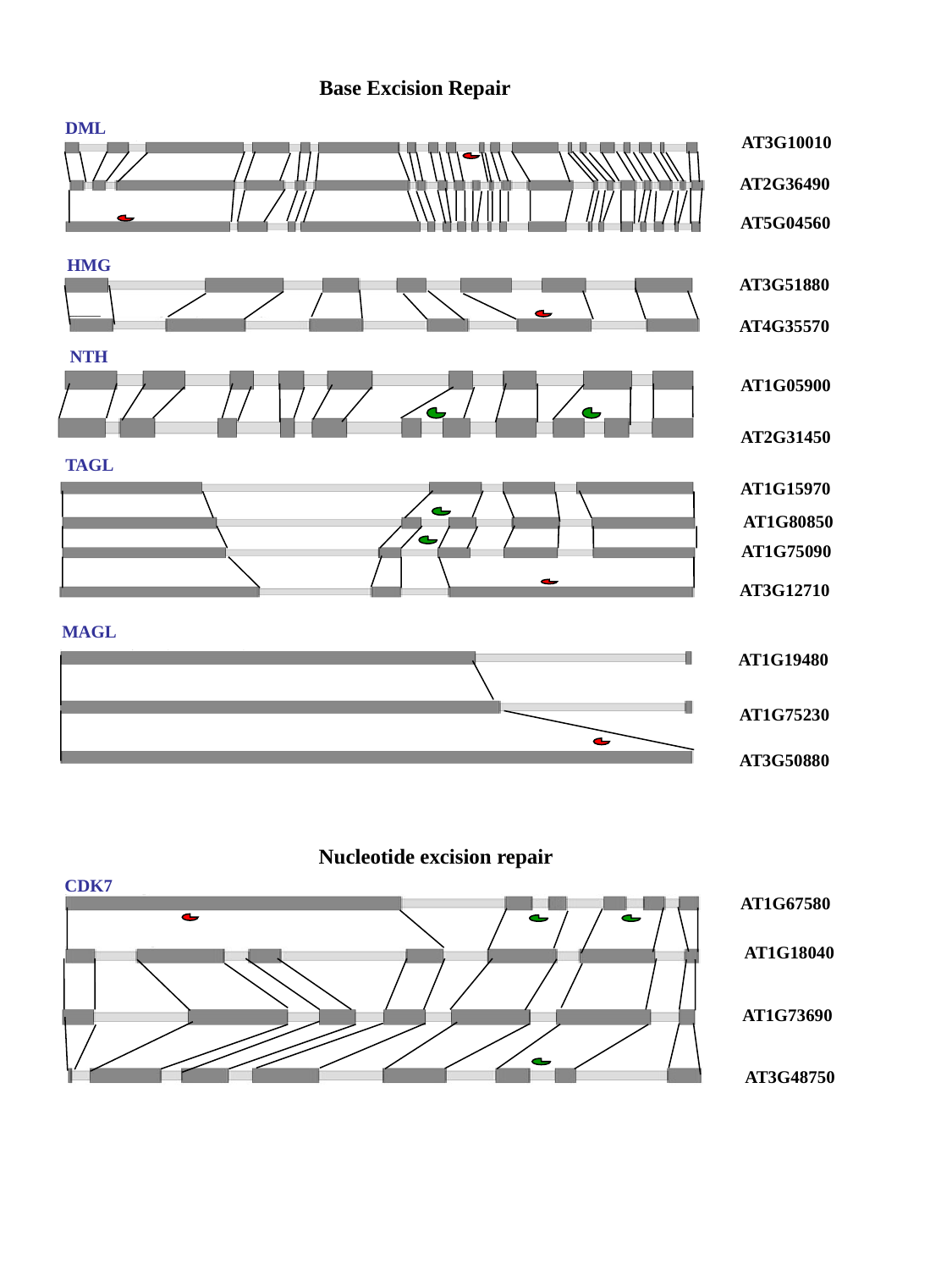

Base Excision Repair
DML
AT3G10010
AT2G36490
AT5G04560
HMG
AT3G51880
AT4G35570
NTH
AT1G05900
AT2G31450
TAGL
AT1G15970
AT1G80850
AT1G75090
AT3G12710
MAGL
AT1G19480
AT1G75230
AT3G50880
Nucleotide excision repair
CDK7
AT1G67580
AT1G18040
AT1G73690
AT3G48750

## Slide 2
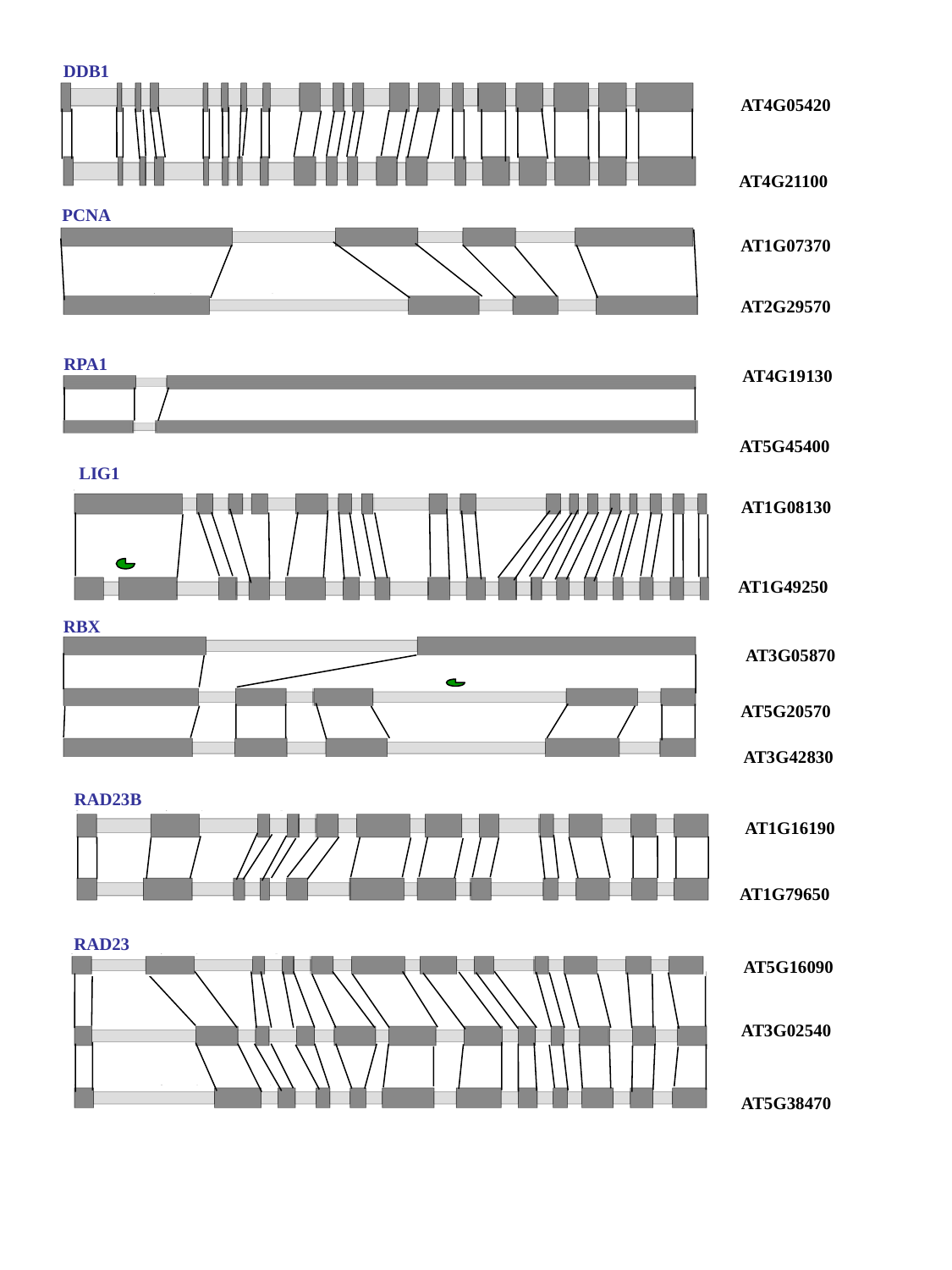

DDB1
AT4G05420
AT4G21100
PCNA
AT1G07370
AT2G29570
RPA1
AT4G19130
AT5G45400
LIG1
AT1G08130
AT1G49250
RBX
AT3G05870
AT5G20570
AT3G42830
RAD23B
AT1G16190
AT1G79650
RAD23
AT5G16090
AT3G02540
AT5G38470

## Slide 3
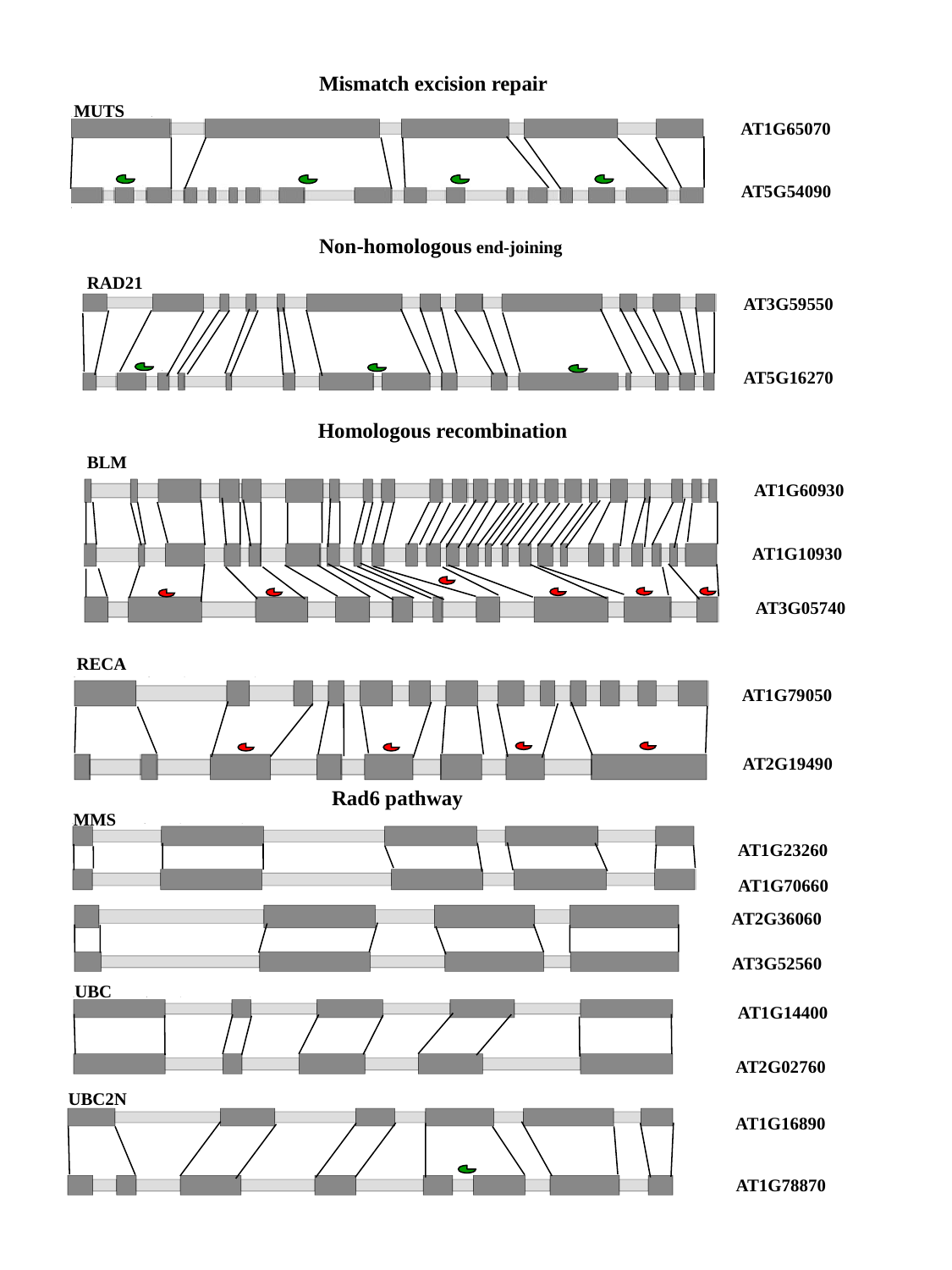

Mismatch excision repair
MUTS
AT1G65070
AT5G54090
Non-homologous end-joining
RAD21
AT3G59550
AT5G16270
Homologous recombination
BLM
AT1G60930
AT1G10930
AT3G05740
RECA
AT1G79050
AT2G19490
Rad6 pathway
MMS
AT1G23260
AT1G70660
AT2G36060
AT3G52560
UBC
AT1G14400
AT2G02760
UBC2N
AT1G16890
AT1G78870

## Slide 4
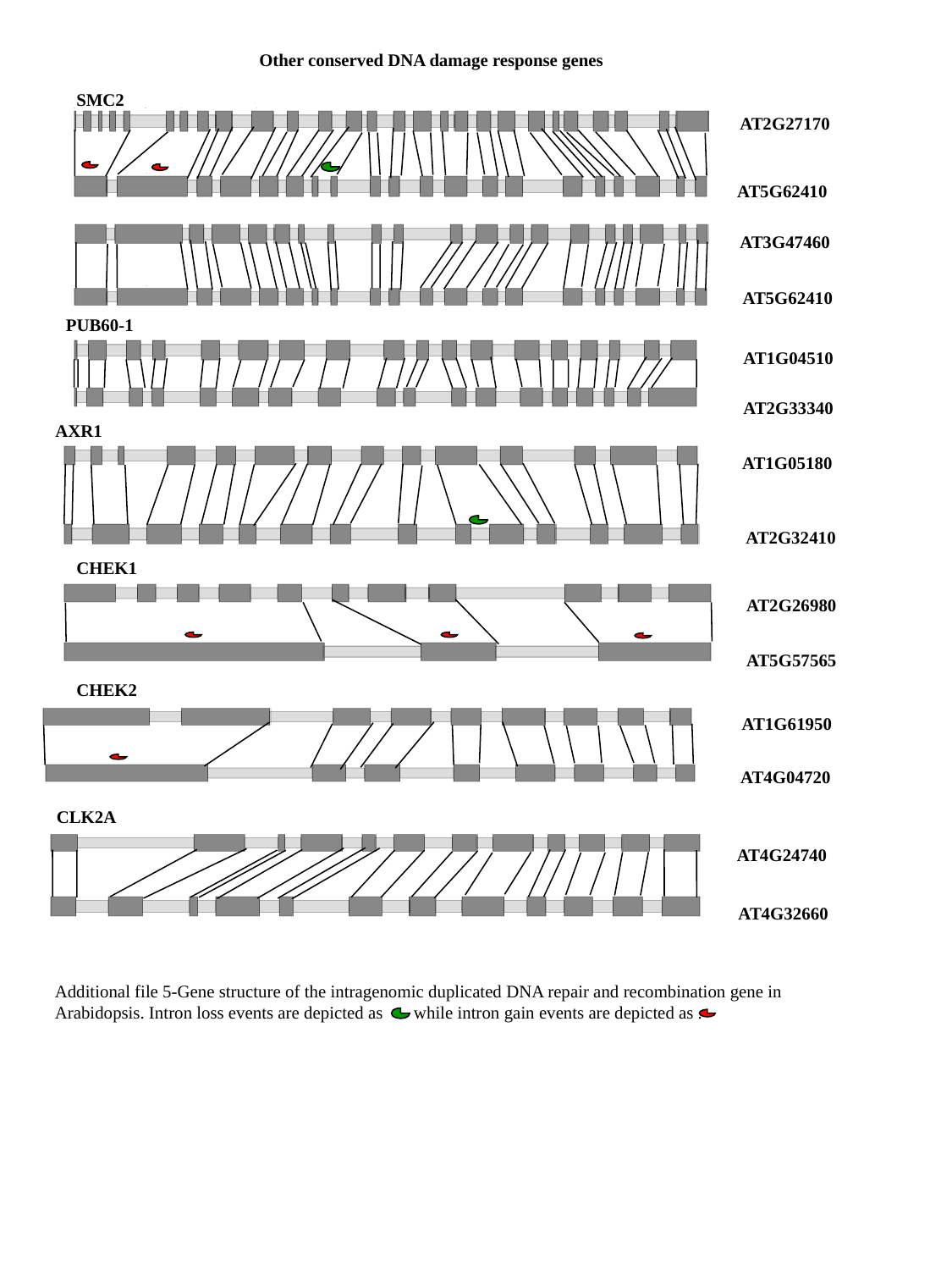

Other conserved DNA damage response genes
SMC2
AT2G27170
AT5G62410
AT3G47460
AT5G62410
PUB60-1
AT1G04510
AT2G33340
AXR1
AT1G05180
AT2G32410
CHEK1
AT2G26980
AT5G57565
CHEK2
AT1G61950
AT4G04720
CLK2A
AT4G24740
AT4G32660
Additional file 5-Gene structure of the intragenomic duplicated DNA repair and recombination gene in Arabidopsis. Intron loss events are depicted as while intron gain events are depicted as .
