# Supplementary material for: DNA repair and recombination in higher plants: insights from comparative genomics of arabidopsis and rice
Source: BMC Genomics. 2010 Jul 21;11:443. doi: 10.1186/1471-2164-11-443 (PMC3091640; doi:10.1186/1471-2164-11-443)
Supplement: Additional file 6 — Gene structure of the intragenomic duplicated DRR gene in rice. [file 1471-2164-11-443-S6.PPT]

## Slide 1
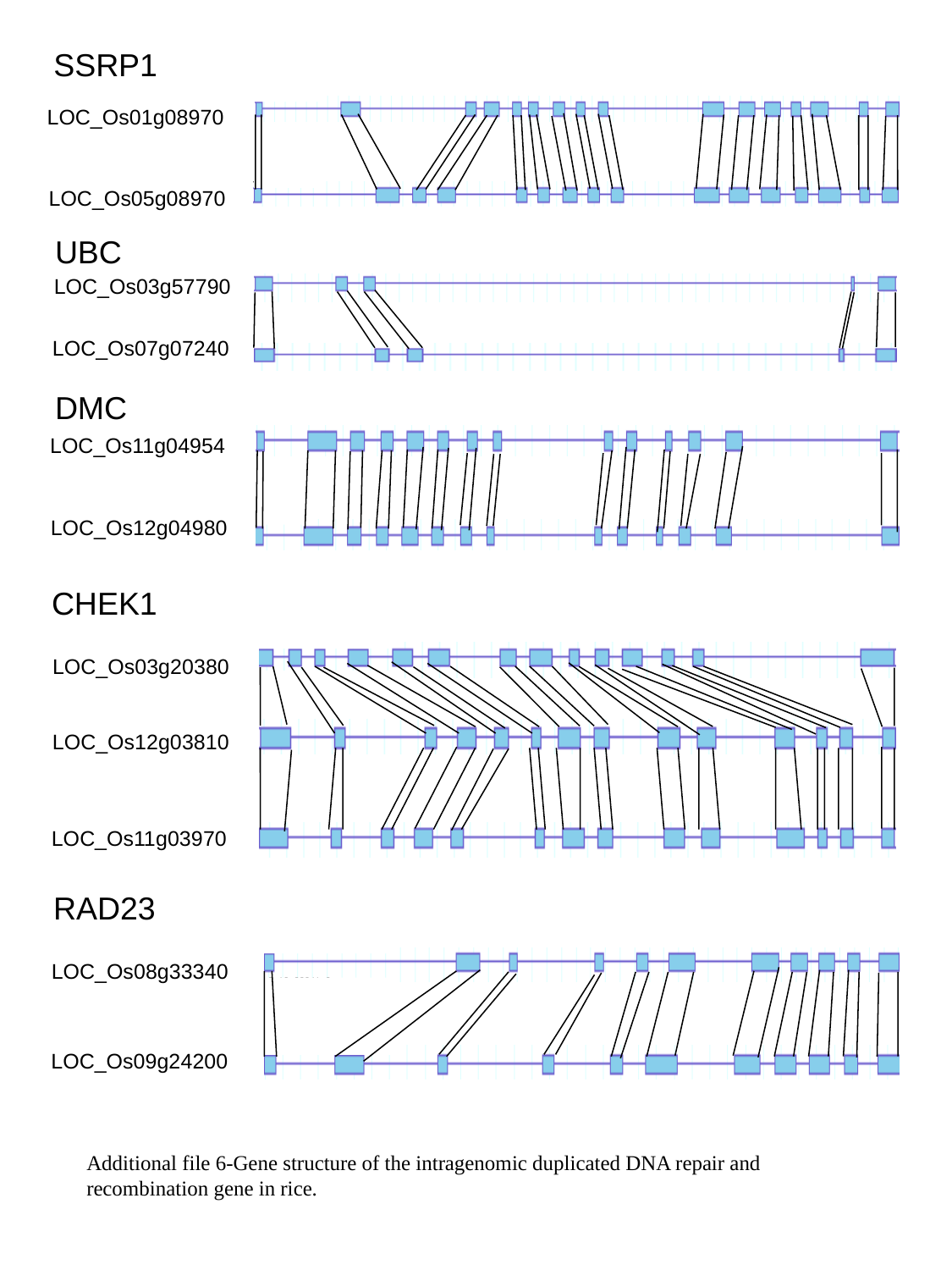

SSRP1
LOC_Os01g08970
LOC_Os05g08970
UBC
LOC_Os03g57790
LOC_Os07g07240
DMC
LOC_Os11g04954
LOC_Os12g04980
CHEK1
LOC_Os03g20380
LOC_Os12g03810
LOC_Os11g03970
RAD23
LOC_Os08g33340
LOC_Os09g24200
Additional file 6-Gene structure of the intragenomic duplicated DNA repair and recombination gene in rice.
