# Supplementary material for: DNA repair and recombination in higher plants: insights from comparative genomics of arabidopsis and rice
Source: BMC Genomics. 2010 Jul 21;11:443. doi: 10.1186/1471-2164-11-443 (PMC3091640; doi:10.1186/1471-2164-11-443)
Supplement: Additional file 7 — Gene structure of the intergenomic duplicated DRR gene in Arabidopsis and rice. [file 1471-2164-11-443-S7.PPT]

## Slide 1
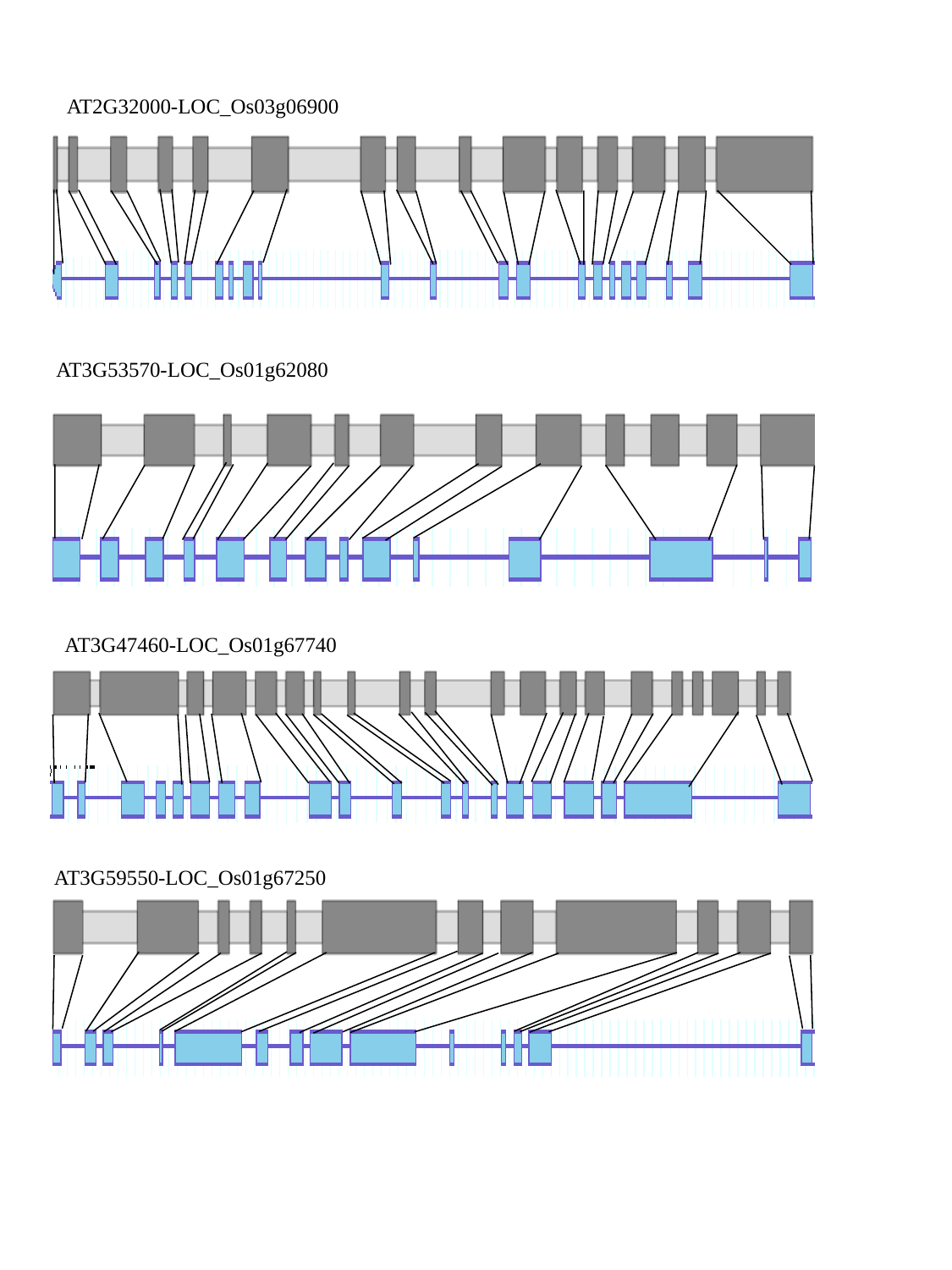

AT2G32000-LOC_Os03g06900
AT3G53570-LOC_Os01g62080
AT3G47460-LOC_Os01g67740
AT3G59550-LOC_Os01g67250

## Slide 2
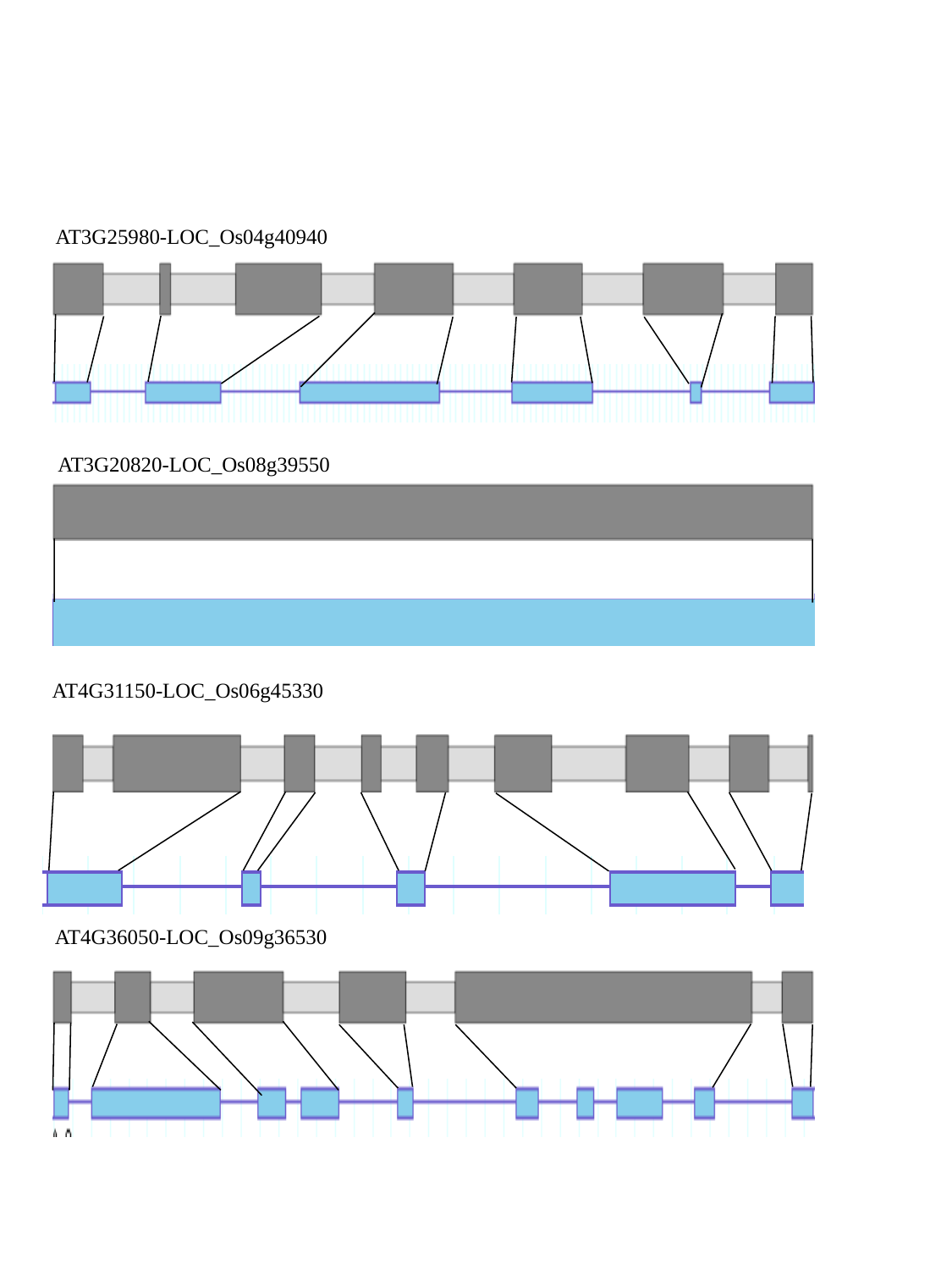

AT3G25980-LOC_Os04g40940
AT3G20820-LOC_Os08g39550
AT4G31150-LOC_Os06g45330
AT4G36050-LOC_Os09g36530

## Slide 3
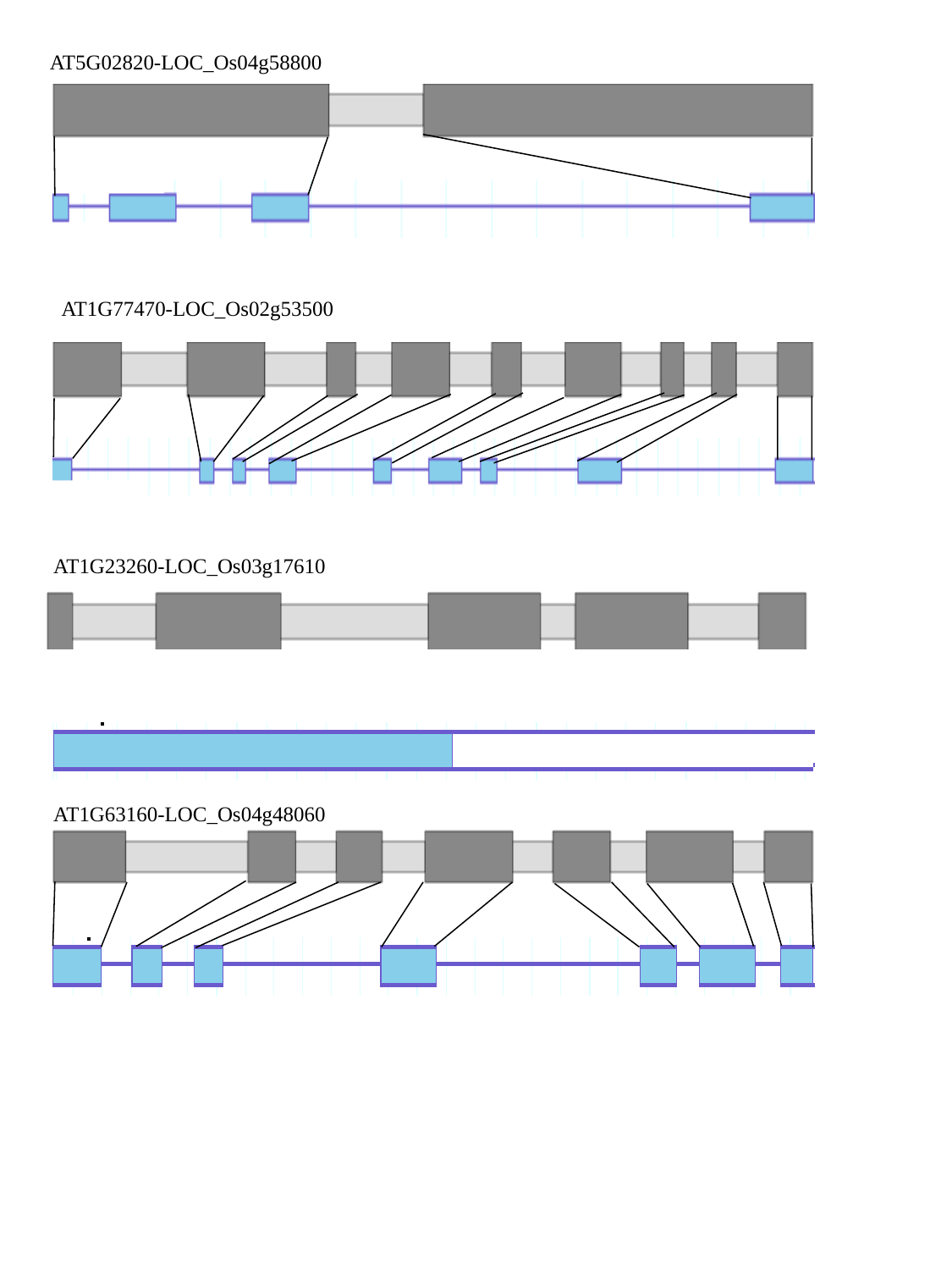

AT5G02820-LOC_Os04g58800
AT1G77470-LOC_Os02g53500
AT1G23260-LOC_Os03g17610
AT1G63160-LOC_Os04g48060

## Slide 4
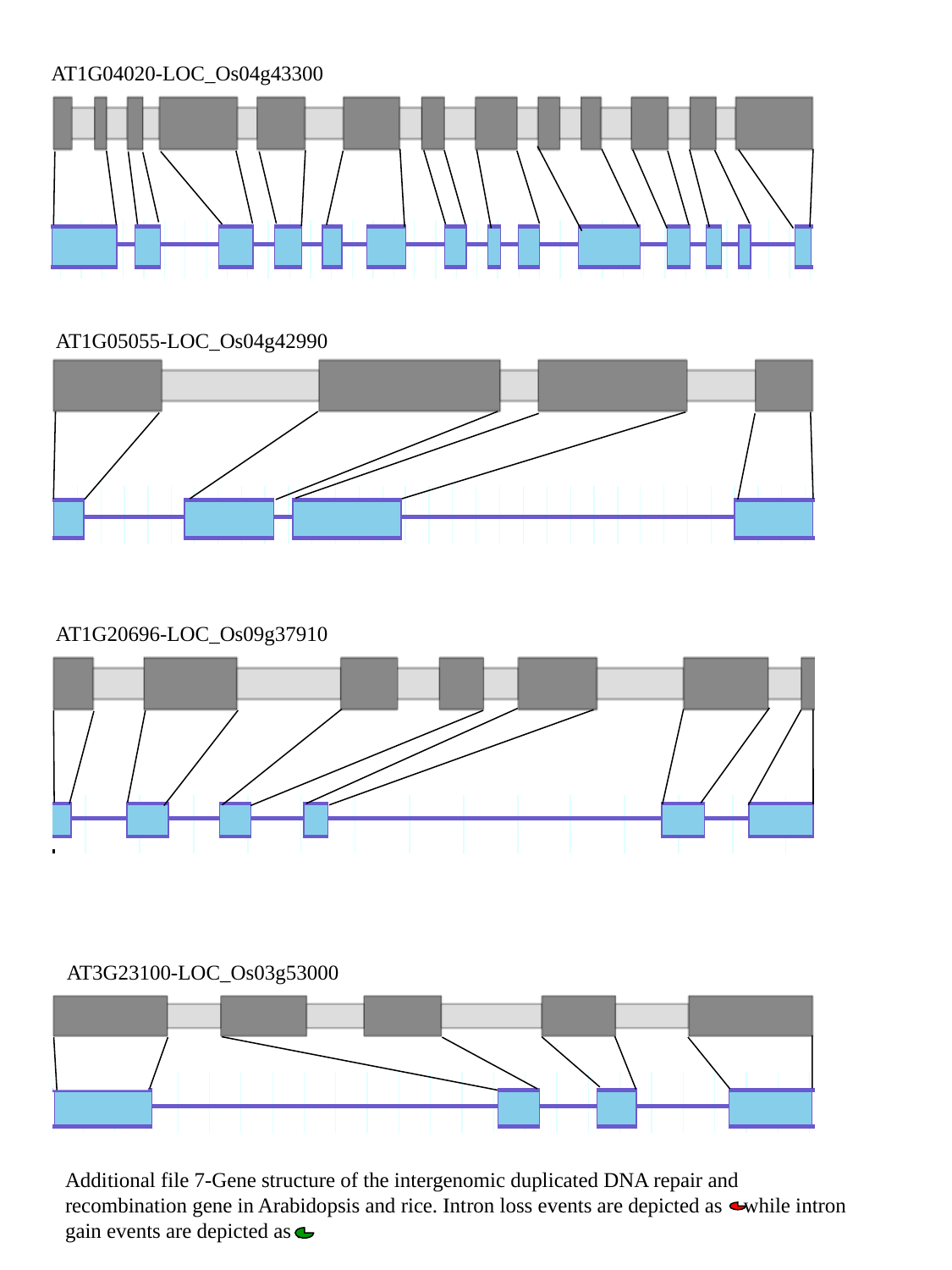

AT1G04020-LOC_Os04g43300
AT1G05055-LOC_Os04g42990
AT1G20696-LOC_Os09g37910
AT3G23100-LOC_Os03g53000
Additional file 7-Gene structure of the intergenomic duplicated DNA repair and recombination gene in Arabidopsis and rice. Intron loss events are depicted as while intron gain events are depicted as .
