# Supplementary material for: DNA repair and recombination in higher plants: insights from comparative genomics of arabidopsis and rice
Source: BMC Genomics. 2010 Jul 21;11:443. doi: 10.1186/1471-2164-11-443 (PMC3091640; doi:10.1186/1471-2164-11-443)
Supplement: Additional file 8 — Gene name, E-value, Ka value and Ks value of Intra and intergenomic duplication in rice and Arabidopsis DRR genes. [file 1471-2164-11-443-S8.DOC]

**Additional file 8A:** Intragenomic duplication in rice DNA repair and recombination genes.

| **Gene name** | **Locus_A** | **Locus_B** | **E-value** | **Ka** | **Ks** |
| --- | --- | --- | --- | --- | --- |
| SSRP1 | LOC_Os01g08970 | LOC_Os05g08970 | 7.00E-159 | 0.08 | 0.75 |
| UBC | LOC_Os03g57790 | LOC_Os07g07240 | 3.00E-27 | 0.01 | 0.62 |
| CHEK1 | LOC_Os03g20380 | LOC_Os12g03810 | 1.00E-136 | 6 | 1 |
| RAD23B | LOC_Os08g33340 | LOC_Os09g24200 | 0 | 8 | 1.01 |
| CHEK1 | LOC_Os11g03970 | LOC_Os12g03810 | 0 | 0 | 0.05 |
| DMC1 | LOC_Os11g04954 | LOC_Os12g04980 | 0 | 0.01 | 4 |

**Additional file 8B:** Intergenomic duplication in rice and Arabidopsis DNA repair and recombination genes.

| **Gene name** | **Arabidopsis locus** | **rice locus** | **E-value** | **Ka** | **Ks** |
| --- | --- | --- | --- | --- | --- |
| TOP3 | AT2G32000 | LOC_Os03g06900 | 1.00E-118 | 0.83 | -1 |
| CLK2B | AT3G53570 | LOC_Os01g62080 | 2.00E-40 | 5 | 2.91 |
| SMC2 | AT3G47460 | LOC_Os01g67740 | 1.00E-49 | 0.22 | 2.4 |
| RAD21.2 | AT3G59550 | LOC_Os01g67250 | 6.00E-98 | 0.92 | 2.77 |
| XRCC4 | AT3G23100 | LOC_Os03g53000 | 2.00E-70 | 0.44 | -1 |
| REV7 | AT3G25980 | LOC_Os04g40940 | 2.00E-64 | 0 | -1 |
| DRT100 | AT3G20820 | LOC_Os08g39550 | 1.00E-68 | 0.42 | 2.08 |
| FLJ35220 | AT4G31150 | LOC_Os06g45330 | 2.00E-42 | 0.41 | 1.45 |
| APE2 | AT4G36050 | LOC_Os09g36530 | 3.00E-43 | 0.55 | 1.92 |
| SPO11-3 | AT5G02820 | LOC_Os04g58800 | 2.00E-82 | 0.29 | -1 |
| RFC3 | AT1G77470 | LOC_Os02g53500 | 5.00E-148 | 7 | 3.01 |
| UEV1 | AT1G23260 | LOC_Os03g17610 | 1.00E-149 | 0.3 | -1 |
| RFC4 | AT1G63160 | LOC_Os04g48060 | 1.00E-92 | 1 | -1 |
| BARD1 | AT1G04020 | LOC_Os04g43300 | 1.00E-100 | 0.6 | 47 |
| GTF2H2 | AT1G05055 | LOC_Os04g42990 | 1.00E-100 | 0.22 | 1.71 |
| HMGB3 | AT1G20696 | LOC_Os09g37910 | 2.00E-148 | 0.29 | 19 |

**Additional file 8C:** Intragenomic duplication in Arabidopsis DNA repair and recombination genes.

| **Gene name** | **Locus_A** | **Locus_B** | **E-value** | **Ka** | **Ks** |
| --- | --- | --- | --- | --- | --- |
| **Base Exicision Repair** | |  |  |  |  |
| *MAGLP/AlkA* | AT1G19480 | AT1G75230 | 5.00E-173 | 0.15 | 0.98 |
|  |  | AT3G50880 | 2.00E-179 | 0.4 | 2.82 |
| Tag | AT1G15970 | AT1G80850 | 0 | 0.21 | 0.75 |
|  |  | AT1G75090 | 5.00E-63 | 0.54 | 1.9 |
|  |  | AT3G12710 | 1.00E-116 | 0.68 | -1 |
| *NTH* | AT1G05900 | AT2G31450 | 0 | 0.22 | 0.55 |
| DML1 | AT2G36490 | AT3G10010 | 4.00E-101 | 0.62 | 2.69 |
|  |  | AT5G04560 | 2.00E-52 | 0.64 | 2.69 |
| HMGB1 | AT3G51880 | AT4G35570 | 3.00E-124 | 0.43 | -1 |
|  |  |  |  |  |  |
| **Nucleotide excision repair (NER)** | | |  |  |  |
| CDK7 | AT1G18040 | AT1G67580 | 3.00E-127 | 0.63 | 3.12 |
|  |  | AT1G73690 | 1.00E-51 | 0.09 | 0.57 |
|  |  | AT3G48750 | 2.00E-179 | 0.51 | -1 |
|  |  |  |  |  |  |
| DDB1 | AT4G05420 | AT4G21100 | 3.00E-85 | 0.04 | 0.57 |
|  |  |  |  |  |  |
| LIG1 | AT1G08130 | AT1G49250 | 2.00E-33 | 0.14 | 0.39 |
|  |  |  |  |  |  |
| PCNA | AT1G07370 | AT2G29570 | 0 | 0.02 | 1.13 |
|  |  |  |  |  |  |
| RAD23B | AT1G16190 | AT1G79650 | 1.00E-54 | 0.1 | 0.6 |
|  |  |  |  |  |  |
| RAD23C | AT3G02540 | AT5G16090 | 3.00E-119 | 0.34 | 0.63 |
|  |  | AT5G38470 | 7.00E-115 | 0.41 | 1.49 |
|  |  |  |  |  |  |
| RPA1 | AT4G19130 | AT5G45400 | 7.00E-83 | 0.22 | 0.79 |
| RBX1 | AT3G42830 | AT5G20570 | 8.00E-61 | 0.08 | 1.21 |
|  | AT3G05870 | AT5G20570 | 3.00E-119 | 0.44 | -1 |
| **Homologous recombination (HR)** | | |  |  |  |
| BLM | AT1G10930 | AT1G60930 | 8.00E-132 | 0.16 | 0.56 |
|  |  | AT3G05740 | 2.00E-111 | 0.66 | -1 |
| RecA – *E. coli* | AT1G79050 | AT2G19490 | 0 | 0.63 | 4.21 |
| **Non-homologous end-joining (NHEJ)** | | |  |  |  |
| RAD21.3 | AT5G16270 | AT3G59550 | 1.00E-131 | 0.87 | 2.58 |
|  |  |  |  |  |  |
| **Rad6 pathway** |  |  |  |  |  |
| UBC1 | AT1G14400 | AT2G02760 | 2.00E-28 | 0.01 | 0.69 |
| UBE2N | AT1G16890 | AT1G78870 | 1.00E-54 | 0 | 0.55 |
| MMS2 | AT1G23260 | AT1G70660 | 4.00E-114 | 0.07 | 0.58 |
|  | AT2G36060 | AT3G52560 | 3.00E-80 | 0.03 | 0.47 |
| **Mismatch excision repair (MMR)** | | |  |  |  |
| *Muts like* | AT1G65070 | AT5G54090 | 1.00E-40 | 1.05 | 3.04 |
| *protein* |  |  |  |  |  |
| **Other conserved DNA damage response genes** | | | |  |  |
| CHEK1 | AT2G26980 | AT5G57565 | 8.00E-49 | 0.43 | -1 |
| CHEK2 | AT1G61950 | AT4G04720 | 5.00E-34 | 0.22 | -1 |
| CLK2A | AT4G24740 | AT4G32660 | 3.00E-27 | 0.3 | 4.79 |
| *PR19A/PUB60-1* | AT1G04510 | AT2G33340 | 0 | 0.1 | 0.63 |
| AXR1 | AT1G05180 | AT2G32410 | 0 | 0.11 | 0.66 |
| SMC2 | AT3G47460 | AT5G62410 | 3.00E-172 | 0.08 | 0.56 |
| SMC3 | AT2G27170 | AT5G62410 | 4.00E-64 | 1 | -1 |
