# Supplementary material for: DNA repair and recombination in higher plants: insights from comparative genomics of arabidopsis and rice
Source: BMC Genomics. 2010 Jul 21;11:443. doi: 10.1186/1471-2164-11-443 (PMC3091640; doi:10.1186/1471-2164-11-443)
Supplement: Additional file 9 — Accession numbers for Populous and Sorghum. [file 1471-2164-11-443-S9.DOC]

| PGDD accession number for Sorghum | JGI accession number for Sorghum |
| --- | --- |
| Sb02g024340 | jgi|Sorbi1|47850 |
| Sb04g005370 | jgi|Sorbi1|50036 |
| Sb10g009520 | jgi|Sorbi1|48972 |
| Sb07g023746 | jgi|Sorbi1|50439 |
| Sb01g010300 | jgi|Sorbi1|48682 |
| Sb04g011270 | jgi|Sorbi1|14857 |
| Sb02g020340 | jgi|Sorbi1|48742 |
| Sb06g033510 | jgi|Sorbi1|14921 |
| Sb09g021920 | gi|Sorbi1|50605 |
| Sb08g008620 | jgi|Sorbi1|50594 |
| Sb04g019820 | jgi|Sorbi1|50551 |
| Sb03g043060 | jgi|Sorbi1|50542 |
| Sb09g030210 | jgi|Sorbi1|47691 |
| Sb09g030220 | jgi|Sorbi1|47691 |

**Additional file 9A:** Accession numbers for Sorghum

| PGDD accession number for Sorghum | JGI accession number for Sorghum |
| --- | --- |
| Pt03g1717 | jgi|Poptr1_1|800 |
| Pt01g0173 | jgi|Poptr1_1|750 |
| Pt04g0545 | jgi|Poptr1_1|831 |
| Pt10g1103 | jgi|Poptr1_1|225 |
| Pt08g1112 | jgi|Poptr1_1|419 |
| Pt10g2335 | jgi|Poptr1_1|822 |
| Pt08g0223 | jgi|Poptr1_1|420 |
| Pt10g0928 | jgi|Poptr1_1|566 |
| Pt08g1251 | jgi|Poptr1_1|720 |
| Pt16g0636 | jgi|Poptr1_1|667 |
| Pt06g1340 | jgi|Poptr1_1|653 |
| Pt00028g0292 | jgi|Poptr1_1|282 |
| Pt00123g0078 | jgi|Poptr1_1|266 |
| Pt00029g0372 | jgi|Poptr1_1|826 |
| Pt03g0772 | jgi|Poptr1_1|757 |
| Pt09g0151 | jgi|Poptr1_1|804 |

**Additional file 9B:** Accession numbers for Populous
