# Supplementary material for: DNA repair and recombination in higher plants: insights from comparative genomics of arabidopsis and rice
Source: BMC Genomics. 2010 Jul 21;11:443. doi: 10.1186/1471-2164-11-443 (PMC3091640; doi:10.1186/1471-2164-11-443)
Supplement: Additional file 10 — Additional methods. [file 1471-2164-11-443-S10.DOC]

**Supplementary methods:-**

Gene structure for different gene for Arabidopsis and rice was predicted by using the Scipio (http://www.webscipio.org/webscipio/webscipio2) and TIGR (http://rice.plantbiology.msu.edu/cgi-bin/gbrowse/rice) server respectively. Whole genome sequence for *Populus* and *Sorghum* were downloaded from the website of Joint Genome Institute database (http://genome.jgi-psf.org/)Although in main text we have used the naming pattern of the authors of PGDD (http://chibba.agtec.uga.edu/duplication) as it was more informative. Chromosomal location of different DNA repair and recombination genes for Arabidopsis were mapped by TAIR’s Chromosome map tool utility (<http://www.arabidopsis.org/jsp/ChromosomeMap/tool.jsp>). We modellled the 3D structure for the conserved domains for AtRECQL4A and AtSMC2a as described previously by Singh et al. (Singh et al. 2008) using the MODELLER9v6 (Sali and Blundell 1993). Protein structure quality was checked by PROCHECK (Laskowski et al. 1993).

Odronitz, H. Pillmann, O. Keller, Waack S, Kollmar M (2008) WebScipio: An online tool for the determination of gene structures using protein sequences. BMC Genomics 9:422.

Singh SK, Roy Choudhury S, Roy S, Sengupta DN (2008) Sequential, Structural, and Phylogenetic Study of BRCT Module in Plants. J Biomol Struct Dyn 26:235-245.

Sali A, Blundell TL (1993) Comparative protein modelling by satisfaction of spatial restraints. J. Mol. Biol. 234: 779-815.

Laskowski R A, MacArthur M W, Moss D S, J M Thornton (1993). PROCHECK: a program to check the stereochemical quality of protein structures. J. Appl. Cryst*.*, 26: 283-291.
